# Narrow funnel-like interaction energy distribution is an indicator of specific protein interaction partner

**DOI:** 10.1016/j.isci.2023.106911

**Published:** 2023-05-20

**Authors:** Juyoung Choi

**Affiliations:** 1 Department of Life Science, Sogang University, Seoul 04017, South Korea

**Keywords:** Artificial intelligence, Biochemistry, Biochemistry methods, Machine learning, Protein

## Abstract

Protein interaction networks underlie countless biological mechanisms. However, most protein interaction predictions are based on biological evidence that are biased to well-known protein interaction or physical evidence that exhibits low accuracy for weak interactions and requires high computational power. In this study, a novel method has been suggested to predict protein interaction partners by investigating narrow funnel-like interaction energy distribution. In this study, it was demonstrated that various protein interactions including kinases and E3 ubiquitin ligases have narrow funnel-like interaction energy distribution. To analyze protein interaction distribution, modified scores of iRMS and TM-score are introduced. Then, using these scores, algorithm and deep learning model for prediction of protein interaction partner and substrate of kinase and E3 ubiquitin ligase were developed. The prediction accuracy was similar to or even better than that of yeast two-hybrid screening. Ultimately, this knowledge-free protein interaction prediction method will broaden our understanding of protein interaction networks.

## Highlights

• Proteins that interact show narrow funnel-like interaction energy distribution
• Protein interaction prediction using energy distribution is accurate across species
• This method can predict substrates of kinase and EUL
• Deep learning model can predict protein interaction using energy distribution

## Graphical abstract

**Figure:**
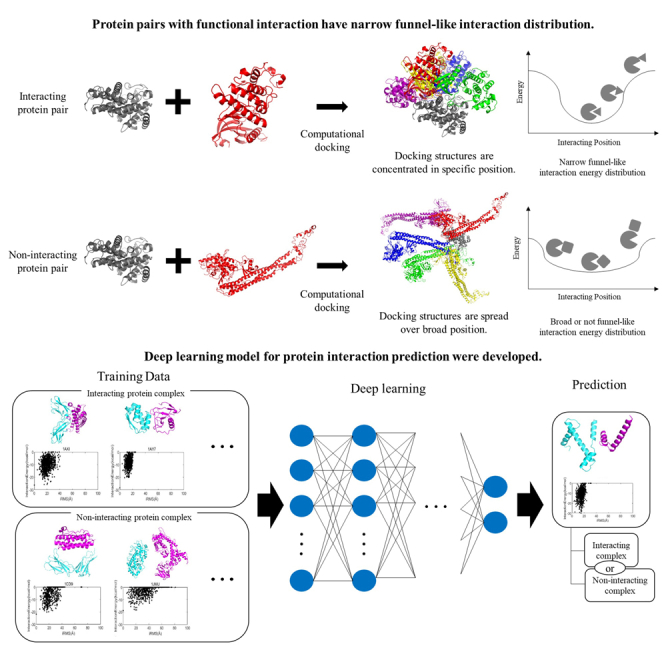

## Introduction

Most biological activities occur through complex biomolecular interaction networks. After the genome sequencing of several tens of thousands of organisms, understanding biomolecule networks beyond the individual genes is becoming increasingly important. In particular, protein-protein interactions (PPI) are among the most diverse biomolecular networks and the center of system biology. Several experimental techniques, such as yeast two-hybrid screening, coimmunoprecipitation, affinity-chromatography, tandem affinity purification-mass spectroscopy, etc., have been developed to identify PPI.^1^ However, with the rapid advancement of proteome identification, experimental results cannot keep up with the potential number of protein combinations. Recently, 200 million proteins in various organisms were predicted or identified.^2^ Meanwhile, the international consortium curated only approximately 1 million PPIs from literature and datasets.^3^^,^^4^ Moreover, among the identified PPI, approximately 70% are *Homo sapiens*-related.^4^ Most PPI might still be unidentified. Therefore, *in silico* methods, as well as high throughput experimental approaches, have been developed to broaden our PPI-related knowledge.^5^

*In silico* PPI prediction methods utilize biological, structural, and physical results. Biological evidence includes gene or domain fusion,^6^^,^^7^ gene neighborhood,^6^^,^^8^ interolog,^9^ coexpression,^6^^,^^10^ coevolution,^11^ and phylogenetic similarity.^6^^,^^12^ Although biological result-based PPI predictions have a powerful predictive ability, in addition to being applicable on a large scale, they are highly biased to well-known PPI. Except for PPIs with rare molecular features (gene or domain fusion and gene neighborhood), omics scale interspecies data are required or at least similar protein interactions in other species should be identified to predict PPI using biological results.^5^^,^^13^ Structural and physical evidence-based PPI prediction utilizes protein-protein interface templates,^14^ interaction energy,^13^^,^^15^ and shape complementarity.^15^^,^^16^ Template-based PPI predictions require known PPIs with similar interface structures.^14^^,^^17^ All these knowledge-based PPI prediction are not enough to discover new PPI, since most PPI might still be unknown. Conversely, PPI predictions with interaction energy and shape complementarity are independent of accumulated large-scale data and known PPIs. However, shape complementarity is different for each PPI type. Obligate PPIs, which are stable only when interacting with each other, have higher shape complementarity, although transient PPIs exhibit low shape complementarity.^5^^,^^18^ Moreover, PPI prediction using interaction energy require a lot of computational power and is not suitable for large scale prediction. Especially, protein docking programs, which calculate the possible direction of interaction and interaction energy, have a high computational cost.^5^^,^^15^ Transient PPIs with weak interactions are difficult to predict using interaction energy.^15^ However, most PPIs are weak, and weak PPIs play a pivotal role in protein interaction networks.^19^ Therefore, high computational cost and the inability to predict transient PPIs with weak interactions represent the major drawbacks of protein interaction partner prediction using *in silico* protein docking.

One of the most difficult PPIs to predict is that of enzyme-specific interaction partners, such as substrates, inhibitors, and regulators, or signaling pathway interactions. Their diversity of complicated signaling pathways^20^ makes biological evidence- and structural template-based PPI prediction difficult. Furthermore, due to weak and transient interaction properties, interaction energy and shape complementarity are not useful for the prediction of these PPIs.^15^^,^^18^ However, understanding PPIs in signaling pathways, such as the interactions of kinases or E3 ubiquitin ligase (EUL) with their interaction partners, is one of the most crucial parts of biology. In mammals, kinases and ubiquitination-related enzymes are the most and the fifth most abundant enzymes in signaling pathways, respectively.^20^ Moreover, in plants, approximately 5% of the protein-coding genes in rice and *Arabidopsis* encode kinases and EULs.^21^^,^^22^^,^^23^ Despite the importance of kinases and EULs, most interaction partner predictions of kinases and EULs are mostly based on biological evidence, which require identified PPI with similar amino acid sequences or domains.^24^^,^^25^^,^^26^^,^^27^^,^^28^^,^^29^^,^^30^ Moreover, because of the lack of identified PPIs, most prediction tools are limited to human kinases or EULs.^24^^,^^26^^,^^27^^,^^28^^,^^29^^,^^30^ Therefore, in most cases, no high-accuracy PPI prediction tools are available for kinases and EULs.

**Figure 1:**
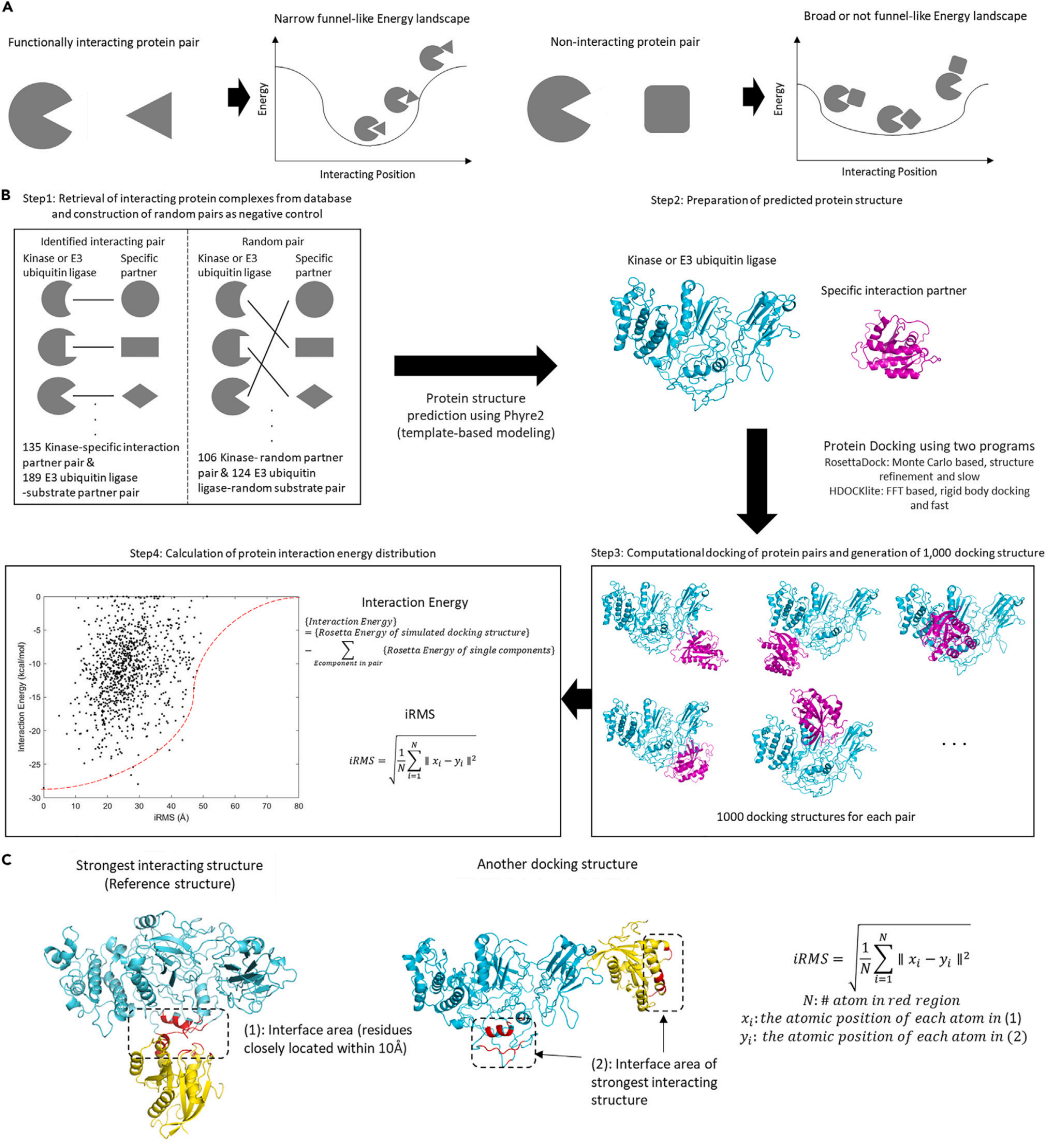
Narrow funnel-like interaction energy distribution hypothesis and workflow for interaction energy distribution analysis (A) Schematic diagram of narrow funnel hypothesis. Specific interactions might have narrow funnel-like interaction energy distribution. (B) Schematic diagram of interaction energy distribution analysis workflow is shown. Each step of interaction energy distribution analysis is described. Dashed line in Figure 1B was drawn as a range including all points except for the five outliers farthest from the origin (0,0) to visually represent a narrow funnel shape in the interaction energy distribution. (C) Introduction of redefined iRMS. Residues of the interface area in the strongest interacting structure are colored in red.

In this study, I suggest a new, interaction energy distribution-based PPI prediction strategy. PPIs with a specific interaction partner, such as PPIs of kinase and EULs, exhibit the specificity to maintain sophisticated signaling pathways. For instance, in humans, kinase phosphorylates specific phosphorylation sites ranging from one to hundreds among approximately 700,000 potential phosphorylation sites.^31^ Moreover, most regulators and inhibitors act when they interact with kinases only in a specific orientation.^32^^,^^33^ Although these PPIs are weak, the specificity of these interactions observed in a specific orientation distinguishes them from non-interacting protein pairs.^31^^,^^34^ Based on this specificity, it was hypothesized that an interaction energy diagram of PPI with specific interaction partners would display a narrow funnel-like landscape, exhibiting a stable state in a specific orientation (Figure 1A). In this study, using Rosetta energy function, knowledge (or Boltzmann relation)-based macromolecular energy function,^35^ the PPI interaction energy distribution was calculated.

Although there are considerable differences of interaction energy distributions between interacting and non-interacting protein complexes, setting a classification criterion with high accuracy is difficult. Recent advances in deep learning have made it possible to extract hidden features from complicated data and classify them.^36^ Training using interaction energy distribution data of interacting and non-interacting protein complex can be employed to classify the interaction energy distribution of unknown protein pairs into interacting or non-interacting protein interaction energy distribution. This binary classification problem has been successfully resolved in various fields using machine learning.^37^ In this study, deep learning models were developed to predict general protein interaction partners and substrates of kinase and EUL.

## Results

### Workflow for the interaction energy distribution of kinases/E3 ubiquitin ligases and their interaction partners

To analyze the interaction energy landscape of kinases/EULs and their interaction partners, I retrieved 135 human kinase-interaction partners, including regulator, inhibitor, and substrate, and 189 human EUL-substrate pairs randomly choosen from known databases.^3^^,^^38^^,^^39^ Some protein pairs were randomly selected from databases because docking programs are too computationally expensive to calculate all protein pairs in databases. However, various types of kinases and EULs were selected to prevent data bias. For example, EUL-substrate pair include RING, HECT, Cullin based EUL complex and kinase-substrate pair include kinases with various functions such as cell cycle control, immune signaling, and hormone signaling with 19 functional domains (Data S1). To construct negative controls, I randomly paired kinases and EULs with different partners with no evidence of the existence of functional interactions in these pairs. Randomly paired partners might later turn out to be real interaction partners, but most likely they would not be interaction partners. Therefore, as a negative control, 106 kinase- and 124 EUL-random partner pairs were constructed (Figure 1B, Data S1).

Using Phyre2, a server for template-based structure modeling,^40^ full predicted structures of kinases, EULs, and their partners were obtained (Data S2). Because of computational costs, the calculation of every possible protein docking structure is nearly impossible. Therefore, 1,000 possible docking structures per pair were sampled using two docking programs (Table 1, Figure 1B). RosettaDock uses the Monte-Carlo algorithm with a knowledge-based energy function^35^ and includes the structure refinement of the docking structures.^41^ HDOCKlite uses the fast Fourier transform (FFT) algorithm with a shape-based scoring function and does not include structure refinement.^42^

**Table 1:** Docking programs for possible docking structure generation

	RosettaDOCK	HDOCKlite
Algorithm	Monte-Carlo	Fast-Fourier Transform
Scoring	Knowledge-based Energy Function	Shape-based Scoring Function
Structure Refinement	O	X (rigid body)
Speed	Slow^a^	Fast^b^
Ref.	Chaudhury et al.^41^	Yan et al.^42^

a Several hours ∼ several days per interaction pair in personal computer (Intel Core i7-10700K 3.80 GHz with 8 cores and 32 GB of RAM).

b Several minutes ∼ few hours per interaction pair in personal computer (Intel Core i7-10700K 3.80 GHz with 8 cores and 32 GB of RAM).

To analyze the interaction energy landscape (Figure 1A), I calculated the interaction energy with Rosetta energy and the following equation:

InteractionEnergy={Rosettaenergyofsimulateddockingstructure}

$$-\sum_{Each\;component\;in\;pair}\left\{Rosetta\;energy\;of\;single\;components\right\}$$

As every theoretical protein energy exhibits differences compared with the experimental data, I ignored energy change because of structure change and considered only the affinity to minimize error. To indicate interacting positions, I used interface root-mean-square deviation (iRMS).^43^ Originally, iRMS was developed to compare simulated docking and native structures.^44^ In this study, I slightly changed iRMS. Instead, of the native structure, I set the strongest interacting structure as a reference structure (Figure 1C). The redefined iRMS was as follows:

$$iRMS=\sqrt{\frac{1}{N}\sum_{i=1}^{N}{∥x_{i}-y_{i}∥}^{2}}$$

N:thenumberofbackboneatomofinterfaceresidues

$$x_{i}:the\;position\;of\;i\;th\;backbone\;atom\;of\;interface\;residues\;in\;docking\;structure$$

$$y_{i}:the\;position\;of\;i\;th\;backbone\;atom\;of\;interface\;residues\;in\;the\;strongest\;interacting\;structure$$

As Figure 1C shows, in the strongest interacting structure, residues closely located within 10 Å were set as interface residues. In different docking structures, the same residues were in different positions. iRMS is the root-mean-square deviation of the backbone atoms of interface residues in two structures. For every docking structure generated from each enzyme-interaction and random partner pairs using two docking programs, interaction energy and iRMS were analyzed and plotted (Data S3 and Figures S1–S4: generated using RosettaDock; Data S4 and Figures S5–S8: generated using HDOCKlite). As HDOCKlite does not include structure refinements, the interaction energy calculation indicates that most docking structures with HDOCKlite were unfavorable (interaction energy > 0 kcal/mol). However, affinity comparison would be possible by comparing the interaction energies.

### Narrow funnel-like interaction energy distribution of kinases/EULs and their specific interaction partners

**Figure 2:**
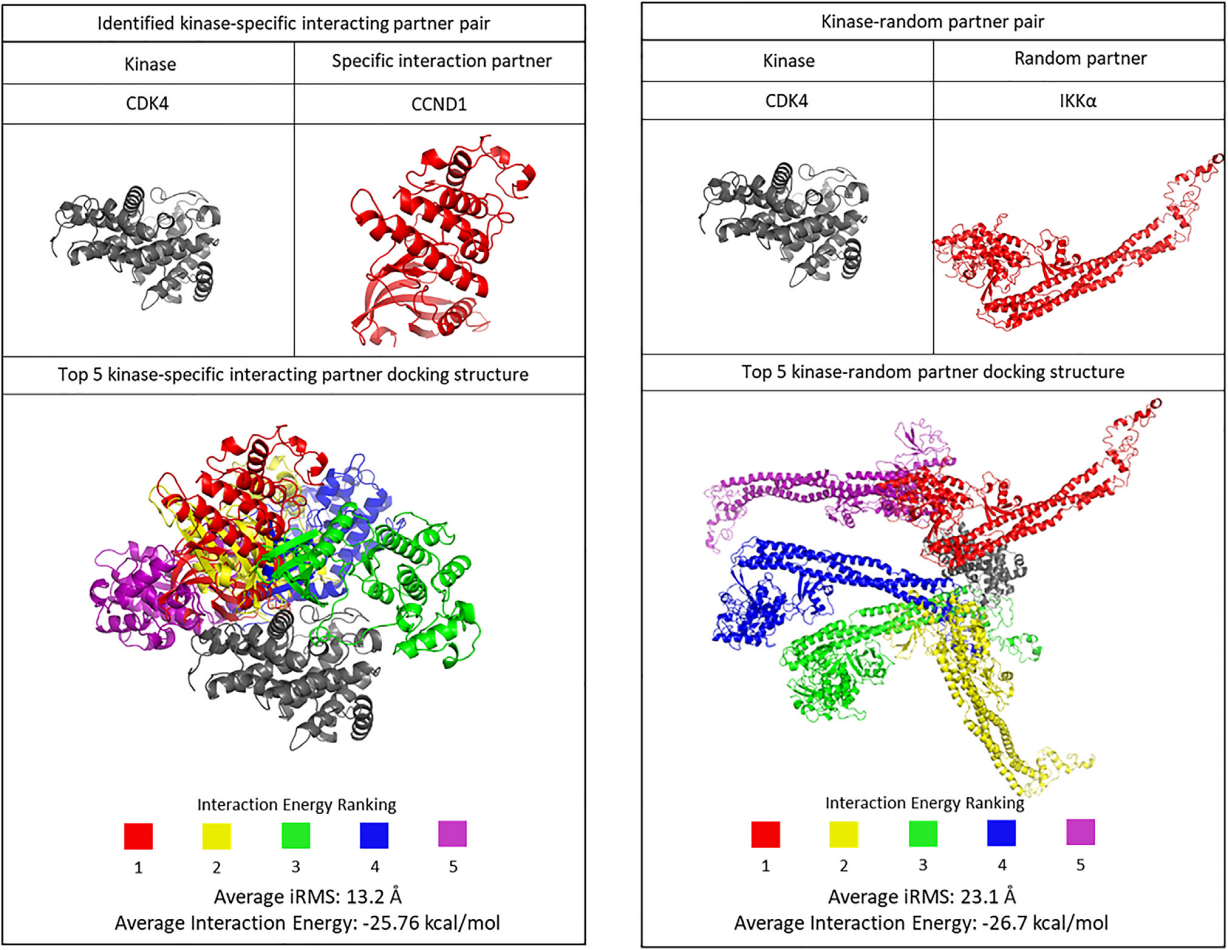
Structural meaning of narrow funnel-like interaction energy distribution The five strongest interacting structures of CDK4-CCND1 (identified specific interaction pair) and CDK4-IKKα (random pair). To avoid confusion, CDK4 in each pair is set in the same position and CDK4 in the strongest interacting structure is represented in gray. Each partner is colored in rainbow color in the order of interaction energy.

To illustrate the structural meaning of the narrow funnel-like interaction energy distribution, Figure 2 shows the interactions between cyclin-dependent kinase 4 (CDK4) and its specific/random partners. It is well-known that CDK4 interacts with G1/S-specific cyclin-D1 (CCND1) and mediates cell cycle progression (G1-to-S phase).^45^ Inhibitor of nuclear factor kappa-B kinase subunit alpha (IKKα) is part of the IKK complex and phosphorylates inhibitor of nuclear factor kappa-B.^46^ Then, the activated nuclear factor kappa-B mediates immune response, inflammation, and apoptosis.^47^ CDK4 randomly paired with IKKα and there is no literature and dataset have identified that they form a functional PPI. Using RosettaDock, 1,000 docking structures of CDK4-CCND1 and CDK4-IKKα pairs were established. Then, by calculating the interaction energy, the top five strongest docking structures have been summarized in Figure 2. The CDK4-CCND1 pair, an identified interaction pair, interacts in similar positions. However, the CDK4-IKKα pair, a random pair, shows scattered interacting positions on the surface of CDK4 and IKKα. Although the average interaction energies are similar and a little higher in the CDK4-IKKα pair, the average iRMSs of CDK4-CCND1 (functionally interacting pair) complex was significantly smaller than CDK4-IKKα (random pair).

**Figure 3:**
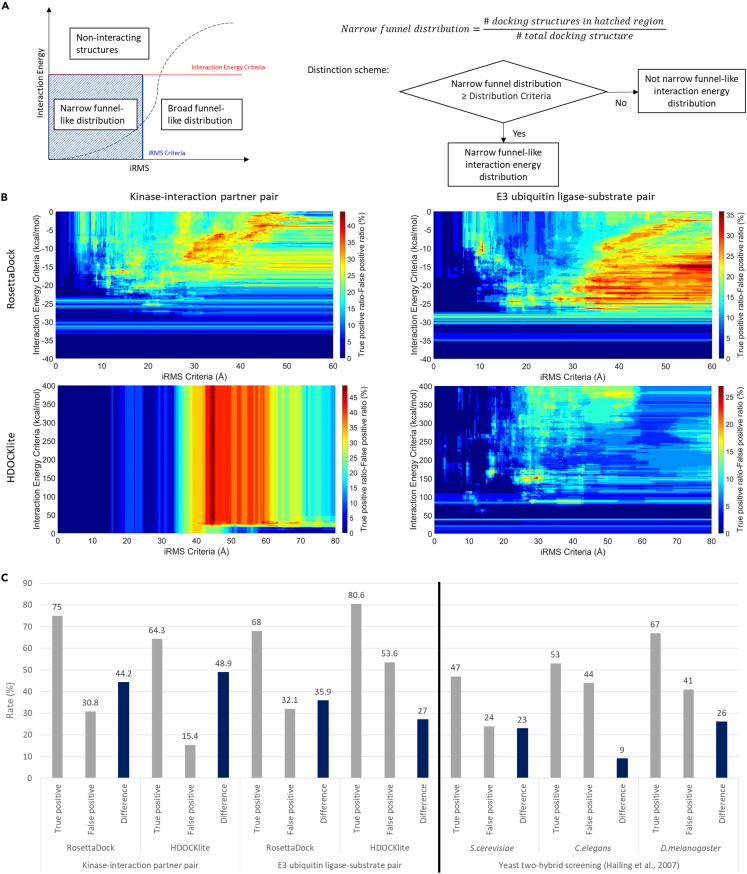
Quantitative analysis of narrow funnel-like interaction energy distribution and accuracy of predicting protein interactions by searching narrow funnel-like interaction distribution (A) Scheme of the strategy to distinguish narrow funnel-like interaction energy distributions. (B) How well narrow funnel interaction energy distribution can distinguish specific interaction partners for each criteria. Among whole interaction pairs, pairs with high-accuracy prediction results (>70% region with high confidence) and relatively small proteins (≤700 residues) were analyzed. Difference ratios between true-positive (identified interacting protein complex with narrow funnel-like interaction energy distribution) and false-positive (random pair with narrow funnel-like interaction energy distribution) for each criteria are shown on heatmap. Due to graphical representation limitations, only the maximum ratio differences are shown for each distribution criteria. (C) Rate comparisons for finding specific interaction partners using the narrow funnel and yeast two-hybrid screening methods. Comparison of genome-scale yeast two-hybrid analysis for three species.^49^ The criteria for finding narrow funnel-like energy distribution are described in Table S2.

For the quantitative analysis of the interaction energy distribution, I devised a strategy to distinguish narrow funnel-like interaction energy distribution (Figure 3A). Docking structures with weak interaction are regarded as functionally non-interacting structure. Docking structures with strong interaction but large iRMS indicate broad funnel-like interaction energy distribution. Therefore, I only had to consider docking structure ratios with strong interactions and small iRMS. I defined a score (narrow funnel distribution) for the distinction of narrow funnel-like distribution (Figure 3A). If the narrow funnel distribution was bigger than or equal to certain distribution criteria, this interaction energy distribution was determined as exhibiting narrow funnel-like distribution. Then, only three criteria (interaction energy, iRMS, and distribution criteria) had to be decided (Figure 3A).

As per my hypothesis, if a protein pair with specific interacting partners showed narrow funnel-like interaction energy distribution, specific partners could be predicted with interaction energy distribution analysis by taking specific criteria. Therefore, I calculated the difference between the true-positive (identified pair with narrow funnel-like interaction energy distribution) and false-positive (random pair with narrow funnel-like interaction energy distribution) ratios for each criterion. As per the hypothesis, the narrow funnel distribution of the functionally interacting protein complex was higher than that of random protein pairs in the case of most criteria.

For whole protein pairs, docking structures generated by RosettaDock, HDOCKlite showed up to 11.9%–20.3% difference of true- and false-positives (Figure S9). Interaction energy, iRMS, and distribution criteria at the maximum points are described in Table S1. As the interaction calculation depends on structure accuracy, I filtered out low-accuracy-predicted structures with less than 70% region with high confidence (>90% confidence)^40^ and analyzed the remaining pairs with high-accuracy structure prediction (identified kinase-interaction pair: n = 56 for RosettaDock, n = 58 for HDOCKlite; random kinase pair: n = 52 for RosettaDock, n = 54 for HDOCKlite; identified EUL pair: n = 59 for RosettaDock, n = 70 for HDOCKlite; random EUL pair: n = 62 for RosettaDock and HDOCKlite). The docking structures, generated by RosettaDock and HDOCKlite, showed up to 17.9%–26.4% difference of true- and false-positives (Figure S10). Interaction energy, iRMS, and distribution criteria at the maximum point are described in Table S2.

As protein global docking works well for small proteins,^48^ I again filtered out pairs with large proteins (>700 residues) and analyzed the remaining pairs with high-accuracy structure prediction and relatively small proteins (identified kinase-interaction pair: n = 28 for RosettaDock and HDOCKlite; random kinase pair: n = 26 for RosettaDock and HDOCKlite; identified EUL pair: n = 25 for RosettaDock, n = 36 for HDOCKlite; random EUL pair: n = 28 for RosettaDock and HDOCKlite). For the kinase pairs, the docking structures generated by RosettaDock and HDOCKlite showed up to 44.2% and 48.9% difference of true- and false-positives, respectively. For the EUL pairs, the docking structures generated by RosettaDock and HDOCKlite showed up to 35.9% and 27% difference of true- and false positives, respectively (Figure 3B). Interaction energy, iRMS, and distribution criteria at the maximum points are described in Table S3. In order to investigate the effectiveness of finding specific interaction partners using interaction energy distribution, true- and false-positive rates were compared with those of the yeast two-hybrid screening approach. True- and false-positive rates from genome-scale yeast two-hybrid trials in three species were compared.^49^ Whole pairs and pairs with high-accuracy structure predictions had similar accuracy to those of the yeast two-hybrid screening (Figures S9 and S10). Pairs with high-accuracy structure predictions and relatively small proteins showed significantly better results than those of the yeast two-hybrid screening approach (Figure 3C).

### Interaction energy distribution of other protein complexes

As kinase and EUL PPIs represent only a small part of the whole PPIs, I investigated the interaction energy distribution in other protein complexes. I retrieved 183 curated experimentally determined protein complex structures randomly choosen from the IntAct database,^4^ then analyzed the PPI between the interacting chains with the interface area in the protein structure using RosettaDock (see Figure 1B and Data S5). Some protein complexes were randomly selected from IntAct database because of computational cost. However, various protein complexes in various species were selected to prevent bias. To confirm that the protein interaction energy distribution analysis method is species-independent, protein complexes from 66 species including animals, plants, fungi, protozoan, bacteria and viruses were extensively analyzed. Moreover, to confirm whether it can be applied to various protein complexes, various protein complexes including cytoskeletal protein complex, enzyme-substrate complex, receptor complex, immune protein complex, etc (Data S5). The interaction energy and iRMS distribution of the protein complexes are plotted in Figure S11. Moreover, I retrieved 110 experimentally determined protein structures, which were unlikely engaged in direct interactions randomly chosen from the Negatome 2.0 database as negative controls,^50^ then analyzed the PPI using RosettaDock (see Figure 1B and Data S5). The interaction energy and iRMS distribution of the protein complexes are plotted in Figure S12.

**Figure 4:**
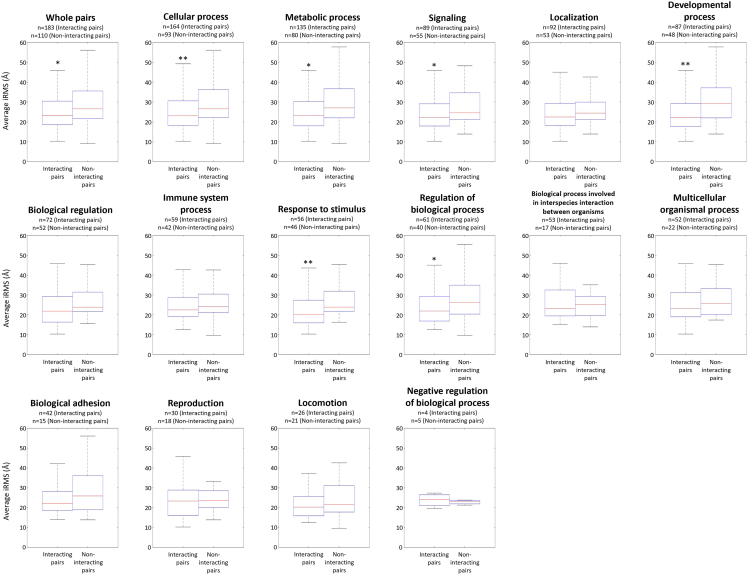
Distribution of average iRMS of interacting protein complexes and non-interacting protein pairs Distribution of average iRMSs of 1,000 docking structures of interacting protein complexes and non-interacting protein pairs are shown using box-and-whisker plot. Protein pairs divided according to the functional annotation with level-2 gene ontology distribution from Generic GO slim. Each related biological process in level-2 gene ontology from Generic GO slim and the number of protein pairs are notated. Significant differences of average iRMS with 95% confidence using Student’s *t* test are marked with asterisks and 99% confidence marked with double asterisks.

To confirm whether other protein complexes showed narrow funnel-like interaction energy distribution, I calculated the iRMS averages of simulated docking structures. As per the hypothesis (Figure 1A), the average iRMS of the interacting protein complexes were significantly smaller than the average iRMS of non-interacting protein pairs (95% confidence using Student’s *t* test, Figure 4). Furthermore, I functionally annotated these proteins according to level-2 gene ontology distribution from Generic GO slim,^51^ then divided the protein pairs according to the related biological processes and calculated the average iRMS for each part (Figure 4). Almost every division showed smaller average iRMS of the interacting complexes than those of the non-interacting pairs, except for the negative regulation of the biological processes. In particular, protein pairs related to cellular, metabolic, and developmental processes, signaling, response to stimuli, and biological process regulation showed significantly smaller average iRMSs than non-interacting protein pairs (95% confidence using Student’s *t* test). These processes are well-known to display molecular cascades with specific PPIs. Among those, cellular process-, developmental process-, and response to stimuli-related proteins pairs showed higher differences (99% confidence using Student’s *t* test). In particular, developmental process-related proteins pairs showed the biggest differences of iRMSs between the interacting complexes and non-interacting pairs (median of average iRMS of the interacting complexes: 22.37 Å, median of average iRMS of non-interacting pairs: 29.6 Å). This result might be related to developmental processes controlled by complex molecular cascades and networks with specific PPIs.^52^

### Deep learning model to predict protein interaction partners

**Figure 5:**
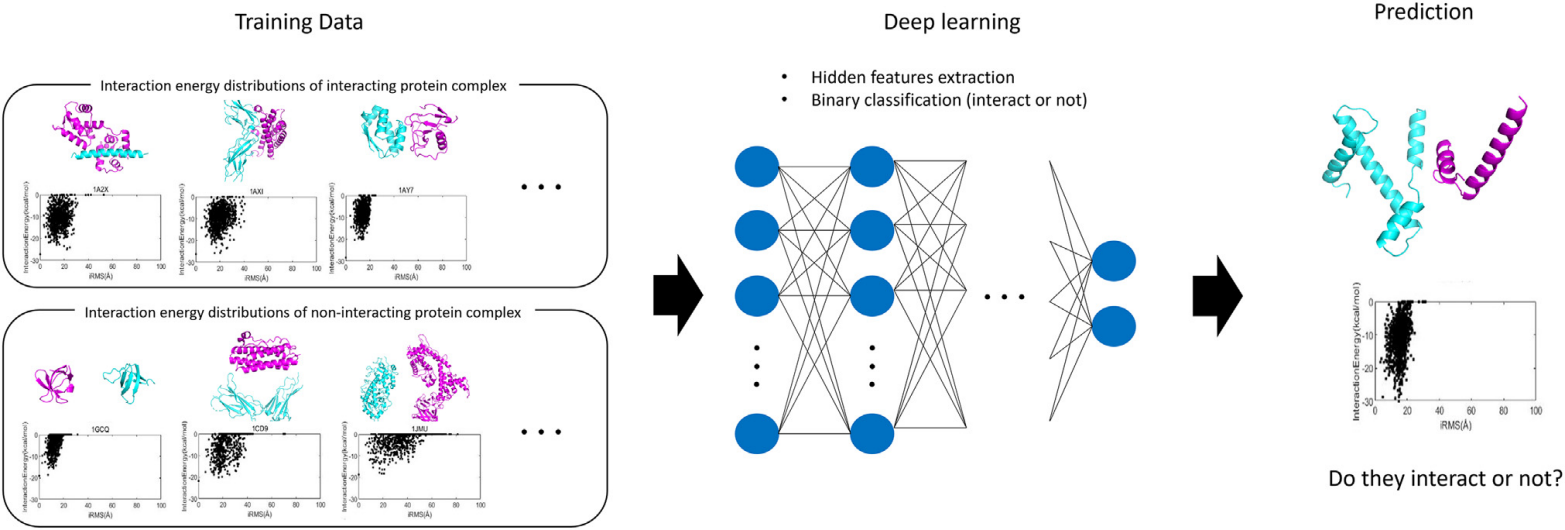
Schematic diagram of protein interaction prediction using interaction energy distributions and deep learning This diagram describes the protein interaction prediction strategy based on the interaction energy diagram.

As I have heretofore demonstrated, there are significant differences in the interaction energy distribution between interacting and non-interacting protein complexes. Thus, I developed deep learning models to predict protein interactions based on complex interaction energy distributions. As described in Figure 5, the prediction program trained with interaction energy distributions of interacting and non-interacting protein pairs uses deep learning to extract hidden distinguishable features from complex distribution patterns and classify them. Then, based on this interaction energy distribution, the prediction program predicts whether the given protein pair will interact.

**Figure 6:**
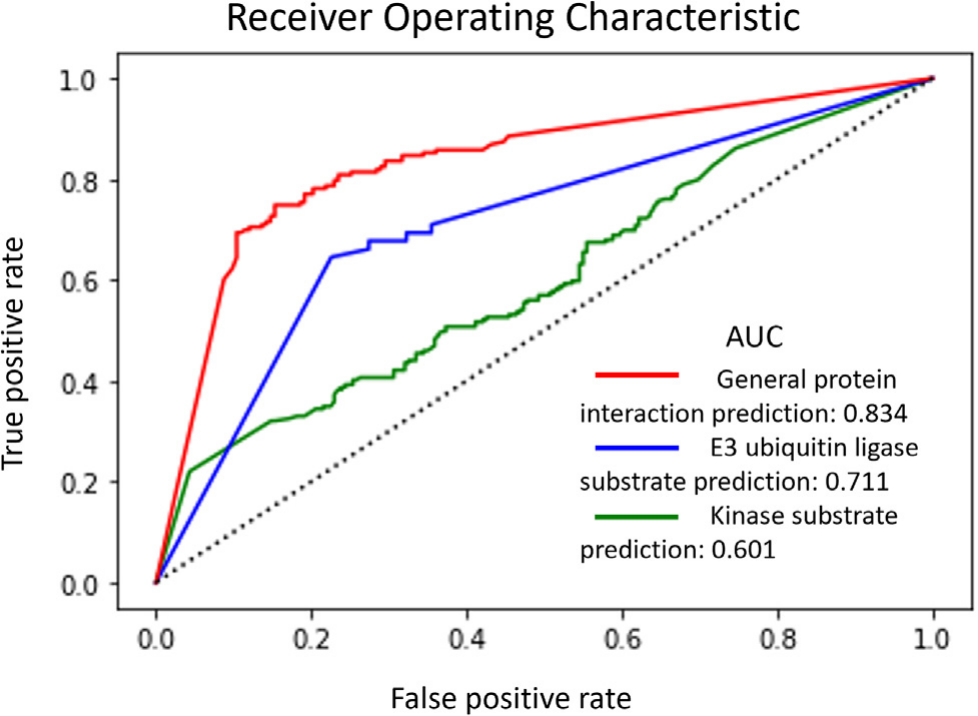
Predictive performance of deep learning models Receiver operating characteristic (ROC) curves from 10-fold cross validation when shuffling data in the deep learning model to prediction protein interactions between E3 ubiquitin ligase (EUL) and substrate, and between kinase and substrate. Each area under curve (AUC) value is notated in the chart.

To develop the prediction model, protein interaction energy distributions of 183 experimentally determined interacting protein complex from the IntAct database^4^ and 110 non-interacting protein pairs from the Negatome 2.0 database^50^ were used for training (Data S5). As the protein interaction energy distribution analysis uses simple two-dimensional numerical data, the deep learning model was designed with a simple recurrent neural network consisting of one block. As interaction prediction can be simplified to binary classification problem, binary cross entropy^53^ was used for loss function. Additionally, to find optimal model structure, I tested seven optimizers from the Keras library, including Adam, SGD, RMSprop, Adadelta, Adamax, Nadam, and Ftrl.^54^ Moreover, considering the amount of input data and the findings of previous studies,^55^^,^^56^ I constructed seven candidate layer structures and tested each. Each of the 49 deep learning models was analyzed with 1–200 epochs. The optimal model for prediction showed an accuracy of 77.6% by utilizing the Adam optimizer containing a 1024-64-2-layer structure and 109 epochs (Figure S13). To assess the predictive performance of this deep learning model, the receiver operating characteristic (ROC) curve from 10-fold cross validation with data shuffling was analyzed. The area under curve (AUC) of this model was 0.834 (Figure 6), demonstrating a higher predictive performance than that of yeast two-hybrid screening (Figure 3C).

To apply this deep learning method to predict substrates of EULs and kinases, I retrieved EULs and kinases from various species along with information about their substrates from the Ubibrowser 2.0^25^ and PhosphoSitePlus^57^ databases. Then, I retrieved predicted structures using AlphaFold2.0 in the Alpha-Fold database^58^ with less than 700 residues and more than 70% prediction confidence in at least half the region. In total, 63 EULs and their substrates and 214 kinases and their substrates were randomly selected and retrieved from Ubibrowser 2.0^25^ and PhosphoSitePlus^57^ because docking programs are too computationally expensive to calculate all data in databases (Data S7). However, to avoid bias, various type of E3 ubiquitin ligases including RING, HECT, Cullin based EUL complex from eight species including animals, plants, bacteria, and fungi were chosen. Moreover, various type of kinase with different functions with 27 unique functional domains and various phosphorylation site from seven species were selected. I then randomly paired enzymes and substrates as a negative control. Among these, pairs with evidence of interactions in the IntAct^4^ or STRING databases^6^ were filtered out. A total of 63 EUL-random substrate pairs and 203 kinase-random substrate pairs were constructed. Their interaction energy distribution was then analyzed using RosettaDock^41^ and HDOCKlite,^42^ as shown in Figure 1. To construct an optimal deep learning model for interaction prediction, 49 candidate models with 7 optimizers in the Keras library and 7 candidate layer structures were analyzed with 1–100 epochs for EULs and 1–50 epochs for kinases (Figures S14–S16). The deep learning model trained using the RosettaDock data (Figures S14 and S15) performed better than that trained using the HDOCKlite data (Figure S16). Therefore, only the deep learning models trained using the RosettaDock data were further analyzed.

For substrate prediction of EULs, the optimal prediction model showed a 77.7% accuracy using an Adam optimizer containing a 1024-2-layer structure and 61 epochs. For substrate prediction of kinases, the optimal model for prediction showed a 61.4% of accuracy with an Adam optimizer containing a 1024-64-2-layer structure and 9 epochs (Figures S14 and S15). ROC curves obtained from 10-fold cross validation with data shuffling were then analyzed to test the predictive performance of these deep learning models. The AUCs of these models were 0.711 and 0.601 for EULs and kinases, respectively. (Figure 6) This result demonstrates higher and similar predictive performance than that of yeast two-hybrid screening, respectively (Figures 3C and 6).

### Protein interaction energy distribution analysis and interaction partner prediction using template modeling score

iRMS is a very useful indicator for analyzing protein interaction positions. However, as the positional information necessary for protein interaction analysis (3D translational shift, rotation, and structural changes of the proteins in the complex) is condensed into a single indicator, this approach has several limitations. Therefore, it is necessary to introduce various indicators. Especially, structural analysis using root-mean-square deviation, including iRMS, is sensitive to local error.^59^ Large local deviation in few residues can results in a large iRMS. Template modeling score (TM-score) is another good indicator for protein structural analysis. In TM-score formula, distance between residues is located in the denominator. Therefore, large local deviation in few residues makes a small change in TM-score. Naturally, TM-score is less sensitive to local error^59^ (Table 2). However, TM-score itself is not appropriate to analyze protein interaction distribution. In the case of a protein pair with large difference in size, the TM-score of the protein pair can almost be determined by the larger protein alone. Instead, three modifications from the TM-score were used for protein interaction analysis.

**Table 2:** Formula and explanation of TM-score and modifications from TM-score

	Formula	Description	Reference
TM-score	$\max\left[\frac{1}{L_{target}}\sum_{i=1}^{L_{aligned}}\frac{1}{1+{\left(\frac{d_{i}}{d_{0}\left(L_{target}\right)}\right)}^{2}}\right]$$L_{target}:the\;amino\;acid\;sequence\;length\;of\;the\;docking\;structure$ $L_{aligned}:the\;length\;of\;the\;aligned\;residues\;to\;the\;strongest\;interacting\;structure$$d_{0}\left(L_{target}\right):the\;distance\;scale\;that\;normalizes\;distance\;to\;make\;TM-score\;length\;independent$ $\left(d_{0}\left(L_{target}\right)≝1.24\sqrt[{3}]{L_{target}-15}-1.8\right)$ $d_{i}:distance\;between\;ith\;residue\;in\;docking\;structure\;and\;strongest\;interacting\;structure$	The TM-score is a length independent measure of similarity between two protein structures. TM-score is between (0,1) and higher is better. (1 means perfect match) Compared to RMSD, it is relatively insensitive to local error.	Zhang et al.^59^
rTM-score	$\frac{N_{protein}}{\sum_{i=1}^{N_{protein}}\left(\frac{1}{{TM-score}_{i}}\right)}$ $N_{protein}:the\;number\;of\;protein\;in\;interacting\;protein\;complex\;or\;non-interacting\;protein\;pair$ ${TM-score}_{i}=TM-score\;of\;i\;th\;protein\;in\;interacting\;protein\;complex\;or\;non-interacting\;protein\;pair$	The rTM-score is the harmonic average of the TM-scores of the components of the protein complex. Therefore, TM-score of one protein cannot dominate rTM-score of protein complex. rTM-score can be used to analyze protein interaction energy distribution with protein docking program with structure refinement.	Zhou et al.,^60^ Derived from TM-score
iTM-score	$\max\left[\frac{1}{L_{interface}}\sum_{i=1}^{L_{aligned\;itf}}\frac{1}{1+{\left(\frac{d_{i}}{d_{0}\left(L_{interface}\right)}\right)}^{2}}\right]$ Interfaceisdefinedaseveryresiduepairsfromtwoproteinswithin10Åofeachother. $L_{interface}:the\;amino\;acid\;sequence\;length\;of\;interface$ $L_{aligned\;itf}:the\;lengt\;of\;the\;aligned\;residues\;in\;interface\;to\;the\;strongest\;interacting\;protein\;complex\;interface$	The iTM-score is a TM-score calculated by restricting the computation to the interfaces of protein complexes Factors unrelated to the interacting position, such as protein size and shape, are ignored or minimized in calculation of iTM-score.	Derived from TM-score
riTM-score	$\frac{N_{protein}}{\sum_{i=1}^{N_{protein}}\left(\frac{1}{{iTM-score}_{i}}\right)}$${iTM-score}_{i}:iTM-score\;of\;i\;th\;protein\;in\;interacting\;protein\;complex\;or\;non-interacting\;protein\;pair$	riTM-score is the harmonic average of the iTM-scores of the components of the protein complex. Therefore, iTM-score of one protein interface cannot dominate riTM-score of protein complex.	Derived from iTM-score and rTM-score

Reciprocal TM-score (rTM-score) is the harmonic average of the TM-scores of the components of the protein complex. Therefore, TM-score of one protein cannot dominate rTM-score of protein complex^60^ (Table 2). In this study, rTM-score of each docking structure was calculated using the most strongly interacting protein complex as a template. However, rTM-score cannot directly measure protein interaction position. The rTM-score calculates the structural similarity between each protein in the docking structure and the most strongly interacting protein complex. Using a docking program with structure refinement, docking structures that have interaction position similar to the template are expected to have a high rTM-score. Therefore, rTM-score can only be used to analyze protein interaction energy distribution with protein docking program with structure refinement.

Moreover, TM-score has shape dependence when used for protein interaction analysis. A slight change in interaction angle can lead to large structural changes depending on the shape. In the case of a long protein, when it interacts to a position similar to the template, it has a smaller TM-score than when a round protein interacts. Therefore, I introduced interface TM-score (iTM-score, Table 2) to overcome size dependence of TM-score for each protein in protein complex. iTM-score is a TM-score calculated by restricting the calculation to interface region rather than entire protein. Interface region defined as residues closely located within 10 Å, which is same as used in iRMS. By ignoring residues not related with interaction, iTM-score minimize shape Furthermore, to overcome size dependence of iTM-score for protein complex with different interface size, I introduced reciprocal iTM-score (riTM-score, Table 2). riTM-score is the harmonic average of the iTM-scores of the components of the protein complex.

**Figure 7:**
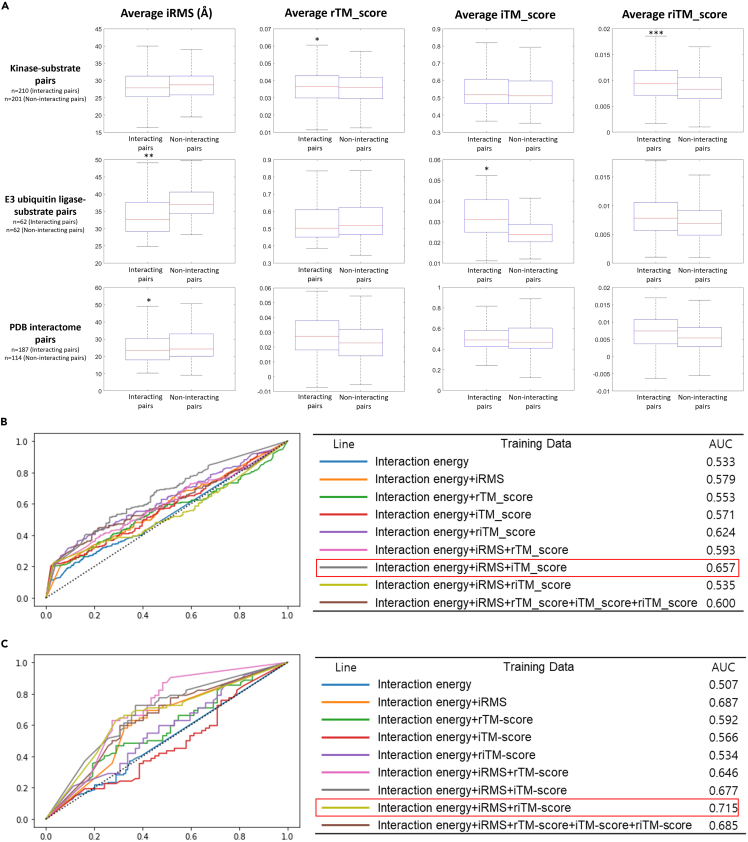
Protein interaction energy distribution analysis using modifications from TM-score (A) Distribution of average iRMSs, rTM-score, iTM-score, and riTM-score of 1,000 docking structures of interacting protein complexes and non-interacting protein pairs including enzyme-random substrate pairs are shown using box-and-whisker plot. Significant differences of average scores with 95% confidence using Student’s *t* test are marked with asterisks, 99% confidence marked with double asterisks and 99.95% confidence marked with triple asterisks. (B) ROC curves from 10-fold cross validation when shuffling data in the deep learning model to prediction protein interactions between kinase and substrate. Each line represents the ROC curve of a model trained with different training data, and the table provides the corresponding area AUC values. The highest AUC value is highlighted with a red box. (C) ROC curves from 10-fold cross validation when shuffling data in the deep learning model to prediction protein interactions between EUL and substrate. Each line represents the ROC curve of a model trained with different training data, and the table provides the corresponding area AUC values. The highest AUC value is highlighted with a red box.

To analyze the distribution of protein substrate interaction energy using modifications from the TM-score, I calculated the iRMS, rTM-score, iTM-score, and riTM-score of docking structures of EUL-substrate or random substrate, as well as those of kinase-substrate or random substrate as mentioned previously (Data S10). Then, I compared average of the iRMS, rTM-score, iTM-score, and riTM-score of between docking structure of these enzyme-substrate complexes and enzyme-random substrate complexes. (Figure 7A) Un-like iRMS, these modifications from TM-score are higher for similar interaction position. Except for rTM-score of kinase-substrate pair, every modifications from TM-score are higher for enzyme-substrate pair than enzyme-random substrate pair. Especially, average rTM-score and riTM-score of the docking structure of the kinase were significantly higher with the substrate than with a random substrate, with 95% and 99.95% confidence according to a Student’s *t* test, respectively. However, average iRMS of the docking structure of the kinase were not significantly different with substrate than with a random substrate according to a Student’s *t* test. Moreover, average iRMS and iTM score of the docking structure of the EUL were significantly different with the substrate than with a random substrate, with 99% and 95% confidence according to a Student’s *t* test, respectively.

Furthermore, to analyze the distribution of protein interaction energy using modifications from the TM-score, I calculated the iRMS, rTM-score, iTM-score, and riTM-score of docking structures of interacting protein complex from the IntAct database^4^ and non-interacting protein pair from the Negatome 2.0 database^50^ as mentioned previously (Data S11). Then, I compared the average of the iRMS, rTM-score, iTM-score, and riTM-score of between docking structure of interacting protein complex and non-interacting protein pair (Figure 7A). Average iRMS of the docking structure of the interacting protein complex were significantly smaller than the non-interacting protein pair, with 95% confidence according to a Student’s *t* test. This means that in order to predict protein interaction partners, it is necessary to compare different indicators according to the nature of each protein interaction.

Modifications from TM-score as well as iRMS are different between interacting protein complex and non-interacting protein pairs. Therefore, I developed deep learning model to predict protein interaction partner using various indicators. As mentioned previously, the deep learning model was designed with a simple recurrent neural network consisting of one block. Then, interaction energy, iRMS, rTM-score, iTM-score, and riTM-score were used for training. I tested eight optimizers from the Keras library, including Adam, SGD, RMSprop, Adadelta, Adamax, Adagrad, Nadam, and Ftrl,^54^ and seven candidate layer structures. Accuracy of each model was analyzed with 1–200 epochs in increments of 10 (Figures S17–S19). Then, among the five models with high accuracy, the ROC analysis was performed using the model with the highest AUC value in the ROC curve analysis. For kinase-substrate interaction, the optimal model for prediction was recurrent neural network model with an Adadelta optimizer containing a 1024-128-2 layer structure and 20 epochs. For EUL-substrate interaction, the optimal model for prediction was recurrent neural network model with an Adagrad optimizer containing a 1024-2-layer structure and 190 epochs. Using these models, ROC analysis was performed for various set of training data (Figure 7B). As many tools that have previously tried to find substrates through interaction energy have shown low accuracy, kinase and EUL had AUC values similar to those of random selection when trained using only interaction energy. For kinase, model trained with interaction energy, iRMS and iTM-score showed AUC value (0.657) in the ROC curve analysis, which is higher than those of optimal model trained with interaction energy and iRMS (Figures 6 and 7B). For EUL, model trained with interaction energy, iRMS and riTM-score showed AUC value (0.715) in the ROC curve analysis, which is similar with those of optimal model trained with iRMS (Figures 6 and 7B). For general protein interaction, the optimal model for prediction was the recurrent neural network model with an Adagrad optimizer containing a 2048-128-2 layer structure and 140 epochs. Using this model, ROC analysis was performed for various set of training data (Figure S20). However, this model showed AUC value lower than those of optimal model trained with iRMS (Figures 6 and S20).

It was analyzed whether protein interaction partners could be predicted using interaction energy distribution even using predicted structures of general proteins. Predicted protein structure of interacting protein complex from the IntAct database^4^ and non-interacting protein pair from the Negatome 2.0 database^50^ by AlphaFold2 were retrived in the Alpha-Fold database.^58^ Then, 1,000 docking structures per protein pair were generated using RosettaDock and interaction energy, iRMS, rTM-score, iTM-score, and riTM-score were calculated as mentioned previously (Data S11). Because protein interaction energy distribution using predicted protein structure is affected by protein length and prediction accuracy, filtered dataset with less than 700 amino acids length proteins and more than 70% region with high confidence (pLDDT>90)^58^ was generated and analyzed. Average rTM-score and riTM-score of the docking structure of the interacting protein complex were slightly higher than non-interacting protein pair (Figure S21A). Even though differences in filtered dataset were bigger than unfiltered dataset, differences were not significantly different with 95% of confidence according to the Student’s *t* test. Deep learning model to predict protein interaction partner using filtered dataset was developed as mentioned previously. The optimal model was the recurrent neural network model with an Adadelta optimizer containing a 2048-512-128-32-8-2 layer structure and 40 epochs. This model trained with interaction energy, riTM-score showed AUC value (0.605) in the ROC curve analysis (Figure S21B).

## Discussion

In this study, I demonstrated that the interaction of a protein with a specific interaction partner has a narrow, funnel-like interaction energy distribution. In addition, interaction energy distributions between interacting and non-interacting protein pairs are substantially different. Especially, PPIs related with various signaling pathway showed much narrower interaction energy distribution than those of random pair. Although these PPIs, including PPIs of kinases or EULs, are difficult to predict and validate using experiments and previous prediction methods, the interaction energy distributions had considerably distinguishable traits. Moreover, because iRMS is too sensitive to local error, I introduced rTM-score^60^ and two new protein interaction position indicators derived from TM-score, iTM-score, and riTM-score, to analyze protein interaction energy distribution (Table 2). These protein interaction position indicating scores, including iRMS and scores derived from TM-score, showed different distributions according to the interaction type. Especially, for kinase, iRMS did not show significant difference between kinase-substrate pair and kinase-random substrate pair. However, riTM-score showed much significant difference between kinase-substrate pair and kinase-random substrate pair with 99.95% of confidence according to Student’s *t* test. This shows that various indicators should be used for each protein interaction analysis.

I then developed deep learning models to predict interaction partner based on the interaction energy distribution. The deep learning model trained using RosettaDock data performed better than that trained using HDOCKlite data, indicating that a more accurate interaction energy calculation with structure refinement is necessary to accurately predict protein interactions. Moreover, as weak as kinase-substrate and EUL-substrate interactions are, the model trained on only interaction energies was close to random selection (Figure 7B, AUC ∼0.5). However, by training interaction energy distribution with iRMS, predictions were much improved. Furthermore, general protein interaction prediction also improved by training interaction energy distribution with iRMS. In case of kinase and EUL substrate prediction, substrate prediction can be further improved by training with iTM-score and riTM-score, respectively. This synergetic effect is remarkable. For kinase substrate prediction, riTM-score showed most significant difference between kinase-substrate pair and kinase-random substrate pair and model trained with riTM-score was best among model trained with single positional indicator. However, including model trained with multiple indicators, model trained with iRMS and iTM-score showed better performance. ROC curve analysis showed that general protein interaction prediction and EUL substrate prediction had a good predictive performance, with an AUC of 0.7–0.9. However, kinase substrate prediction and general protein interaction partner prediction using predicted structure only had an acceptable predictive performance, with an AUC of approximately 0.6–0.7. It may be related with dynamic and diversity of interaction. Both predictions showed better performance with scores modified from TM-score than iRMS, which may be related with local error. Protein docking program with structure refinement have limited capacity to refine protein structure during protein interaction. In case of EUL, substrate interacts with E2 enzyme as well as EUL or more. Therefore, EUL-substrate interaction will be more stationary in position than kinase-substrate interaction. Moreover, protein structure from PDB data includes structural change during the interaction. However, AlphaFold2 predicts stable structure on its own. Therefore, limited consideration of structural changes during the interaction may hamper prediction. This problem can be improved by introducing new indicators, improving docking programs and predicting protein structure in the context of protein interaction. Currently, most protein interaction prediction methods are biological evidence- and known protein complex structure template-based.^5^^,^^6^^,^^7^^,^^8^^,^^9^^,^^10^^,^^11^^,^^14^^,^^17^ However, these methods that require omics scale interspecies biological data or known similar interactions display limited predictability. Knowledge-free protein interaction prediction methods would be necessary to describe the interactome in various species. Therefore, I focused on a protein docking program, which simulates the physical interactions of proteins. Protein docking programs have been developed for over 30 years.^61^ With communitywide docking program assessment, Critical Assessment of PRediction of Interactions (CAPRI),^62^ recent docking programs are showing good performance in predicting protein complex structures.^63^ However, docking programs are rarely used to find interacting partners for two reasons: low accuracy in weak and transient interactions and computational costs.^5^^,^^15^^,^^61^

In 2011, using supercomputers, protein docking structures of interacting and non-interacting protein pairs were generated for up to 100,000 structures per each pair and compared.^15^ Although over half of the protein complexes showed a better docking score than 85% of the background, the approach showed lower performance for enzyme-inhibitor interactions. Moreover, most protease interactions were similar to those of the background.^15^ Three reasons explain why docking score, consisting of interaction energy and the shape complementarity of the docking structure, was not enough for interacting partner prediction of high accuracy. First, investigation of proteomic quantity is missing. Even an interaction partner with weak interaction energy can be a dominant interaction partner if its cellular amount is large.^19^ However, most cellular quantities of proteins are unknown. Second, all protein energy functions exhibit a small difference from reality. Even though multiple protein energy functions were developed, energy-based free protein structure modeling showed poorer performance than template-based modeling.^64^ Moreover, the Rosetta energy function, one of the successful and widely used energy functions, predicts that the ΔΔG of the HIV1 protease T193V mutation is −4.95 kcal/mol, but the experimental result was −1.11 kcal/mol.^35^^,^^65^ In contrast, due to the competition effect, a slightly higher interaction energy is enough to be a dominant interaction partner.^31^ Therefore, minor errors in the energy function can hinder the interaction partner prediction. Third, low shape complementarity of transient interaction may not be distinguishable from those of others.^5^^,^^18^ Certain docking scores use protein complex geometric shape complementarity,^42^^,^^66^ but transient interaction partners do not have dominant shape complementarity, distinguishable from those of others.

In this study, I used specificity, as well as interaction energy, to distinguish protein interaction partners. Specificity is the result of a long evolution.^67^ Because of specificity, complicated molecular cascades in biological processes and complex life activities can be maintained.^31^ As specificity in multiple PPIs depends on the interaction in specific regions, such as van der Waals and electrostatic interactions,^31^^,^^67^ such specific PPIs are in a specific orientation. For example, p53, a well-known tumor suppressor, has more than 20 and 10 phosphorylation and ubiquitination sites, respectively. Each site is phosphorylated and ubiquitinated by specific kinases and EULs.^68^ These PPIs in specific orientation result in narrow funnel-like interaction energy distribution (Figure 1A). In this study, I showed that specific interaction partner search with narrow funnel-like interaction energy distribution achieved similar accuracy as yeast two-hybrid screening (Figures 3C, 6, and 7). Therefore, the narrow funnel-like interaction energy distribution is a key indicator of specific interaction partners.

High computational cost is another hurdle to using docking programs for interaction partner predictions. In this study, PPI prediction with narrow funnel interaction energy distribution using data from HDOCKlite, an FFT-based rigid body docking program,^42^ showed good performance in specific interaction partner distinction (Figure 3C). As this program runs fast even on personal computers (Table 1), it can predict specific interaction partners without additional computational power. Moreover, protein interaction prediction on the proteomic level might be feasible with small computational power. RosettaDock includes docking structure refinement using the Monte-Carlo algorithm.^41^ Therefore, it can calculate cooperative interactions, accompanied by structure changes.^69^ Furthermore, I showed that 1,000 docking structures per each pair are enough for interaction partner prediction. Therefore, the narrow funnel-like interaction energy distribution-based approach adapted to small computational power. Due to the small computation amount, interaction predictions on the proteome scale are also possible.

However, this study also has clear limitations. First, protein structures should be predicted or determined and the structure prediction result affects PPI prediction results (Figures 3, 7, S9, S10, S20, and S21). However, amazing progress has been recently achieved in protein structure prediction. Protein structure prediction tools using deep learning were developed.^70^^,^^71^ In a recent blind protein structure prediction assessment, critical assessment of protein structure prediction 14,^64^ Alpha-predicted certain targets with higher accuracy than template-based predictions.^72^ Protein structure prediction error is decreasing close to the error of protein structure determination using X-ray crystallography.^71^^,^^72^ Moreover, predicted structures are being accumulated in databases and such structures are being published.^58^ Therefore, proteome-scale predicted protein structures can be used by anyone in the near future.

Second, protein size affects the prediction results (Figures 3, S9, and S10). As global docking works better for small than large proteins,^48^ interaction energy distribution is affected by protein size. This bias can be improved through high resolution *in silico* protein docking with more docking structures.^15^ Furthermore, interaction partner prediction with interaction energy distribution can be used in parallel with other interaction partner predictions. Widely used biological result- and template structure-based protein interaction prediction methods are biased by well-studied interaction pairs.^5^ However, the interaction energy distribution is completely irrelevant for the extent of how well-studied these interactions are. This method is biased by protein pairs with well-predicted structures and of small size (Figures 3, S9, and S10). They can be used together to complement each other.

To improve this study further, improvement of protein energy function should be considered. There are two kinds of protein energy functions. One is the physics-based energy function, constructed based on classical mechanics and correction terms with perturbations in quantum mechanics, such as the Chemistry at Harvard Macromolecular Mechanics (CHARMM) force field.^73^ The other is knowledge-based energy function, using Boltzmann relation to retrieve energy terms from experimentally determined protein structures, such as Rosetta energy.^35^ These energy functions are developed using a bottom-up approach consisting of each formula and parameter of physical properties like building blocks.^35^^,^^73^ Terms might also be missing or contain big errors. Therefore, from a holistic point of view, protein energy might be retrieved using the Boltzmann relation from protein structures. To do so, significant amounts of structures are necessary, although the experimentally identified protein structures are limited. However, due to the recent progress in the protein structure prediction-related deep learning field, we can predict distribution of protein structures. In Alpha-Fold, predicted distance and torsion distribution are generated by deep learning and the potentials are retrieved with predicted distributions using Boltzmann relation without specific physical energy formula.^70^ Therefore, interaction energy can be generated with a top-down approach using deep learning. Moreover, the Boltzmann distribution is valid in the equilibrium state.^74^ However, living organisms are dynamic, open, and are in a nonequilibrium state. To describe life phenomena more precisely, nonequilibrium statistical mechanics, such as generalized Boltzmann distribution, would be necessary.^75^^,^^76^

In this study, the development of EUL substrate prediction using deep learning enabled the proposal of new application. Innovative methods such as CRISPR/Cas9, siRNA, or miRNA, which inhibit DNA and RNA levels in the central dogma, have limitations as potential medicines for regulating the expression of a specific gene. Therefore, recently, studies on degrading undrugable targets at the protein level using small molecules called proteolysis targeting chimera (PROTAC) that help interaction to EUL have been actively conducted.^77^^,^^78^ More than 3,000 PROTACs have been developed or reported, and dozens of these have entered clinical trials. In fact, ARV-110 and ARV-471 have both entered phase II clinical trials.^78^^,^^79^ However, due to the limited understanding of EUL-substrate interactions, studies on PROTACs have been restricted to a few EULs, especially cereblon (CRBN). To broaden target range and reduce side effects, more various EULs should be used with PROTACs.^77^^,^^78^^,^^79^ It is possible to investigate the available EUL by analyzing the interaction energy distribution between target-PROTAC complex and the EUL. The structure of a protein target-PROTAC complex can be experimentally determined using cryo-EM,^80^ X-ray crystallography,^81^ or other methods, or predicted using a well-developed protein-ligand docking program.^82^^,^^83^ In addition, the off-target effect of PROTAC can be calculated using this analysis.

Another possible interaction energy distribution analysis application is that narrow funnel-like interaction energy distribution can be a specificity indicator. Finding specific interactions is important in drug discovery. Nonspecific interactions can result in side effects.^84^ Even though narrow funnel-like interaction energy distribution does not guarantee the exclusion of binding other molecules, it shows that proteins bind to a specific site and can be used as a specific interaction indicator. In particular, several kinases and EULs are considered as targets of multiple drugs, such as anticancer drugs.^85^^,^^86^ Therefore, this study could be applied in drug discovery as a specific interaction indicator.

### Limitations of the study

Still, this study has several limitations. Although over 1,000 PPIs were analyzed in this study, this number is far less than the number of discovered PPIs. As demonstrated in this study, different types of protein interactions exhibit distinct patterns of interaction energy distribution. Therefore, limited protein interaction analysis leads to restricted data on the distribution patterns of interaction energy. Especially, Artificial Intelligence (AI) model for predicting PPIs trained on limited data can exhibit low accuracy for diverse proteins. Therefore, further research involving large-scale analysis utilizing effective protein docking program and high-performance computing device such as supercomputer will improve PPI prediction.

Moreover, indicators for PPI used in this study (iRMS, rTM-score, iTM-score, and riTM-score) require strongest interacting docking structure as a model structure. Therefore, these indicators may be not appropriate for analyzing PPIs with multi-binding sites or allosteric regulation. Furthermore, because of small dataset and curse of dimensionality,^87^^,^^88^ I have utilized these indicators to analyze PPIs in this study, without directly using spatial information such as the three-dimensional position and rotation of the protein. If a large PPI interaction energy dataset from large scale analysis is available to avoid the curse of dimensionality, it would be necessary to analyze which information is more accurate for analyzing PPIs.

This study only presents an initial model that demonstrates the possibility of predicting protein interactions using the distribution of interaction energy. Deep learning model applied in this study is simple recurrent neural network. Diverse AI structure including convolutional neural network^89^ and transformer^90^ can improve performance of AI model. Furthermore, to improve accessibility for scientists in various fields of protein research, it should be provided as an online server with a good graphical user interface, like STRING,^6^ PRISM,^14^ and prePPI^17^ server.

## STAR★Methods

### Key resources table

**Table:** 

REAGENT or RESOURCE	SOURCE	IDENTIFIER
**Deposited data**		

Protein interactome data	IntAct database^4^ (https://www.ebi.ac.uk/intact/)	RRID:SCR_006944
E3 ubiquitin ligases-substrates data	UbiBrowser 2.0^25^ (http://ubibrowser.bio-it.cn/ubibrowser_v3/)	N/A
Non-interacting protein data	Negatome 2.0 database^50^ (http://mips.helmholtz-muenchen.de/proj/ppi/negatome)	N/A
Kinases-substrates data	PhosphoSitePlus^57^ (https://www.phosphosite.org/)	RRID:SCR_001837
Predicted protein structure using AlphaFold2^72^	Alpha-Fold database^58^ (https://alphafold.ebi.ac.uk/)	N/A

rowhead**Software and algorithms**		

Phyre2: Protein structure prediction server	Phyre2 server^40^ (http://www.sbg.bio.ic.ac.uk/∼phyre2/)	RRID:SCR_010270
RosettaDock	RosettaCommons^41^ (https://www.rosettacommons.org/)	RRID:SCR_015701
HDOCKlite	HDOCK server^42^ (http://hdock.phys.hust.edu.cn/)	N/A
DockQ	Sankar et al.(2016)^43^ (https://github.com/bjornwallner/DockQ/)	N/A
Deep learning model for protein interaction using interaction energy distribution	This paper (https://github.com/cjy318/protein_interaction)	N/A

### Resource availability

#### Lead contact

Further information and requests for resources should be directed to and will be fulfilled by the lead contact, Juyoung Choi (rgdfs@sogang.ac.kr)

#### Materials availability

This study did not generated or used new unique materials.

### Experimental model and subject details

This study did not include experiment with specific model or subject.

### Method details

#### Protein structure preparation

To analyze kinase interactions, 135 kinase-interaction partner pairs were retrieved from kinase and protein interaction databases.^3^^,^^38^ Using NumPy’s random number generator,^91^ 106 random, not identified as interacting pairs in literature and datasets, were generated. (Data S1) From the E3 ubiquitin ligase-substrate interaction database UbiBrowser,^25^ 189 E3 ubiquitin ligase-substrate interactions were retrieved. Using NumPy’s random number generator,^91^ 124 random, not identified as interacting pairs in literature and datasets, were generated. (Data S1) Using the Phyre2 server,^40^ protein structures were predicted with the intensive mode option. (Data S2)

To analyze interacting protein complexes, 183 protein complex structures were retrieved from the protein interactome database IntAct.^4^ Interacting chains in the protein complexes were analyzed using the interfaceResidue script of PyMOL.^92^ As negative controls, 110 experimentally determined protein structures, unlikely engaged in direct interactions, were retrieved from the Negatome 2.0 database.^50^

To analyze interacting protein with predicted protein structure using AlphaFold2,^71^ predicted protein structures are retrieved from Alpha-Fold database.^58^ Compared to interaction energy distribution analysis using PDB data, proteins not updated in the Alpha-Fold database^58^ were excluded and more non-interacting protein pairs were added from Negatome 2.0 database^50^ to avoid imbalance of size between filtered datasets. (Data S11)

#### Protein docking structure generation using RosettaDock

For RosettaDock, I used the Rosetta 2020.08 bundle.^41^ Each kinase and E3 ubiquitin ligase protein interaction pairs were merged using PyMOL.^92^ Kinase or E3 ubiquitin ligase chain IDs were designated as “A” and those of interacting partners as “B” using PyMOL.^92^ Before kinase and E3 ubiquitin ligase protein pair docking using RosettaDock,^41^ I optimized their side-chain conformations (prepacking) using the following command:

./bin/docking_protocol.static.linuxgccrelease -in:file:s (Merged protein structure file) -docking:partners A_B -dock_pert 3 8 -randomize1 -randomize2 -spin -out:path:all (Output directory)

Next, I generated 1,000 docking structures using the following command.

./bin/docking_protocol.static.linuxgccrelease -in:file:s (prepacked files) -docking:partners A_B -dock_pert 3 8 -randomize1 -randomize2 -spin -use_ellipsoidal_randomization true -nstruct 1000 -out:path:all (output directory)

To analyze interacting proteins complexes and non-interacting protein pairs, I optimized their side-chain conformations (prepacking) using the following command.

./bin/docking_protocol.static.linuxgccrelease -in:file:s (protein complex structure file) -docking:partners (chain ID of proteins which participating in interaction)_(chain ID of proteins in opposite side of interaction) -dock_pert 3 8 -randomize1 -randomize2 -spin -out:path:all (output directory)

Then, I generated 1,000 docking structures using the following command.

./bin/docking_protocol.static.linuxgccrelease -in:file:s (protein complex structure file) -docking:partners (chain ID of proteins which participating in interaction)_(chain ID of proteins in opposite side of interaction) -dock_pert 3 8 -randomize1 -randomize2 -spin -use_ellipsoidal_randomization true -nstruct 1000 -out:path:all (output directory)

To analyze kinase and E3 ubiquitin ligase interactions using HDOCKlite,^42^ I generated 1,000 docking structures using the following command.

./hdock (kinase or E3 ubiquitin ligase) (Interaction partner) -out (docking file)

./createpl (docking file) top1000.pdb -nmax 1000 -complex -models

All commands were written in a Linux shell file and executed.

For further analysis, kinase or E3 ubiquitin ligase chain IDs were designated as “A” and those of interacting partners as “B” using PyMOL.^92^ The graphical representations of the docking structure were exported using PyMOL.^92^

#### Interaction energy, iRMS, rTM-score, iTM-score and riTM-score analysis

To calculate interaction energy, I calculated the Rosetta energy of each protein or complex using the following command:

./bin/score_jd2.linuxgccrelease -in:file:s (protein or complex) -out:file:scorefile (score file)

Then, I calculated the interaction energy using the following equation:

InteractionEnergy={Rosettaenergyofsimulateddockingstructure}

$$-\sum_{Each\;component\;in\;pair}\left\{Rosetta\;energy\;of\;single\;components\right\}$$

Then, iRMS were calculated using DockQ^43^ and the following command.

./DockQ.py (Docking structure) (Strongest interacting docking structure) -short ≫>>(iRMS file)

For interacting protein complexes and non-interacting protein pairs, iRMS were calculated using the following command:

./DockQ.py (Docking structure) (Strongest interacting docking structure) -short -native_chain1 (chain ID of proteins which participating in interaction) -model_chain1 (chain ID of proteins which participating in interaction) -native_chain2 (chain ID of proteins in opposite side of interaction) -model_chain2 (chain ID of proteins in opposite side of interaction) -perm1 -perm2 >>(iRMS file)

All commands were written in a Linux shell file and executed.

Moreover, in this study, rTM-score, iTM-score and riTM-score are applied to interaction energy distribution analysis. (Table 2) To calculate these scores, TM-score was calculated as previous study.^93^^,^^94^

Interface in iTM-score and riTM-score is defined as every residue pairs from two proteins within 10 Å of each other. Interface are selected using interface selection module to calculate iRMS in DockQ program.^43^

#### Gene ontology analysis

The bioinformatics analysis was performed as described previously,^95^ with small modifications. Briefly, using OmicsBox v2.0.36, protein sequences were blasted specifically using the NCBI nr database. Functional domains and motifs were distinguished using the EMBL-EBI Inter-Pro database.^96^ Then, OmicsBox was used to annotate these results. Finally, I summarized gene ontology results according to level-2 gene ontology distribution from Generic GO slim^51^ using OmicsBox.

#### Development of deep learning model and validation

To construct training dataset for general PPI prediction using deep learning, iRMS and interaction energy of 183 protein complex structures, retrieved from IntAct database^4^ and 110 non-interacting protein pairs, retrieved from the Negatome 2.0 database^50^ were calculated as above. To construct training dataset for prediction of kinase and EUL substrate, 214 kinase-substrate pair and 63 EUL-substrate pair were retrieved from PhosphoSitePlus^57^ and UbiBrowser 2.0^25^ database, respectively. (Data S7) Predicted protein structure were retrieved from Alpha-Fold database.^58^ Then, 1,000 simulated docking structure were generated using RosettaDock^41^ and HDOCKlite,^41^ as described above. Then, interaction energy and iRMS were calculated as described above.

Deep learning models were designed as a simple recurrent neural network model with one block using Keras library.^54^ All data and codes, used for deep learning, are publicly available on GitHub. (https://github.com/cjy318/protein_interaction).

## Data Availability

• All codes, used in this study, are deposited and publicly available on GitHub. (https://github.com/cjy318/protein_interaction) • Every numerical data in this study is included in Supplementary Data. Training data for deep learning model is deposited and publicly available on GitHub. (https://github.com/cjy318/protein_interaction) • Any additional information required to reanalyze the data in this study is available from the lead contact upon request.

## Supplemental information

Document S1 Figures S1–S21 and Tables S1 and S2

Data S1. Information of pairs of kinases and their interacting partner and pairs of E3 ubiquitin ligases and their substrates retrived from databases; related to Figure 1

Data S2. Protein structure prediction results using Phyre2.0; related to Figure 1

Data S3. Interaction energy and iRMS of 1,000 simulated docking structure of pairs of kinases and their interacting partner and pairs of E3 ubiquitin ligases generated using RosettaDock; related to Figures 1, 3, S1–S4, S9, and S10

Data S4. Interaction energy and iRMS of 1,000 simulated docking structure of pairs of kinases and their interacting partner and pairs of E3 ubiquitin ligases generatedbusing HDOCKlite; related to Figures 1, 3, and S5–S10

Data S5. Information of general protein interactome and non-interacting protein pairs retrieved from databases and their interaction energy distributions; related to Figures 4, S11, and S12

Data S6. Functional annotation of proteins in general protein interactome and non-interacting protein pairs retrieved from databases. (protein structures used in computational docking were retrieved from PDB datrabase); related to Figure 4

Data S7. Functional annotation of proteins in general protein interactome and non-interacting protein pairs retrieved from databases. (protein structures used in computational docking were retrieved from PDB datrabase); related to Figure 4

Data S8. Interaction energy distributions of pairs of kinases, E3 ubiquitin ligases and their substrates retrived from databases for training of deep learning model generated using RosettaDock; related to Figures 5, 6, S14, and S15

Data S9. Interaction energy distributions of pairs of kinases, E3 ubiquitin ligases and their substrates retrived from databases for training of deep learning model generated using HDOCKlite; related to Figures 5, 6, and S16

Data S10 Interaction energy distributions of pairs of kinases, E3 ubiquitin ligases and their substrates analyzed with iRMS, rTM-score, iTM-score, and riTM-score; related to Figures 5, 7, S17, and S18

Data S11. Interaction energy distributions of general protein interactome and non-interacting protein pairs analyzed with iRMS, rTM-score, iTM-score, and riTM-score (protein structures used in computational docking were retrieved from PDB datrabase.); related to Figures 5, 7, S19, and S20

Data S12. Interaction energy distributions of general protein interactome and non-interacting protein pairs analyzed with iRMS, rTM-score, iTM-score, and riTM-score (protein structures used in computational docking were predicted using AlphaFold2.); related to Figure 5, 7, and S21
