# Supplementary material for: Narrow funnel-like interaction energy distribution is an indicator of specific protein interaction partner
Source: iScience. 2023 May 20;26(6):106911. doi: 10.1016/j.isci.2023.106911 (PMC10250834; doi:10.1016/j.isci.2023.106911)
Supplement: Document S1 Figures S1–S21 and Tables S1 and S2 [file mmc1.pdf]

iScience, Volume *26*

Supplemental information

Narrow funnel-like interaction energy distribution is an indicator of specific protein interaction partner

Juyoung Choi

Supplementary Data Information

Supplementary Data 1: Information of pairs of kinases and their interacting partner and pairs of E3 ubiquitin ligases and their substrates retrived from databases; related to Figure 1.

Supplementary Data 2: Protein structure prediction results using Phyre2.0; related to Figure 1.

Supplementary Data 3: Interaction energy and iRMS of 1,000 simulated docking structure of pairs of kinases and their interacting partner and pairs of E3 ubiquitin ligases generated using RosettaDock; related to Figure 1, 3, S1-S4, S9-S10.

Supplementary Data 4: Interaction energy and iRMS of 1,000 simulated docking structure of pairs of kinases and their interacting partner and pairs of E3 ubiquitin ligases generated using HDOCKlite; related to Figure 1, 3, S5-S10.

Supplementary Data 5: Information of general protein interactome and non-interacting protein pairs retrieved from databases and their interaction energy distributions; related to Figure 4, S11-S12.

Supplementary Data 6: Functional annotation of proteins in general protein interactome and non-interacting protein pairs retrieved from databases. (protein structures used in computational docking were retrieved from PDB datrabase); related to Figure 4.

Supplementary Data 7: Information of pairs of kinases, E3 ubiquitin ligases and their substrates retrived from databases for training of deep learning model for substrate prediction; related to Figure 5, 6, S14-S16.

Supplementary Data 8: Interaction energy distributions of pairs of kinases, E3 ubiquitin ligases and their substrates retrived from databases for training of deep learning model generated using RosettaDock; related to Figure 5, 6, S14-S15.

Supplementary Data 9: Interaction energy distributions of pairs of kinases, E3 ubiquitin ligases and their substrates retrived from databases for training of deep learning model generated using HDOCKlite; related to Figure 5, 6, S16.

Supplementary Data 10: Interaction energy distributions of pairs of kinases, E3 ubiquitin ligases and their substrates analyzed with iRMS, rTM-score, iTM-score and riTM-score; related to Figure 5, 7, S17-S18.

Supplementary Data 11: Interaction energy distributions of general protein interactome and non-interacting protein pairs analyzed with iRMS, rTM-score, iTM-score and riTM-score (protein structures used in computational docking were retrieved from PDB datrabase.); related to Figure 5, 7, S19-S20.

Supplementary Data 12: Interaction energy distributions of general protein interactome and non-interacting protein pairs analyzed with iRMS, rTM-score, iTM-score and riTM-score (protein structures used in computational docking were predicted using AlphaFold2.) ; related to Figure 5, 7, S21.

Supplementary Figures and Tables

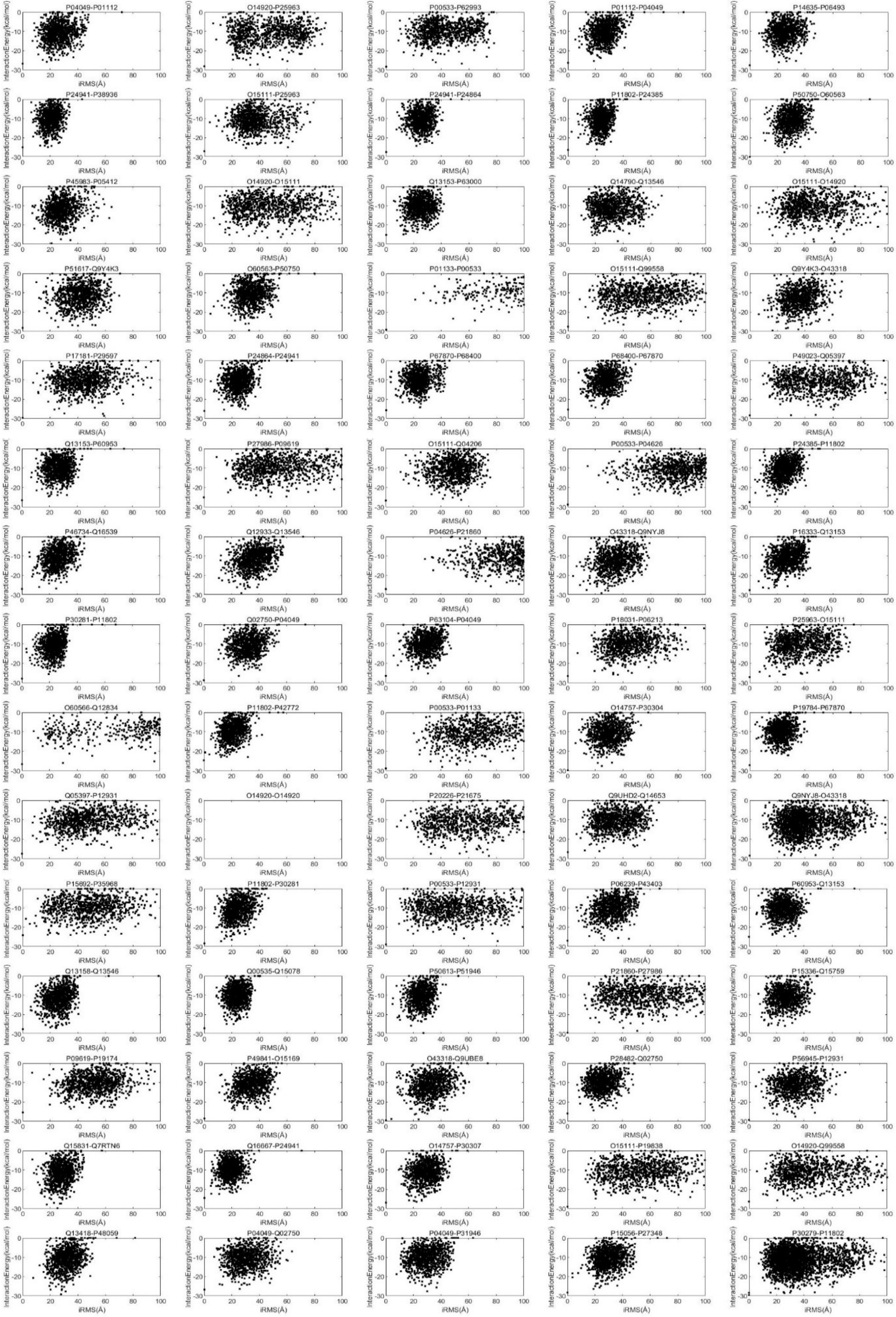

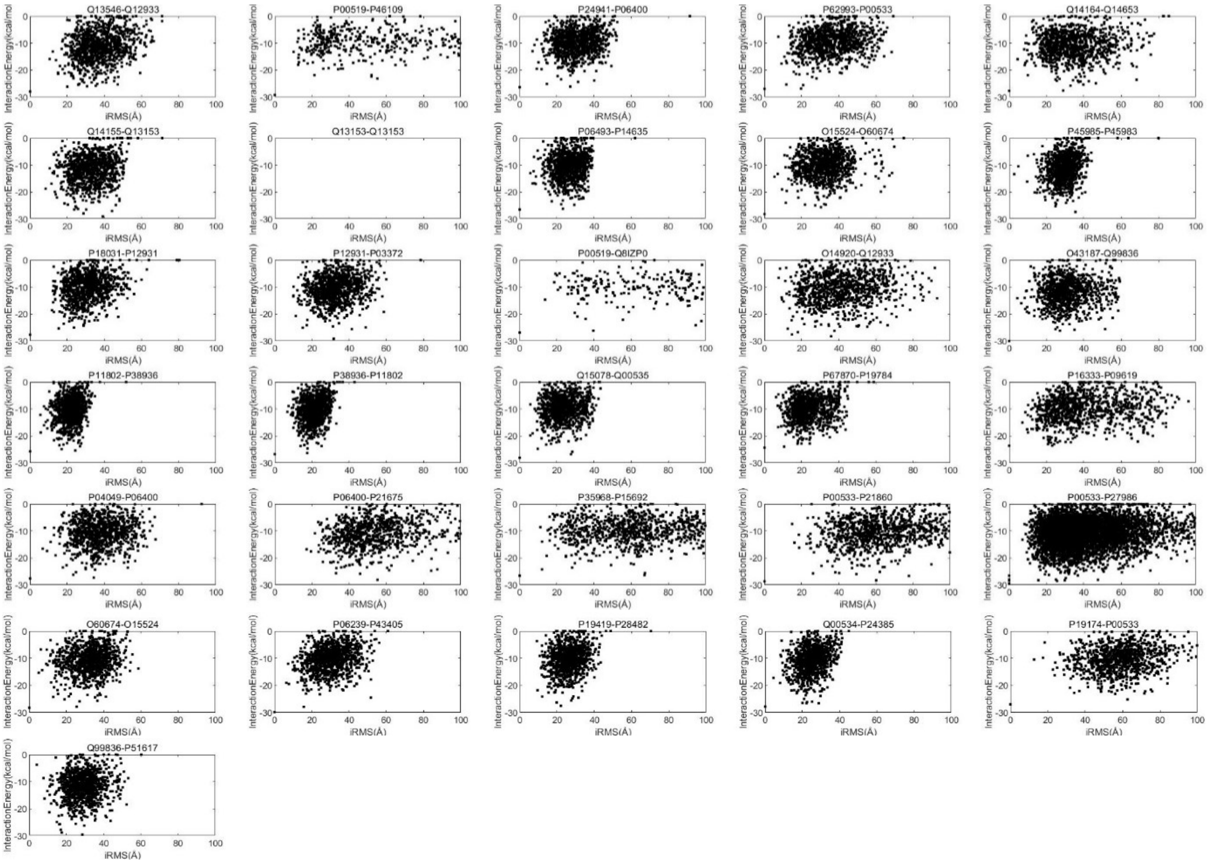

Figure S1: Interaction energy distribution of 131 kinase-specific interaction partner pairs, docking simulated using RosettaDock.

Interaction energy of 1000 simulated docking structures of 131 kinase-specific interaction partner pairs. Protein dockings were simulated with RosettaDock. Each kinase and specific interaction partner uniport ID is indicated above. Interaction energies were calculated with Rosetta energy. iRMS were calculated between each simulated docking structure and the most stable docking structure. To compare distributions, the scale was fixed (iRMS: 0–100Å, Interaction energy: −30 to 0 kcal/mol). Points outside this range were ignored; related to Figure 1 and 3.

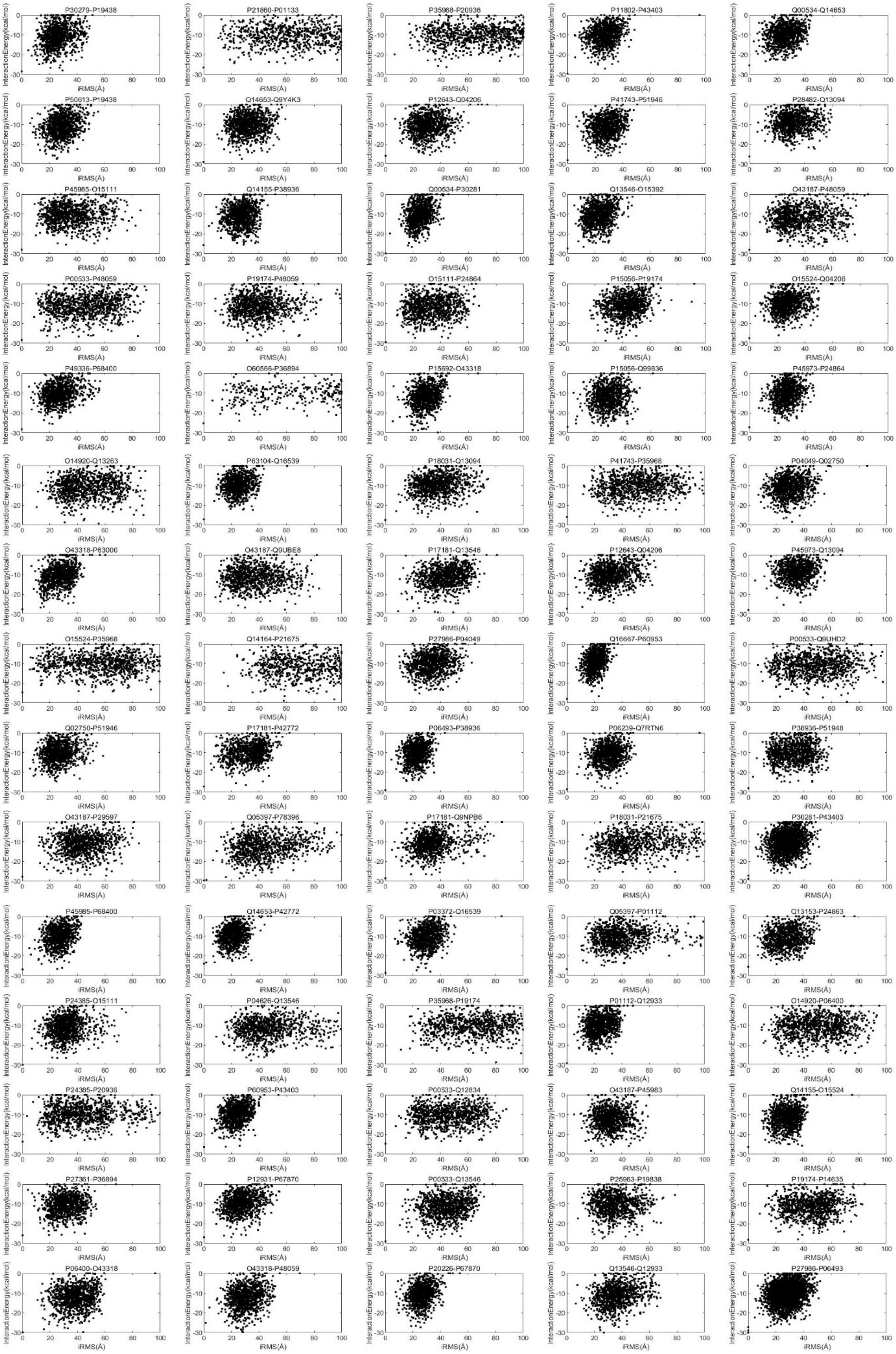
P30279-P19438
P21860-P01133
P35968-P20936
P11802-P43403
Q00534-Q14653
P50613-P19438
Q14653-Q9Y4K3
P12643-Q04206
P41743-P51946
P28482-Q13094
P45985-O15111
Q14155-P38936
Q00534-P30281
Q13546-O15392
O43187-P48059
P00533-P48059
P19174-P48059
O15111-P24864
P15056-P19174
O15524-Q04206
P49336-P68400
O60566-P36894
P15692-O43318
P15056-Q99836
P45973-P24864
O14920-Q13263
P63104-Q16539
P18031-Q13094
P41743-P35968
P04049-Q02750
O43318-P63000
O43187-Q9UBE8
P17181-Q13546
P12643-Q04206
P45973-Q13094
O15524-P35968
Q14164-P21675
P27986-P04049
Q16667-P60953
P00533-Q9UHD2
Q02750-P51946
P17181-P42772
P06493-P38936
P06239-Q7RTN6
P38936-P51948
O43187-P29597
Q05397-P78396
P17181-Q9NPB6
P18031-P21675
P30281-P43403
P45985-P68400
Q14653-P42772
P03372-Q16539
Q05397-P01112
Q13153-P24863
P24385-O15111
P04626-Q13546
P35968-P19174
P01112-Q12933
O14920-P06400
P24385-P20936
P60953-P43403
P00533-Q12834
O43187-P45983
Q14155-O15524
P27361-P36894
P12931-P67870
P00533-Q13546
P25963-P19838
P19174-P14635
P06400-O43318
O43318-P48059
P20226-P67870
Q13546-Q12933
P27986-P06493
InteractionEnergy(kcal/mol)
iRMS(Å)

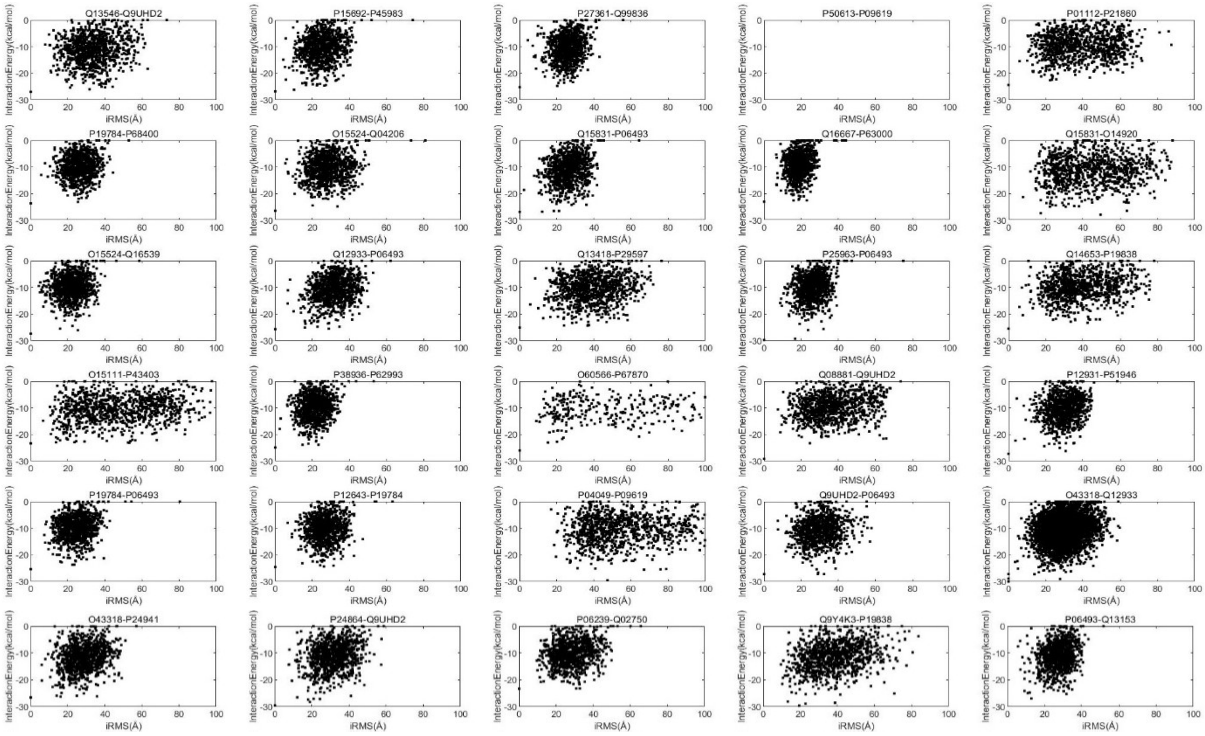

Figure S2: Interaction energy distribution of 105 kinase-random partner pairs, docking simulated using RosettaDock.

Interaction energy of 1000 simulated docking structures of 105 kinase-random partner pairs. Protein dockings were simulated with RosettaDock. Each kinase and random partner uniport ID is indicated above. Interaction energies were calculated with Rosetta energy. iRMS were calculated between each simulated docking structure and the most stable docking structure. To compare distributions, the scale was fixed (iRMS: 0–100Å, Interaction energy: −30 to 0 kcal/mol). Points outside this range were ignored; related to Figure 1 and 3.

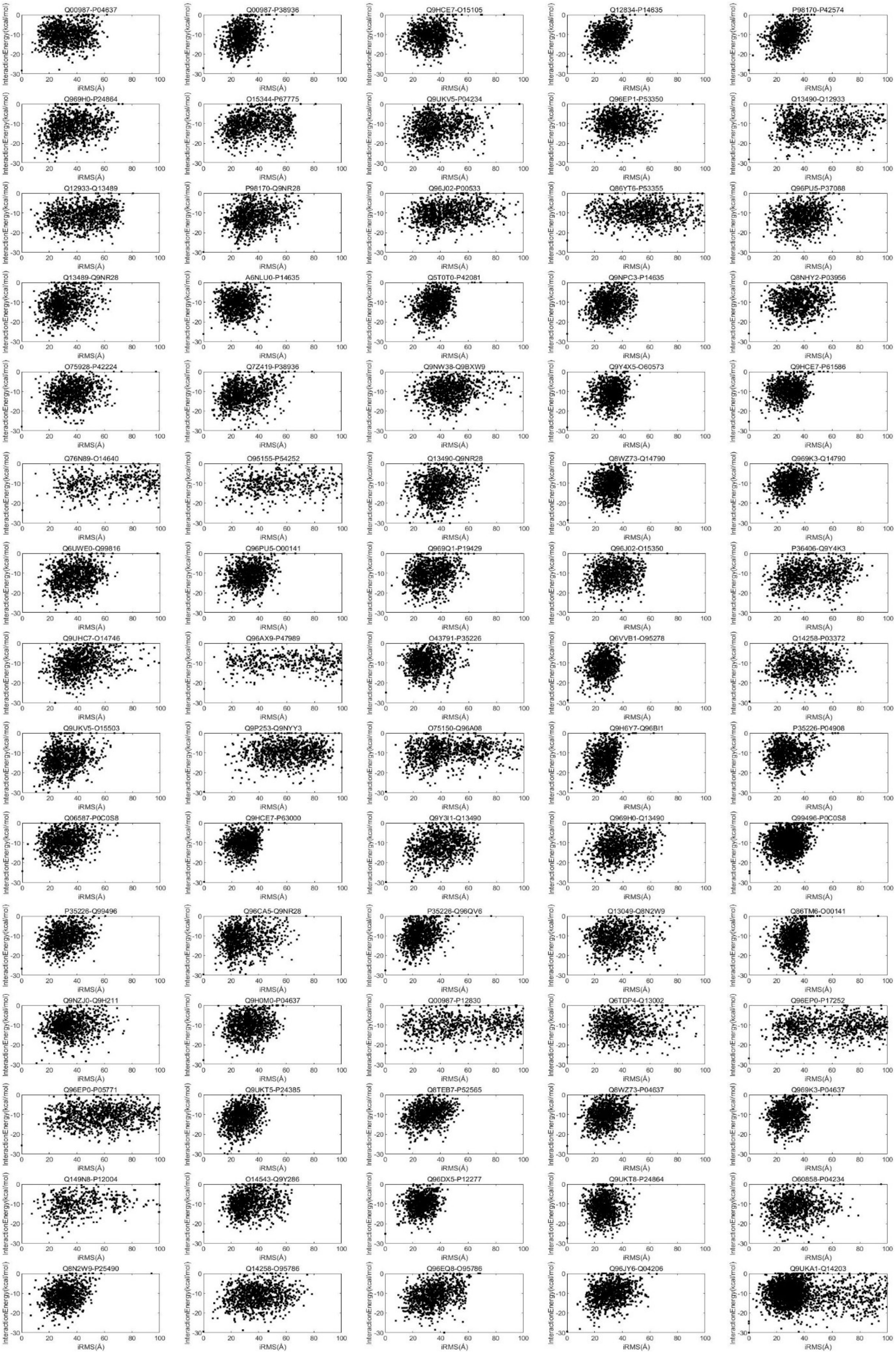
Q00987-P04637
Q00987-P38936
Q9HCE7-O15105
Q12834-P14635
P98170-P42574
Q969H0-P24864
O15344-P67775
Q9UKV5-P04234
Q96EP1-P53350
Q13490-Q12933
Q12933-Q13489
P98170-Q9NR28
Q96J02-P00533
Q86YT6-P53355
Q96PU5-P37088
Q13489-Q9NR28
A6NLU0-P14635
Q5T0T0-P42081
Q9NPC3-P14635
Q8NHY2-P03956
O75928-P42224
Q7Z419-P38936
Q9NW38-Q9BXW9
Q9Y4X5-O60573
Q9HCE7-P61586
Q76N89-O14640
O95155-P54252
Q13490-Q9NR28
Q8WZ73-Q14790
Q969K3-Q14790
Q6UWE0-Q99816
Q96PU5-O00141
Q969Q1-P19429
Q96J02-O15350
P36406-Q9Y4K3
Q9UHC7-O14746
Q96AX9-P47989
O43791-P35226
Q6VVB1-O95278
Q14258-P03372
Q9UKV5-O15503
Q9P253-Q9NYY3
O75150-Q96A08
Q9H6Y7-Q96BI1
P35226-P04908
O06587-P0C0S8
Q9HCE7-P63000
Q9Y3I1-Q13490
Q969H0-Q13490
Q99496-P0C0S8
P35226-Q99496
Q96CA5-Q9NR28
P35226-Q96QV6
Q13049-Q8N2W9
Q86TM6-O00141
Q9NZJ0-Q9H211
Q9H0M0-P04637
Q00987-P12830
Q6TDP4-Q13002
Q96EP0-P17252
Q96EP0-P05771
Q9UKT5-P24385
Q8TEB7-P52565
Q8WZ73-P04637
Q969K3-P04637
Q149N8-P12004
O14543-Q9Y286
Q96DX5-P12277
Q9UKT8-P24864
O60858-P04234
Q8N2W9-P25490
Q14258-O95786
Q96EQ8-O95786
Q96JY6-Q04206
Q9UKA1-Q14203
InteractionEnergy(kcal/mol)
iRMS(Å)

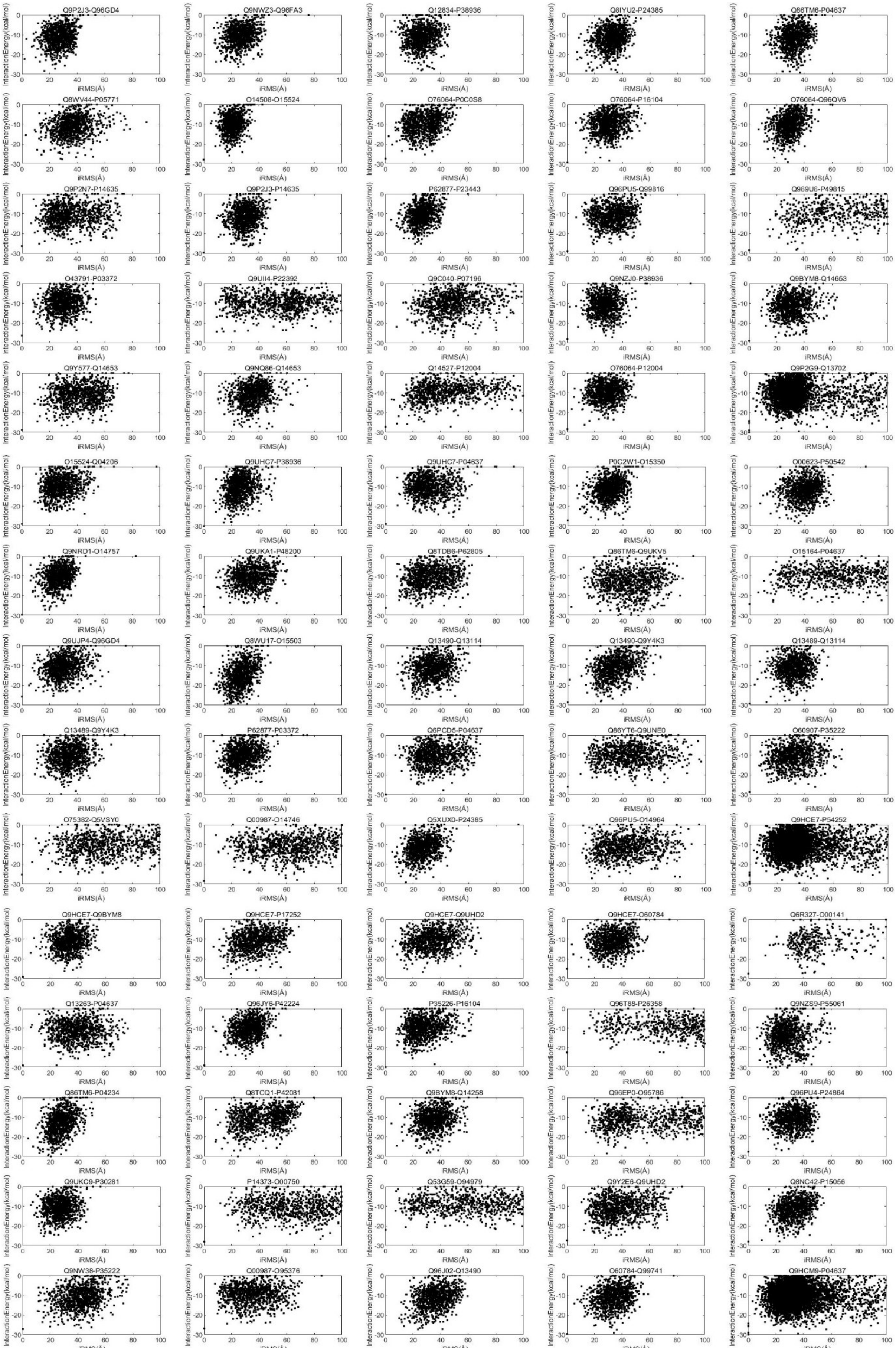
Q9P2J3-Q96GD4
Q9NWZ3-Q96FA3
Q12834-P38936
Q8IYU2-P24385
Q86TM6-P04637
Q8WV44-P05771
O14508-O15524
O76064-P0C0S8
O76064-P16104
O76064-Q96QV6
Q9P2N7-P14635
Q9P2J3-P14635
P62877-P23443
Q96PU5-Q99816
Q969U6-P49815
O43791-P03372
Q9UII4-P22392
Q9C040-P07196
Q9NZJ0-P38936
Q9BYM8-Q14653
Q9Y577-Q14653
Q9NQ86-Q14653
Q14527-P12004
O76064-P12004
Q9P2G9-Q13702
O15524-Q04206
Q9UHC7-P38936
Q9UHC7-P04637
P0C2W1-O15350
O00623-P50542
Q9NRD1-O14757
Q9UKA1-P48200
Q8TDB6-P62805
Q86TM6-Q9UKV5
O15164-P04637
Q9UJP4-Q96GD4
Q8WU17-O15503
Q13490-Q13114
Q13490-Q9Y4K3
Q13489-Q13114
Q13489-Q9Y4K3
P62877-P03372
Q6PCD5-P04637
Q86YT6-Q9UNE0
O60907-P35222
O75382-Q5VSY0
Q00987-O14746
Q5XUX0-P24385
Q96PU5-O14964
Q9HCE7-P54252
Q9HCE7-Q9BYM8
Q9HCE7-P17252
Q9HCE7-Q9UHD2
Q9HCE7-O60784
Q6R327-O00141
Q13263-P04637
Q96JY6-P42224
P35226-P16104
Q96T88-P26358
Q9NZS9-P55061
Q86TM6-P04234
Q8TCQ1-P42081
Q9BYM8-Q14258
Q96EP0-O95786
Q96PU4-P24864
Q9UKC9-P30281
P14373-O00750
Q53G59-O94979
Q9Y2E6-Q9UHD2
Q8NC42-P15056
Q9NW38-P35222
Q00987-O95376
Q96J02-Q13490
O60784-Q99741
Q9HCM9-P04637
InteractionEnergy(kcal/mol)
iRMS(Å)

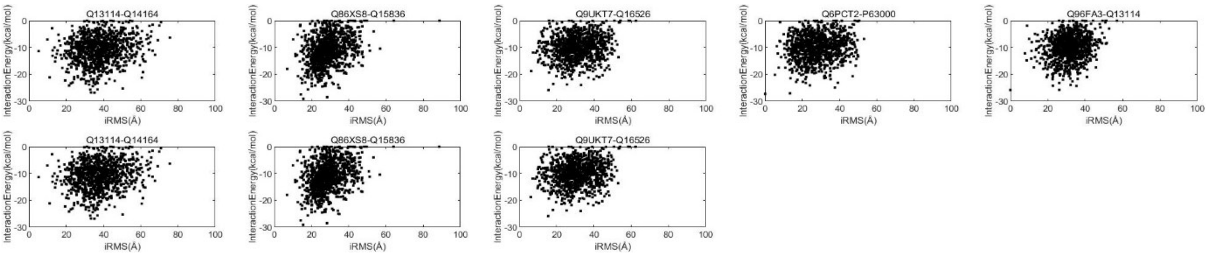

Figure S3: Interaction energy distribution of 158 E3 ubiquitin ligase-substrate pairs, docking simulated using RosettaDock.

Interaction energy of 1000 simulated docking structures of 158 E3 ubiquitin ligase-substrate pairs. Protein dockings were simulated with RosettaDock. Each E3 ubiquitin ligase and substrate uniport ID is indicated above. Interaction energies were calculated with Rosetta energy. iRMS were calculated between each simulated docking structure and the most stable docking structure. To compare distributions, the scale was fixed (iRMS: 0–100Å, Interaction energy: −30 to 0 kcal/mol). Points outside this range were ignored; related to Figure 1 and 3.

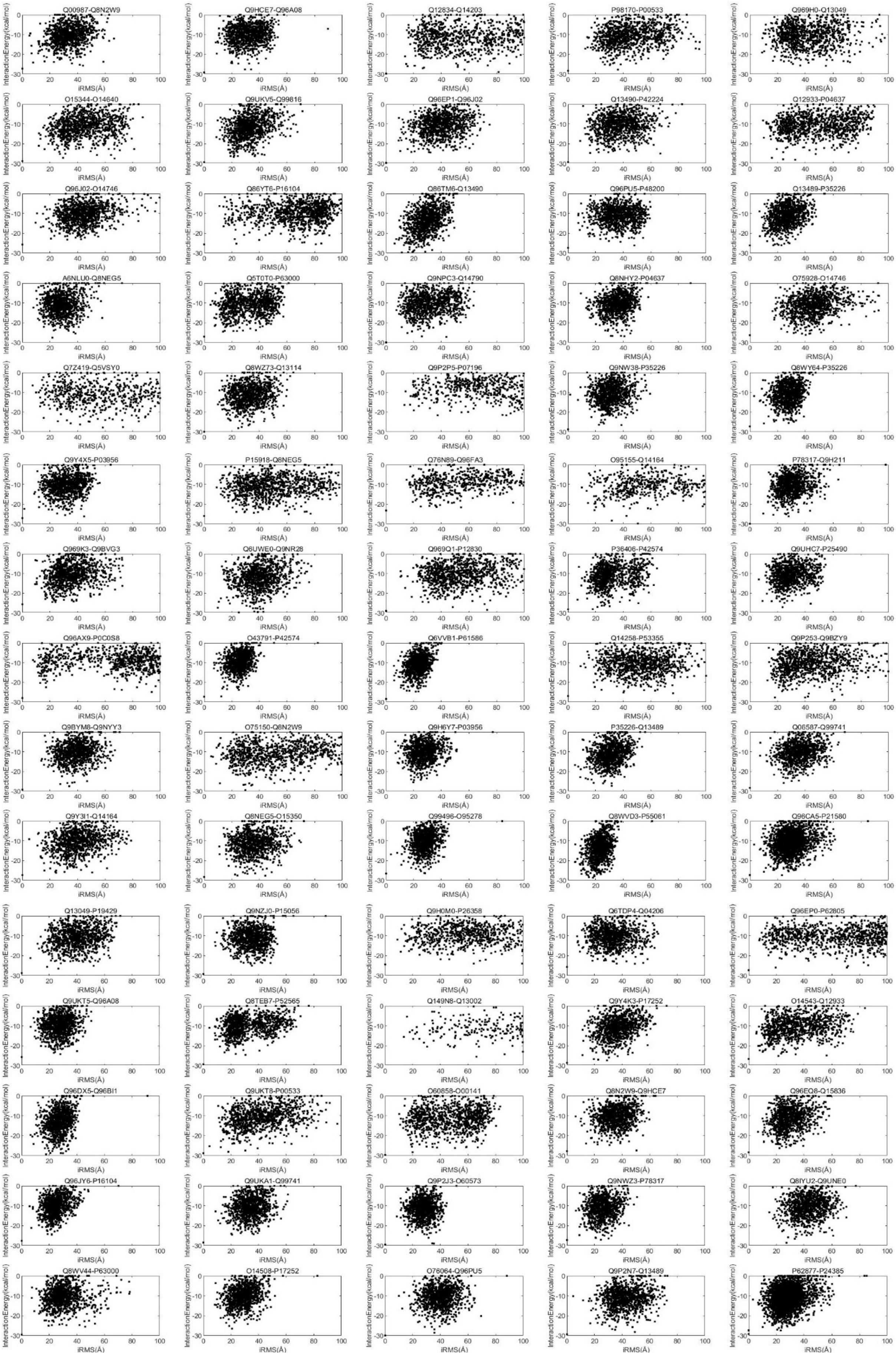
Q00987-Q8N2W9
Q9HCE7-Q96A08
Q12834-Q14203
P98170-P00533
Q969H0-Q13049
O15344-O14640
Q9UKV5-Q99816
Q96EP1-Q96J02
Q13490-P42224
Q12933-P04637
Q96J02-O14746
Q86YT6-P16104
Q86TM6-Q13490
Q96PU5-P48200
Q13489-P35226
A6NLU0-Q8NEG5
Q5T0T0-P63000
Q9NPC3-Q14790
Q8NHY2-P04637
O75928-O14746
Q7Z419-Q5VSY0
Q8WZ73-Q13114
Q9P2P5-P07196
Q9NW38-P35226
Q8WY64-P35226
Q9Y4X5-P03956
P15918-Q8NEG5
Q76N89-Q96FA3
O95155-Q14164
P78317-Q9H211
Q969K3-Q9BVG3
Q6UWE0-Q9NR28
Q969Q1-P12830
P36406-P42574
Q9UHC7-P25490
Q96AX9-P0C0S8
O43791-P42574
Q6VVB1-P61586
Q14258-P53355
Q9P253-Q9BZY9
Q9BYM8-Q9NYY3
O75150-Q8N2W9
Q9H6Y7-P03956
P35226-Q13489
Q06587-Q99741
Q9Y3I1-Q14164
Q8NEG5-O15350
Q99496-O95278
Q8WVD3-P55061
Q96CA5-P21580
Q13049-P19429
Q9NZJ0-P15056
Q9H0M0-P26358
Q6TDP4-Q04206
Q96EP0-P62805
Q9UKT5-Q96A08
Q8TEB7-P52565
Q149N8-Q13002
Q9Y4K3-P17252
O14543-Q12933
Q96DX5-Q96BI1
Q9UKT8-P00533
O60858-O00141
Q8N2W9-Q9HCE7
Q96EQ8-O15836
Q96JY6-P16104
Q9UKA1-Q99741
Q9P2J3-O60573
Q9NWZ3-P78317
Q8IYU2-Q9UNE0
Q8WV44-P63000
O14508-P17252
O76064-Q96PU5
Q9P2N7-Q13489
P62877-P24385
InteractionEnergy(kcal/mol)
iRMS(Å)

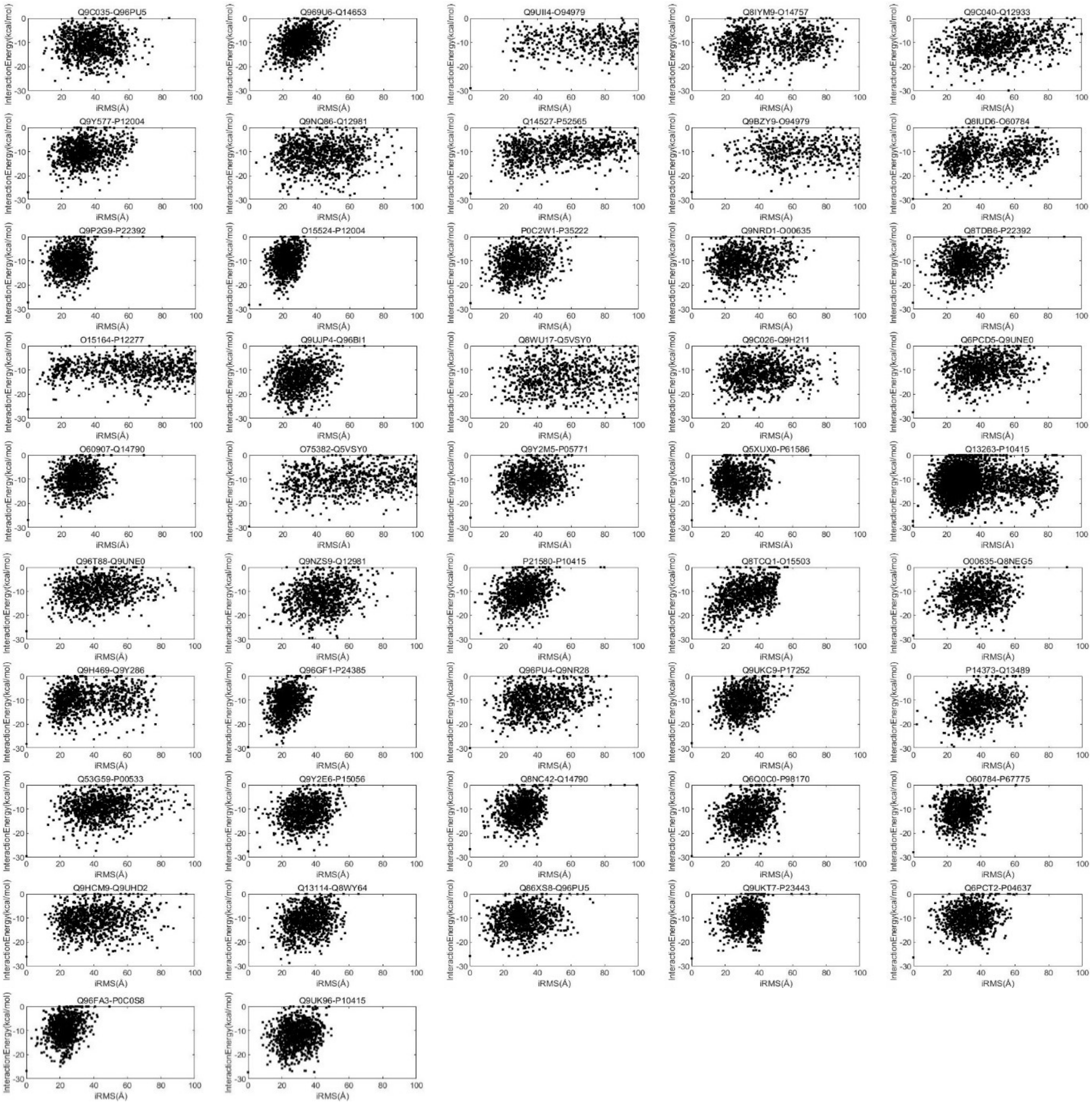

Figure S4: Interaction energy distribution of 122 E3 ubiquitin ligase-random substrate pairs, docking simulated using RosettaDock.

Interaction energy of 1000 simulated docking structures of 122 E3 ubiquitin ligase-random substrate pairs. Protein dockings were simulated with RosettaDock. Each E3 ubiquitin ligase and substrate uniport ID is indicated above. Interaction energies were calculated with Rosetta energy. iRMS were calculated between each simulated docking structure and the most stable docking structure. To compare distributions, the scale was fixed (iRMS: 0–100Å, Interaction energy: −30 to 0 kcal/mol). Points outside this range were ignored; related to Figure 1 and 3.

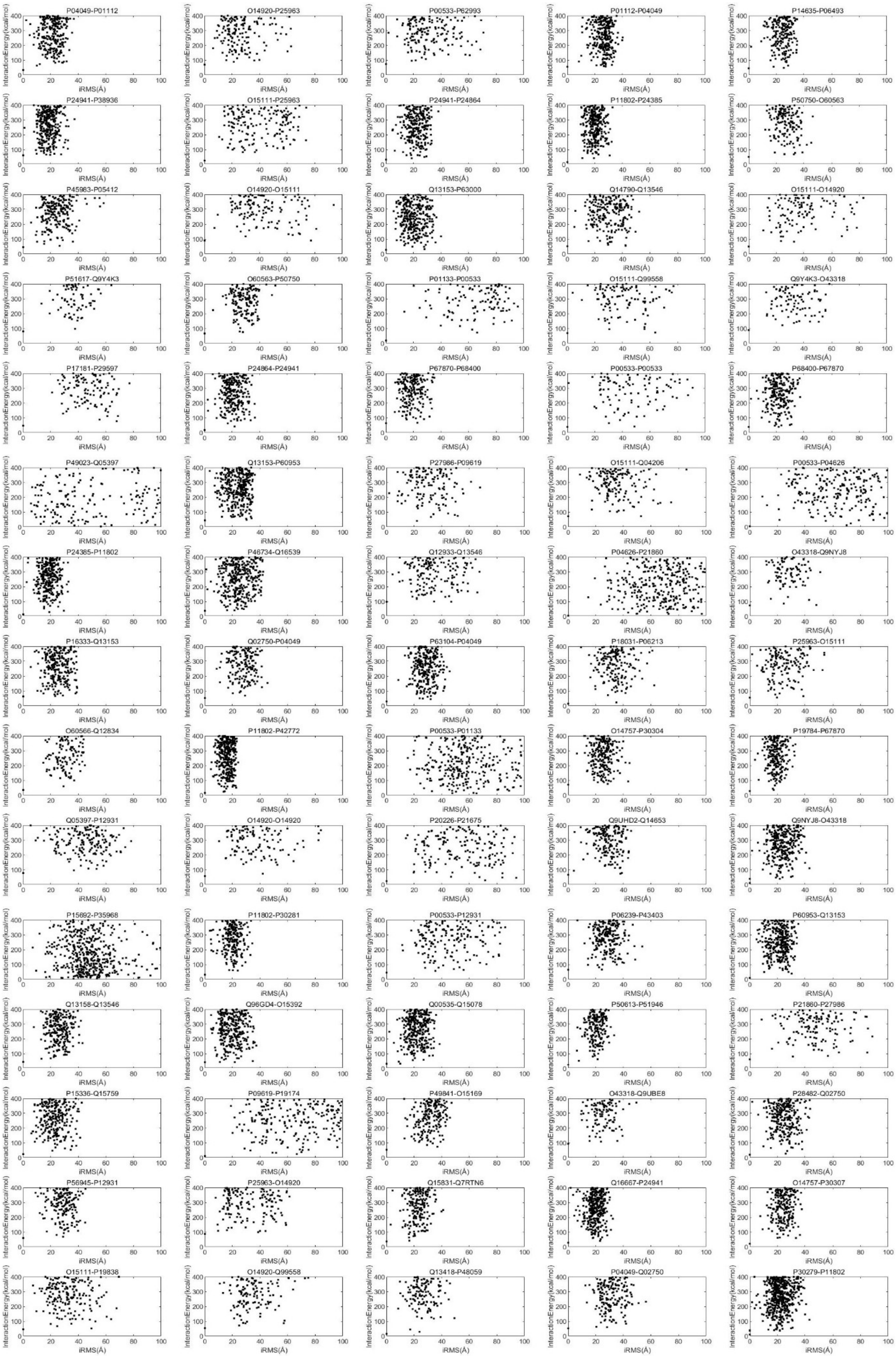
P04049-P01112
O14920-P25963
P00533-P62993
P01112-P04049
P14635-P06493
P24941-P38936
O15111-P25963
P24941-P24864
P11802-P24385
P50750-O60563
P45983-P05412
O14920-O15111
Q13153-P63000
Q14790-Q13546
O15111-O14920
P51617-Q9Y4K3
O60563-P50750
P01133-P00533
O15111-Q99558
Q9Y4K3-O43318
P17181-P29597
P24864-P24941
P67870-P68400
P00533-P00533
P68400-P67870
P49023-Q05397
Q13153-P60953
P27986-P09619
O15111-Q04206
P00533-P04626
P24385-P11802
P46734-Q16539
Q12933-Q13546
P04626-P21860
O43318-Q9NYJ8
P16333-Q13153
Q02750-P04049
P63104-P04049
P18031-P06213
P25963-O15111
O60566-Q12834
P11802-P42772
P00533-P01133
O14757-P30304
P19784-P67870
Q05397-P12931
O14920-O14920
P20226-P21675
Q9UHD2-Q14653
Q9NYJ8-O43318
P15692-P35968
P11802-P30281
P00533-P12931
P06239-P43403
P60953-Q13153
Q13158-Q13546
Q96GD4-O15392
Q00535-Q15078
P50613-P51946
P21860-P27986
P15336-Q15759
P09619-P19174
P49841-O15169
O43318-Q9UBE8
P28482-Q02750
P56945-P12931
P25963-O14920
Q15831-Q7RTN6
Q16667-P24941
O14757-P30307
O15111-P19838
O14920-Q99558
Q13418-P48059
P04049-Q02750
P30279-P11802
InteractionEnergy(kcal/mol)
iRMS(Å)

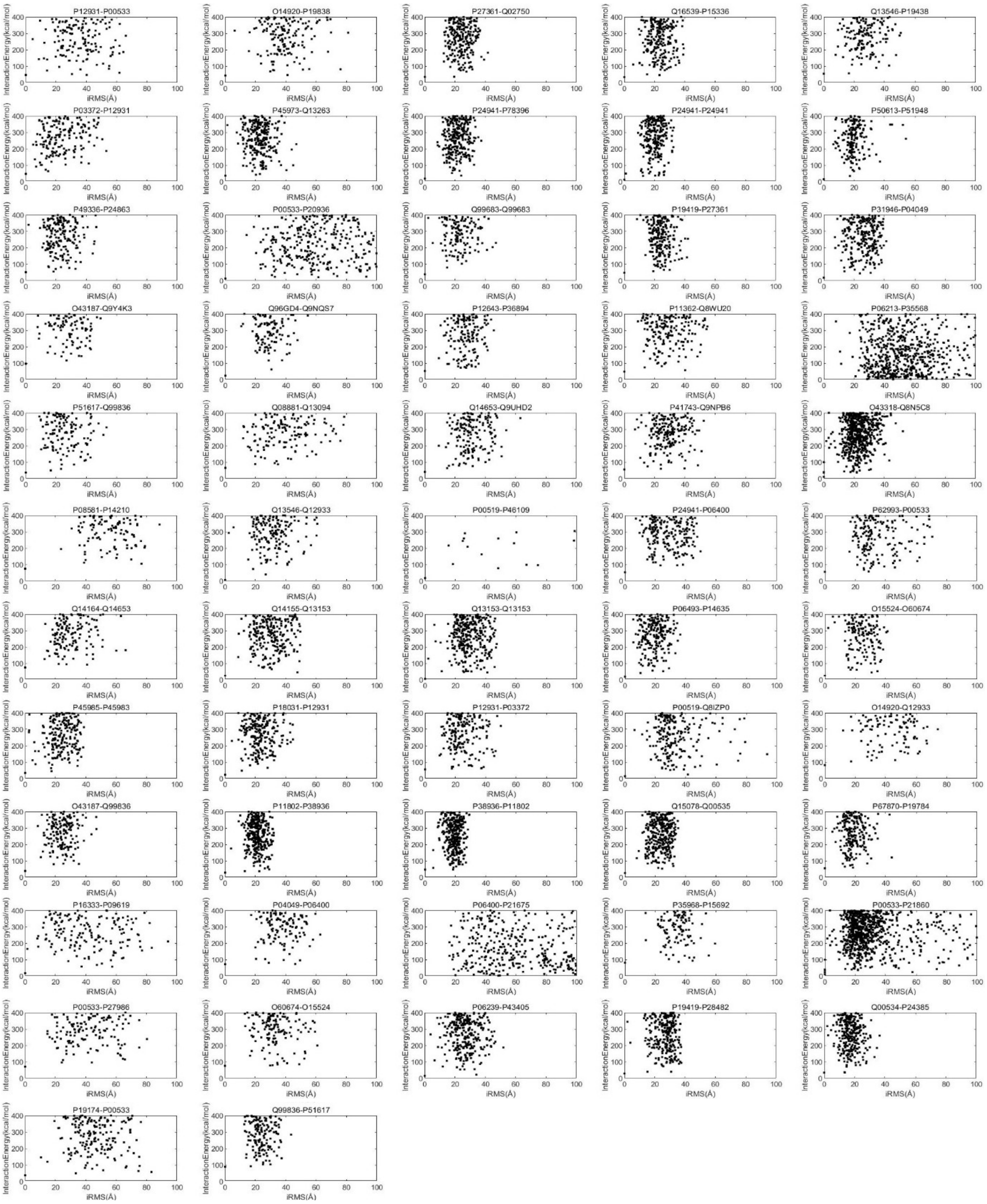

Figure S5: Interaction energy distribution of 132 kinase-specific interacting partner pairs, docking simulated using HDOCKlite.

Interaction energy of 1000 simulated docking structures of 132 kinase-specific interacting partner pairs. Protein dockings were simulated with HDOCKlite. Each kinase and specific interacting partner uniport ID are indicated above. Interaction energies were calculated with Rosetta energy. iRMS were calculated between each simulated docking structure and the most stable docking structure. To compare distributions, the scale was fixed (iRMS: 0–100Å, Interaction energy: 0–400 kcal/mol). Points outside this range were ignored; related to Figure 1 and 3.

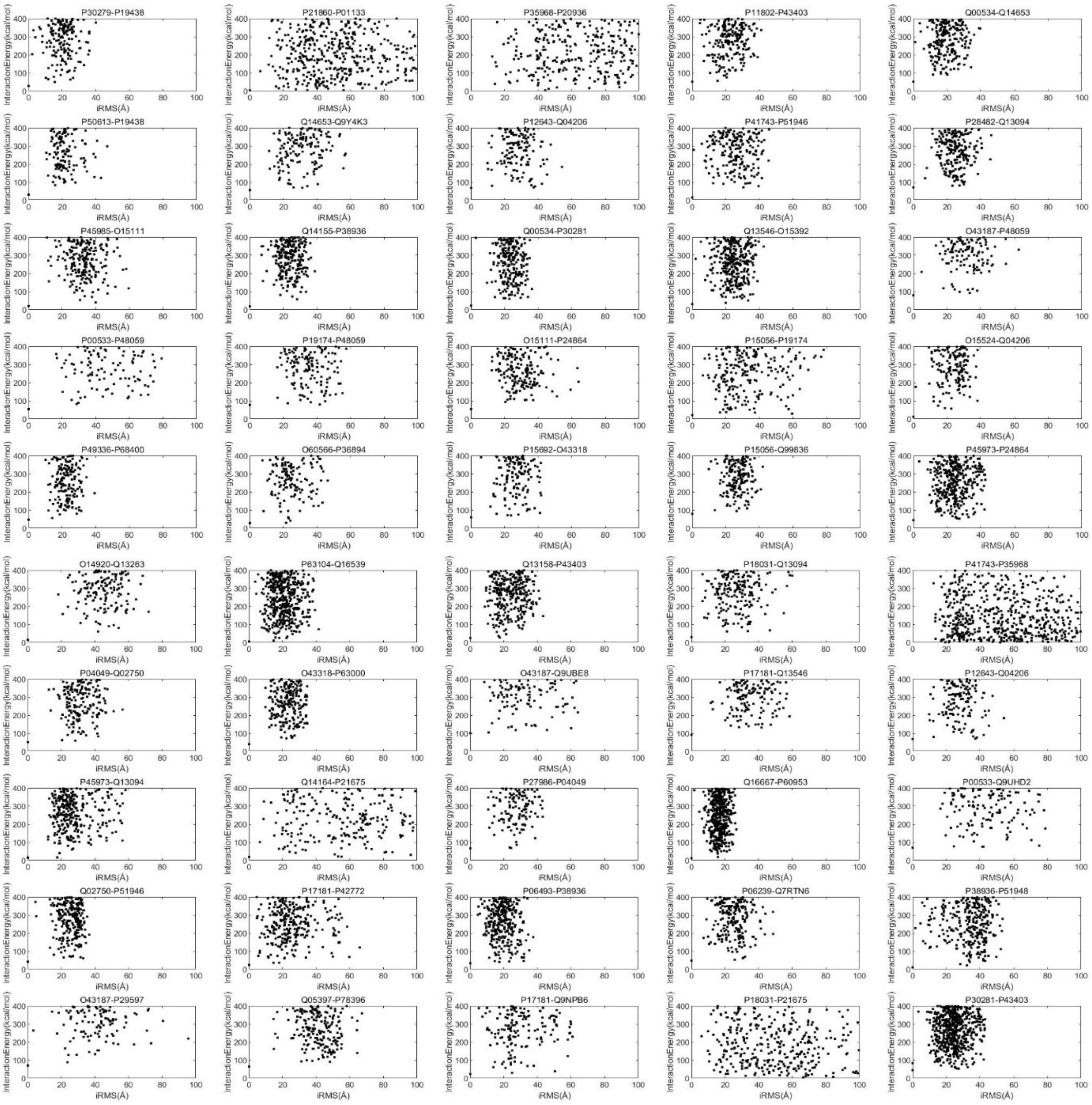
P30279-P19438
P21860-P01133
P35968-P20936
P11802-P43403
Q00534-Q14653
P50613-P19438
Q14653-Q9Y4K3
P12643-Q04206
P41743-P51946
P28482-Q13094
P45985-O15111
Q14155-P38936
Q00534-P30281
Q13546-O15392
O43187-P48059
P00533-P48059
P19174-P48059
O15111-P24864
P15056-P19174
O15524-Q04206
P49336-P68400
O60566-P36894
P15692-O43318
P15056-Q99836
P45973-P24864
O14920-Q13283
P63104-Q16539
Q13158-P43403
P18031-Q13094
P41743-P35968
P04049-Q02750
O43318-P63000
O43187-Q9UBE8
P17181-Q13546
P12643-Q04206
P45973-Q13094
Q14164-P21675
P27986-P04049
Q16667-P60953
P00533-Q9UHD2
Q02750-P51946
P17181-P42772
P06493-P38936
P06239-Q7RTN6
P38936-P51948
O43187-P29597
Q05397-P78396
P17181-Q9NPB6
P18031-P21675
P30281-P43403
InteractionEnergy(kcal/mol)
iRMS(Å)

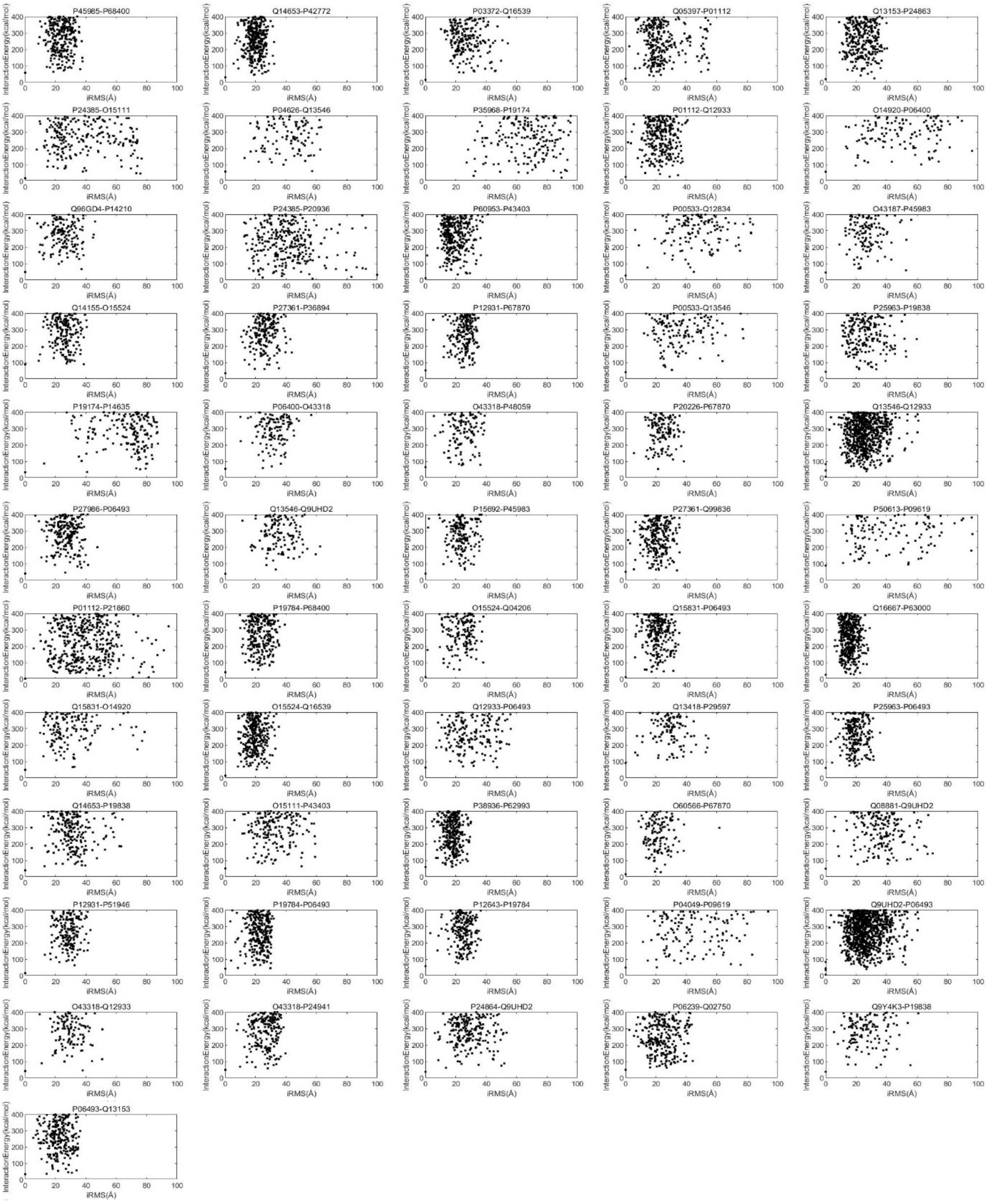

Figure S6: Interaction energy distribution of 106 kinase-random partner pairs, docking simulated using HDOCKlite.

Interaction energy of 1000 simulated docking structures of 106 kinase-random partner pairs. Protein dockings were simulated with HDOCKlite. Each kinase and partner uniport ID is indicated above. Interaction energies were calculated with Rosetta energy. iRMS were calculated between each simulated docking structure and the most stable docking structure. To compare distributions, the scale was fixed (iRMS: 0–100Å, Interaction energy: 0–400 kcal/mol). Points outside this range were ignored; related to Figure 1 and 3.

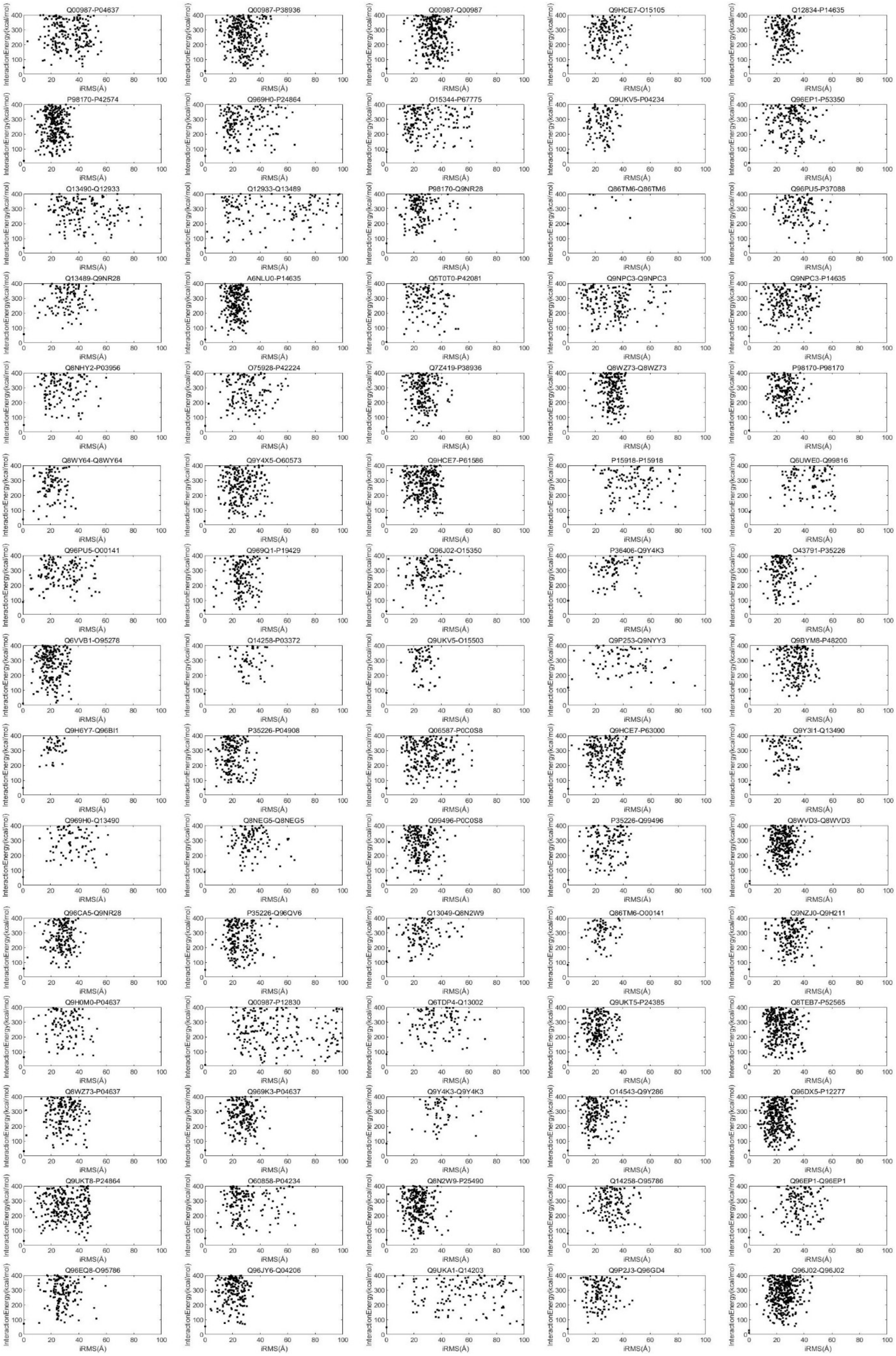
Q00987-P04637
Q00987-P38936
Q00987-Q00987
Q9HCE7-O15105
Q12834-P14635
P98170-P42574
Q969H0-P24864
O15344-P67775
Q9UKV5-P04234
Q96EP1-P53350
Q13490-Q12933
Q12933-Q13489
P98170-Q9NR28
Q86TM6-Q86TM6
Q96PU5-P37088
Q13489-Q9NR28
A6NLU0-P14635
Q5T0T0-P42081
Q9NPC3-Q9NPC3
Q9NPC3-P14635
Q8NHY2-P03956
O75928-P42224
Q7Z419-P38936
Q8WZ73-Q8WZ73
P98170-P98170
Q8WY64-Q8WY64
Q9Y4X5-O60573
Q9HCE7-P61586
P15918-P15918
Q6UWE0-Q99816
Q96PU5-O00141
Q969Q1-P19429
Q96J02-O15350
P36406-Q9Y4K3
O43791-P35226
Q6VVB1-O95278
Q14258-P03372
Q9UKV5-O15503
Q9P253-Q9NYY3
Q9BYM8-P48200
Q9H6Y7-Q96BI1
P35226-P04908
Q06587-P0C0S8
Q9HCE7-P63000
Q9Y3I1-Q13490
Q969H0-Q13490
Q8NEG5-Q8NEG5
Q99496-P0C0S8
P35226-Q99496
Q8WVD3-Q8WVD3
Q96CA5-Q9NR28
P35226-Q96QV6
Q13049-Q8N2W9
Q86TM6-O00141
Q9NZJ0-Q9H211
Q9H0M0-P04637
Q00987-P12830
Q6TDP4-Q13002
Q9UKT5-P24385
Q8TEB7-P52565
Q8WZ73-P04637
Q969K3-P04637
Q9Y4K3-Q9Y4K3
O14543-Q9Y286
Q96DX5-P12277
Q9UKT8-P24864
O60858-P04234
Q8N2W9-P25490
Q14258-O95786
Q96EP1-Q96EP1
Q96EQ8-O95786
Q96JY6-Q04206
Q9UKA1-Q14203
Q9P2J3-Q96GD4
Q96J02-Q96J02
InteractionEnergy(kcal/mol)
iRMS(Å)

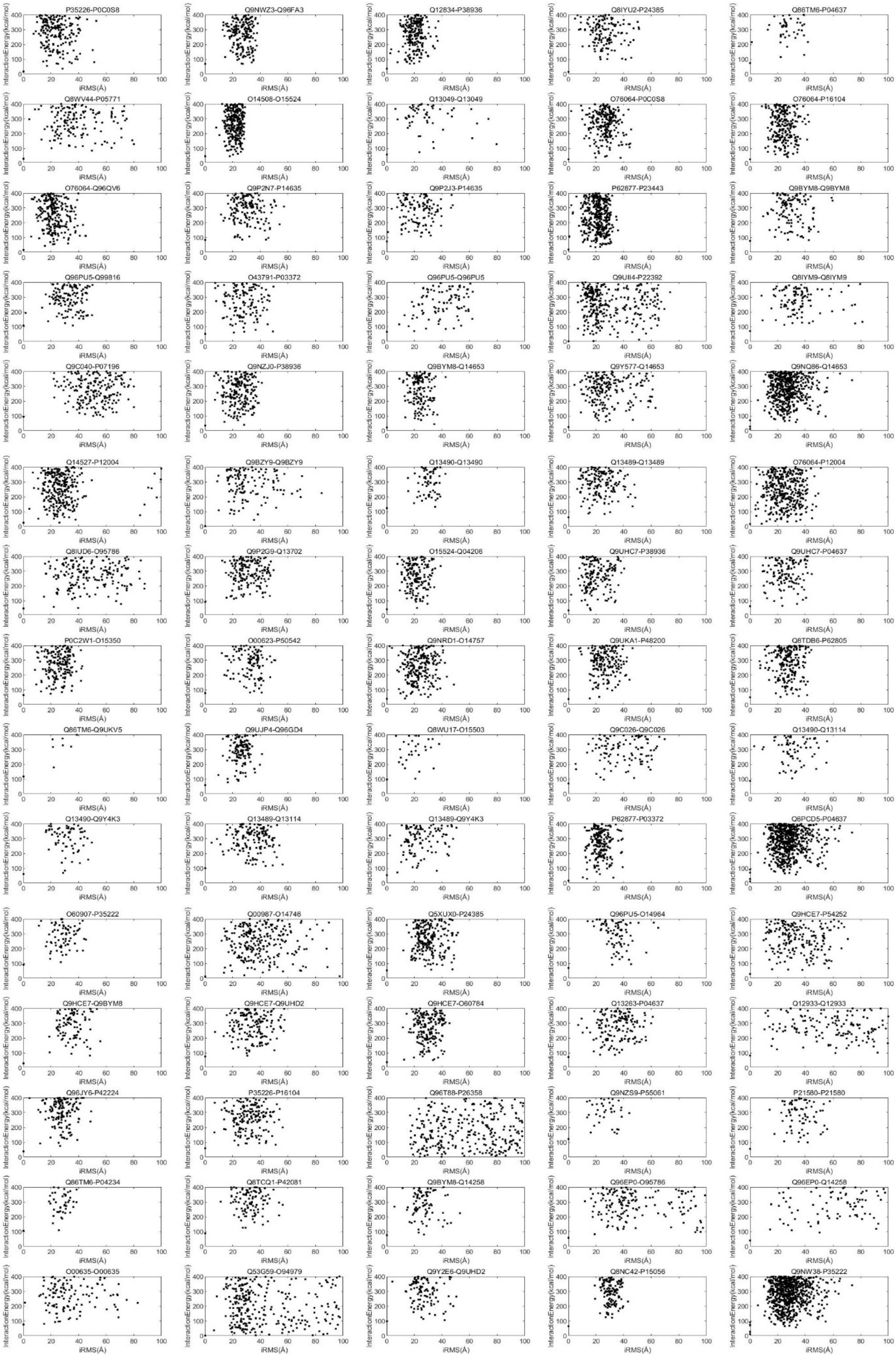
P35226-P0C0S8
Q9NWZ3-Q96FA3
Q12834-P38936
Q8IYU2-P24385
Q86TM6-P04637
Q8WV44-P05771
O14508-O15524
Q13049-Q13049
O76064-P0C0S8
O76064-P16104
O76064-Q96QV6
Q9P2N7-P14635
Q9P2J3-P14635
P62877-P23443
Q9BYM8-Q9BYM8
Q96PU5-Q99816
O43791-P03372
Q96PU5-Q96PU5
Q9UII4-P22392
Q8IYM9-Q8IYM9
Q9C040-P07196
Q9NZJ0-P38936
Q9BYM8-Q14653
Q9Y577-Q14653
Q9NQ86-Q14653
Q14527-P12004
Q9BZY9-Q9BZY9
Q13490-Q13490
Q13489-Q13489
O76064-P12004
Q8IUD6-O95786
Q9P2G9-Q13702
O15524-Q04206
Q9UHC7-P38936
Q9UHC7-P04637
P0C2W1-O15350
O00623-P50542
Q9NRD1-O14757
Q9UKA1-P48200
Q8TDB6-P62805
Q86TM6-Q9UKV5
Q9UJP4-Q96GD4
Q8WU17-O15503
Q9C026-Q9C026
Q13490-Q13114
Q13490-Q9Y4K3
Q13489-Q13114
Q13489-Q9Y4K3
P62877-P03372
Q6PCD5-P04637
O60907-P35222
Q00987-O14746
Q5XUX0-P24385
Q96PU5-O14964
Q9HCE7-P54252
Q9HCE7-Q9BYM8
Q9HCE7-Q9UHD2
Q9HCE7-O60784
Q13263-P04637
Q12933-Q12933
Q96JY6-P42224
P35226-P16104
Q96T88-P26358
Q9NZS9-P55061
P21580-P21580
Q86TM6-P04234
Q8TCQ1-P42081
Q9BYM8-Q14258
Q96EP0-O95786
Q96EP0-Q14258
O00635-O00635
Q53G59-O94979
Q9Y2E6-Q9UHD2
Q8NC42-P15056
Q9NW38-P35222
InteractionEnergy(kcal/mol)
iRMS(Å)

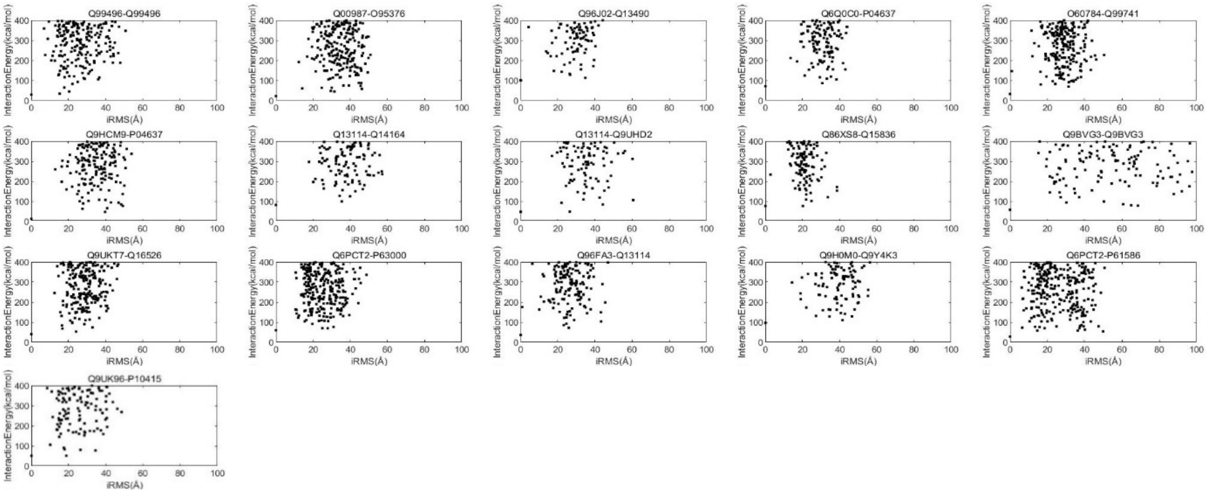

Figure S7: Interaction energy distribution of 166 E3 ubiquitin ligase-substrate pairs, docking simulated using HDOCKlite.

Interaction energy of 1000 simulated docking structures of 166 E3 ubiquitin ligase-substrate pairs. Protein dockings were simulated with HDOCKlite. Each E3 ubiquitin ligase and substrate uniport ID is indicated above. Interaction energies were calculated with Rosetta energy. iRMS were calculated between each simulated docking structure and the most stable docking structure. To compare distributions, the scale was fixed (iRMS: 0–100Å, Interaction energy: 0–400 kcal/mol). Points outside this range were ignored; related to Figure 1 and 3.

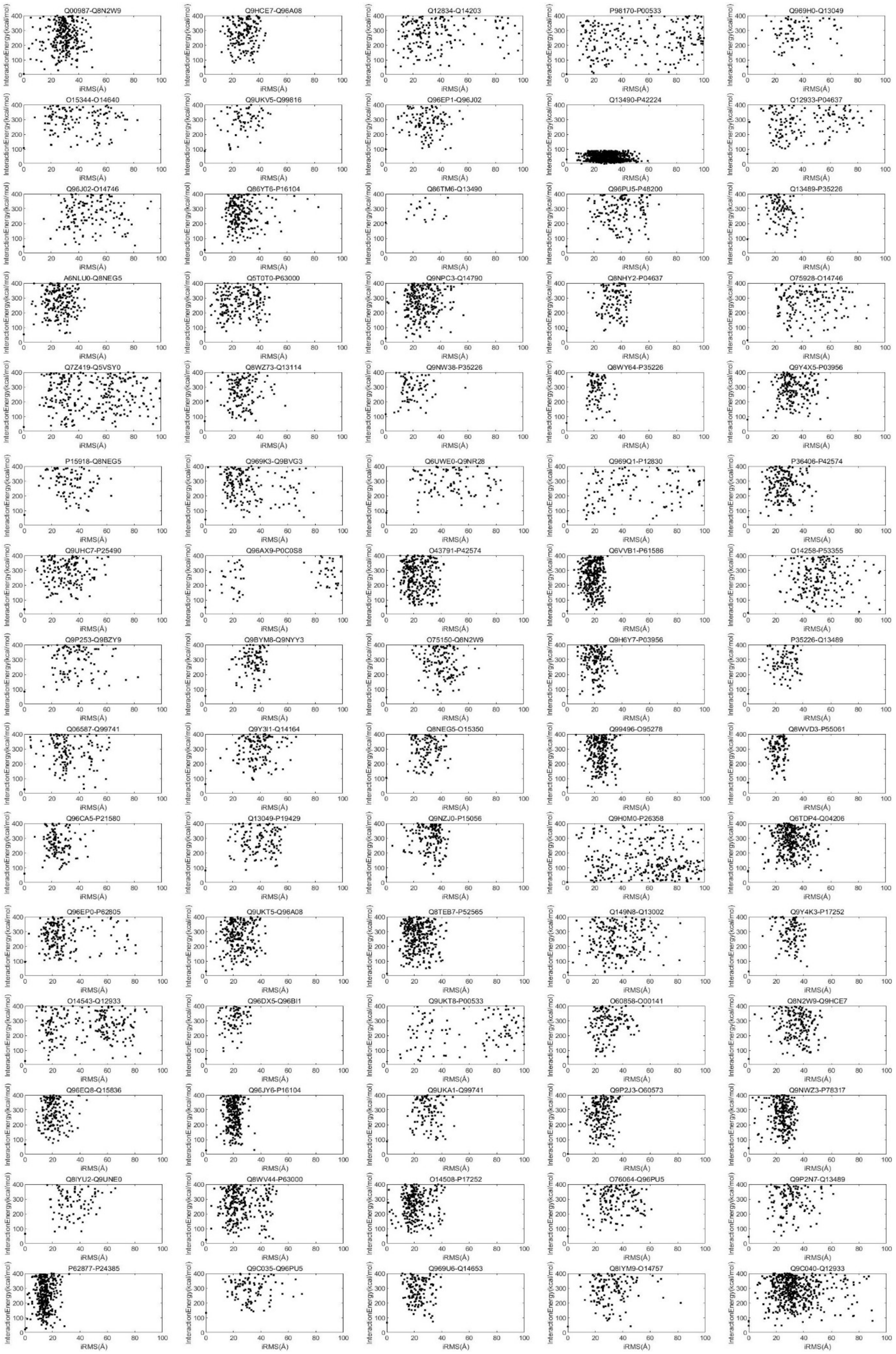
Q00987-Q8N2W9
Q9HCE7-Q96A08
Q12834-Q14203
P98170-P00533
Q969H0-Q13049
O15344-O14640
Q9UKV5-Q99816
Q96EP1-Q96J02
Q13490-P42224
Q12933-P04637
Q96J02-O14746
Q86YT6-P16104
Q86TM6-Q13490
Q96PU5-P48200
Q13489-P35226
A6NLU0-Q8NEG5
Q5T0T0-P63000
Q9NPC3-Q14790
Q8NHY2-P04637
O75928-O14746
Q7Z419-Q5VSY0
Q8WZ73-Q13114
Q9NW38-P35226
Q8WY64-P35226
Q9Y4X5-P03956
P15918-Q8NEG5
Q969K3-Q9BVG3
Q6UWE0-Q9NR28
Q969Q1-P12830
P36406-P42574
Q9UHC7-P25490
Q96AX9-P0C0S8
O43791-P42574
Q6VVB1-P61586
Q14258-P53355
Q9P253-Q9BZY9
Q9BYM8-Q9NYY3
O75150-Q8N2W9
Q9H6Y7-P03956
P35226-Q13489
Q06587-Q99741
Q9Y3I1-Q14164
Q8NEG5-O15350
Q99496-O95278
Q8WVD3-P55061
Q96CA5-P21580
Q13049-P19429
Q9NZJ0-P15056
Q9H0M0-P26358
Q6TDP4-Q04206
Q96EP0-P62805
Q9UKT5-Q96A08
Q8TEB7-P52565
Q149N8-Q13002
Q9Y4K3-P17252
O14543-Q12933
Q96DX5-Q96BI1
Q9UKT8-P00533
O60858-O00141
Q8N2W9-Q9HCE7
Q96EQ8-Q15836
Q96JY6-P16104
Q9UKA1-Q99741
Q9P2J3-O60573
Q9NWZ3-P78317
Q8IYU2-Q9UNE0
Q8WV44-P63000
O14508-P17252
O76064-Q96PU5
Q9P2N7-Q13489
P62877-P24385
Q9C035-Q96PU5
Q969U6-Q14653
Q8IYM9-O14757
Q9C040-Q12933
InteractionEnergy(kcal/mol)
iRMS(Å)

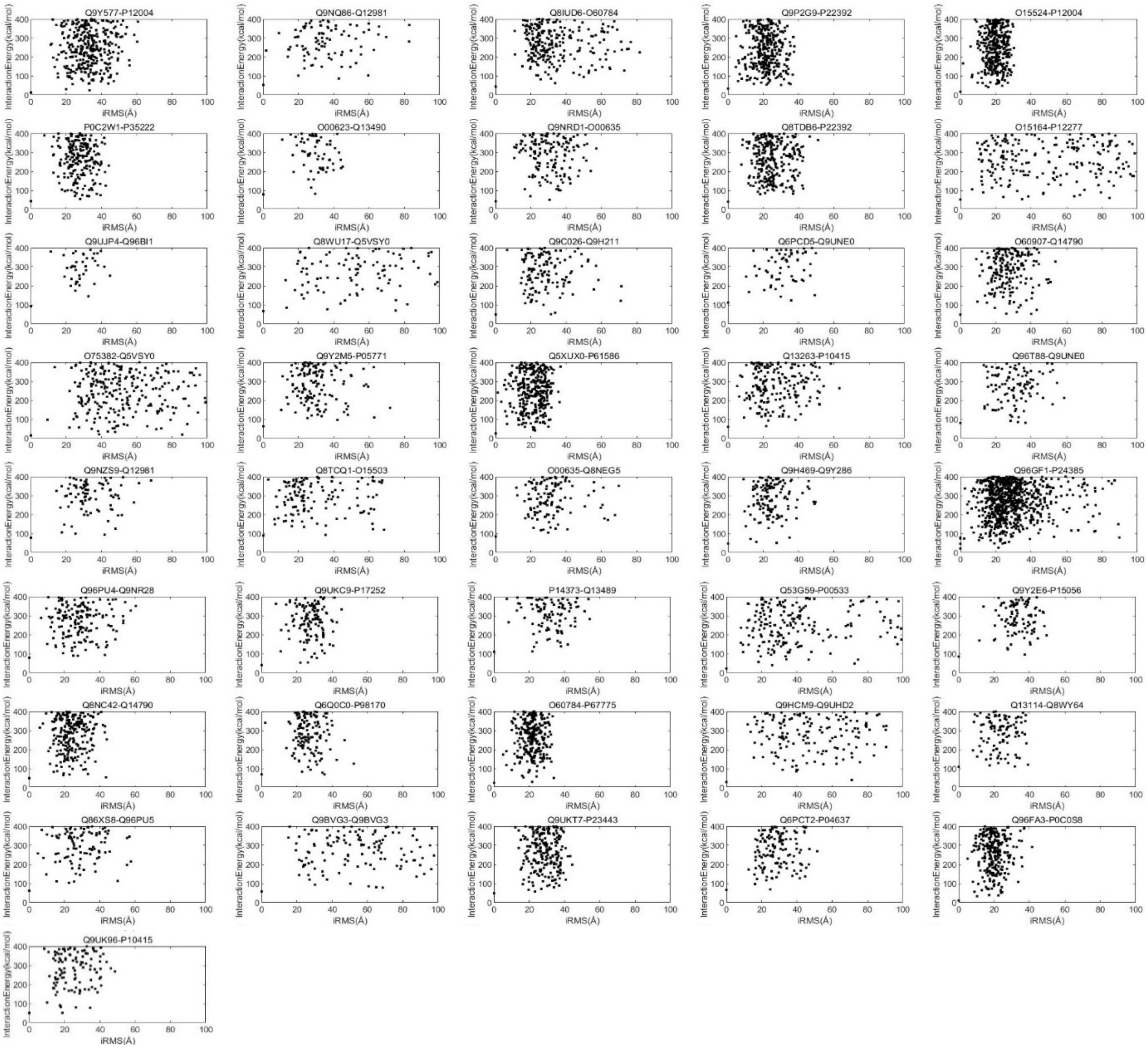

Figure S8: Interaction energy distribution of 116 E3 ubiquitin ligase-random substrate pairs, docking simulated using HDOCKlite.

Interaction energy of 1000 simulated docking structures of 116 E3 ubiquitin ligase-random substrate pairs. Protein dockings were simulated with HDOCKlite. Each E3 ubiquitin ligase and substrate uniport ID is indicated above. Interaction energies were calculated with Rosetta energy. iRMS were calculated between each simulated docking structure and the most stable docking structure. To compare distributions, the scale was fixed (iRMS: 0–100Å, Interaction energy: 0–400 kcal/mol). Points outside this range were ignored; related to Figure 1 and 3.

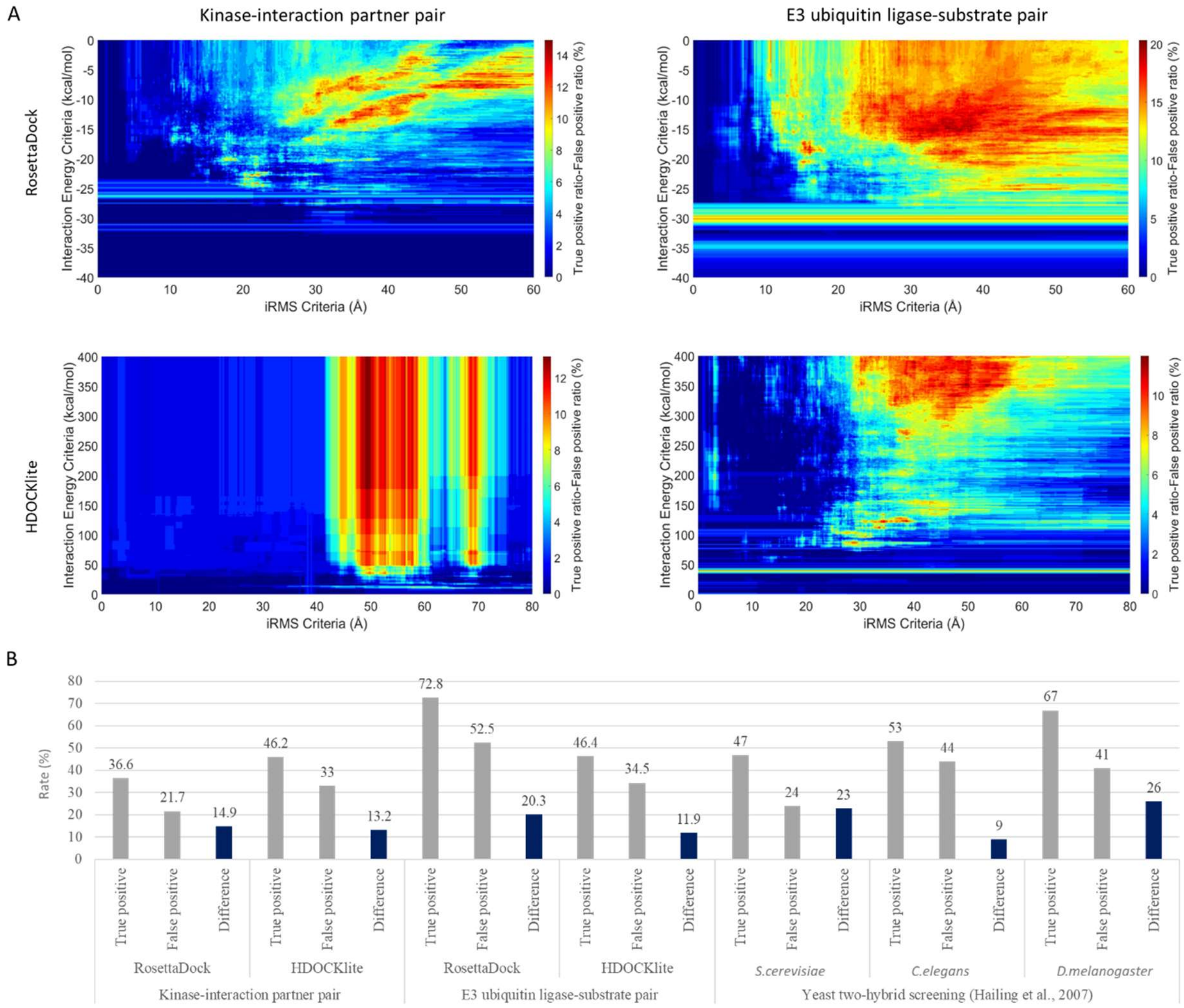

Figure S9: Accuracy of predicting protein interaction by searching narrow funnel-like interaction distribution for whole analyzed kinase and E3 ubiquitin ligase interaction pair.

(A) How well narrow-funnel interaction energy distribution can distinguish specific interaction partner is shown for each criterion. Whole interaction pairs were analyzed. The ratio of difference between true-positive (identified pair with narrow funnel-like interaction energy distribution) and false-positive (random pair with narrow funnel-like interaction energy distribution) for each criterion are shown. Due to the limitation of graphical representation, only maximum of ratio for each distribution criteria are shown. (B) Comparison of rate for finding specific interaction partner using narrow-funnel searching method and yeast two-hybrid screening. Genome-scale yeast two-hybrid analysis for three species were compared[51]. Criteria for finding narrow funnel-like energy distribution are described in Table S1; related to Figure 3.

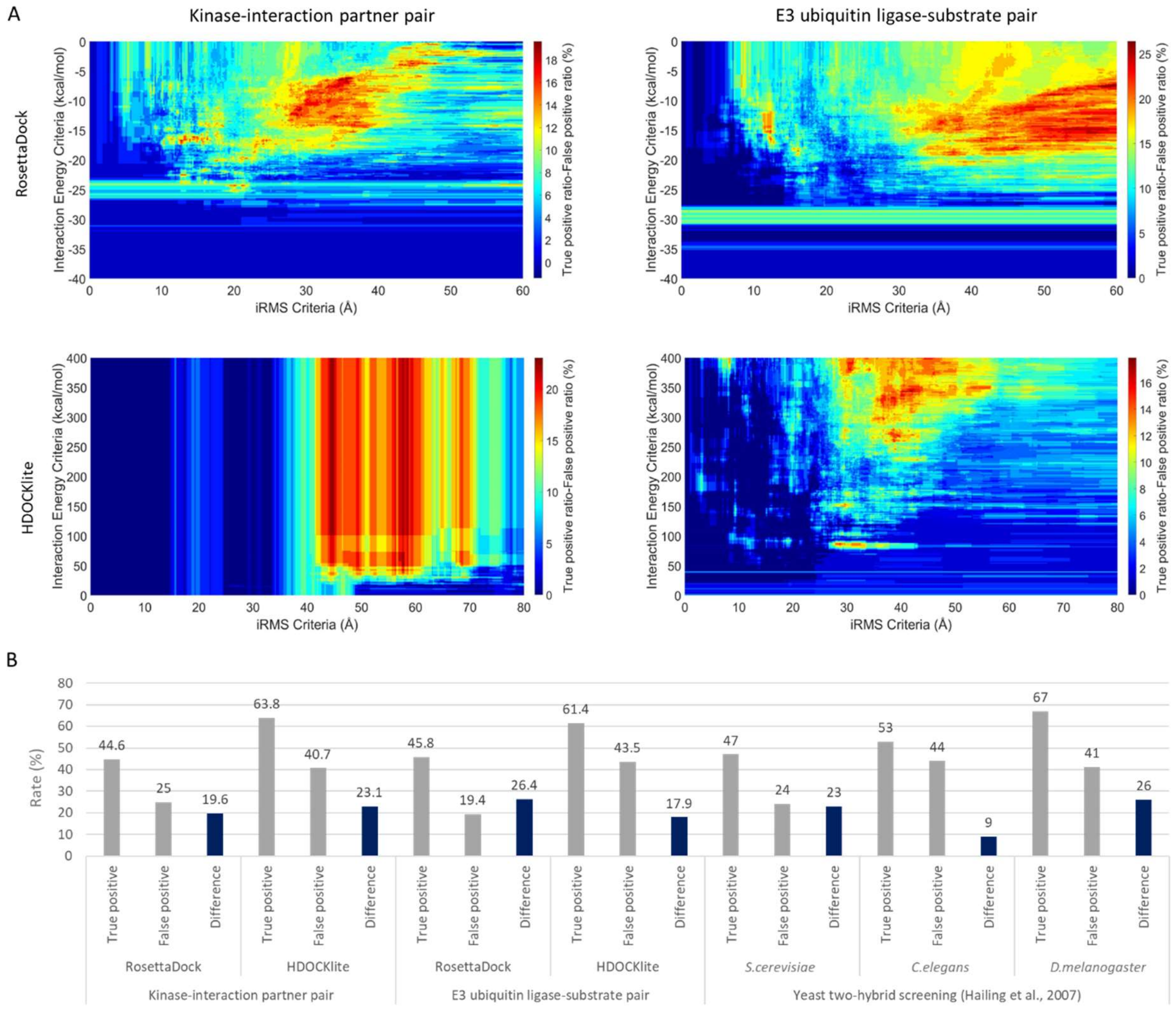

Figure S10: Accuracy of predicting protein interaction by searching narrow funnel-like interaction distribution for kinase and E3 ubiquitin ligase interaction pair with high accuracy for structure prediction.

(A) How well narrow-funnel interaction energy distribution can distinguish specific interaction partner is shown for each criterion. Interaction pairs with high-accuracy structure prediction results (>70% region with high confidence) are analyzed. The ratio of difference between true-positive (identified pair with narrow funnel-like interaction energy distribution) and false-positive (random pair with narrow funnel-like interaction energy distribution) for each criterion are shown. Due to the limitation of graphical representation, only maximum of ratio for each distribution criteria are shown. (B) Comparison of rate for finding specific interaction partner using narrow-funnel searching method and yeast two-hybrid screening. Genome-scale yeast two-hybrid analysis for three species were compared[51]. Criteria for finding narrow funnel-like energy distribution are described in Table S2; related to Figure 3.

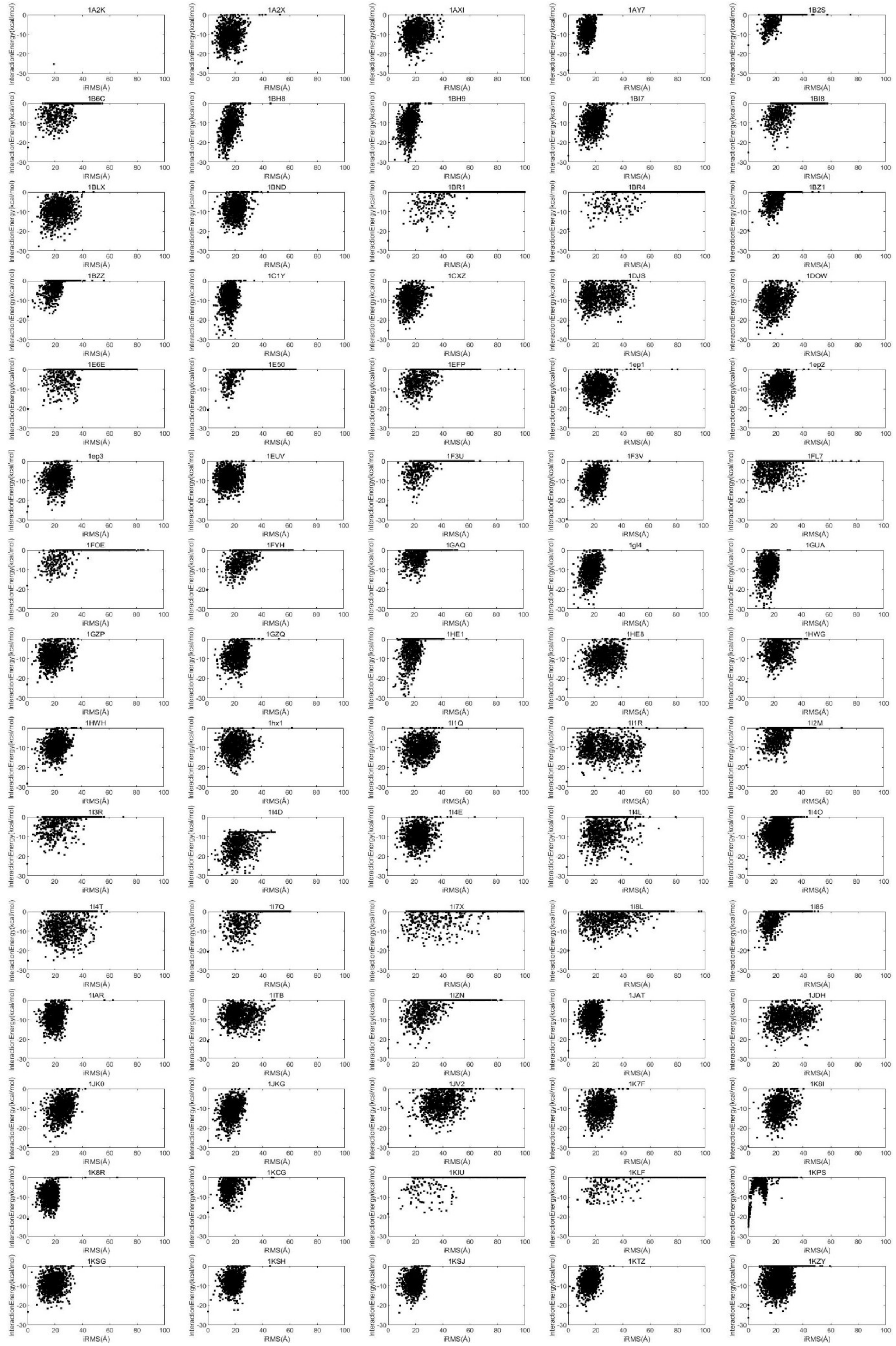

1A2K
1A2X
1AXI
1AY7
1B2S
1B6C
1BH8
1BH9
1BI7
1BI8
1BLX
1BND
1BR1
1BR4
1BZ1
1BZZ
1C1Y
1CXZ
1DJS
1DOW
1E6E
1E50
1EFP
1ep1
1ep2
1ep3
1EUV
1F3U
1F3V
1FL7
1FOE
1FYH
1GAQ
1gl4
1GUA
1GZP
1GZQ
1HE1
1HE8
1HWG
1HWH
1hx1
1I1Q
1I1R
1I2M
1I3R
1I4D
1I4E
1I4L
1I4O
1I4T
1I7Q
1I7X
1I8L
1I85
1IAR
1ITB
1IZN
1JAT
1JDH
1JK0
1JKG
1JV2
1K7F
1K8I
1K8R
1KCG
1KIU
1KLF
1KPS
1KSG
1KSH
1KSJ
1KTZ
1KZY
InteractionEnergy(kcal/mol)
iRMS(Å)

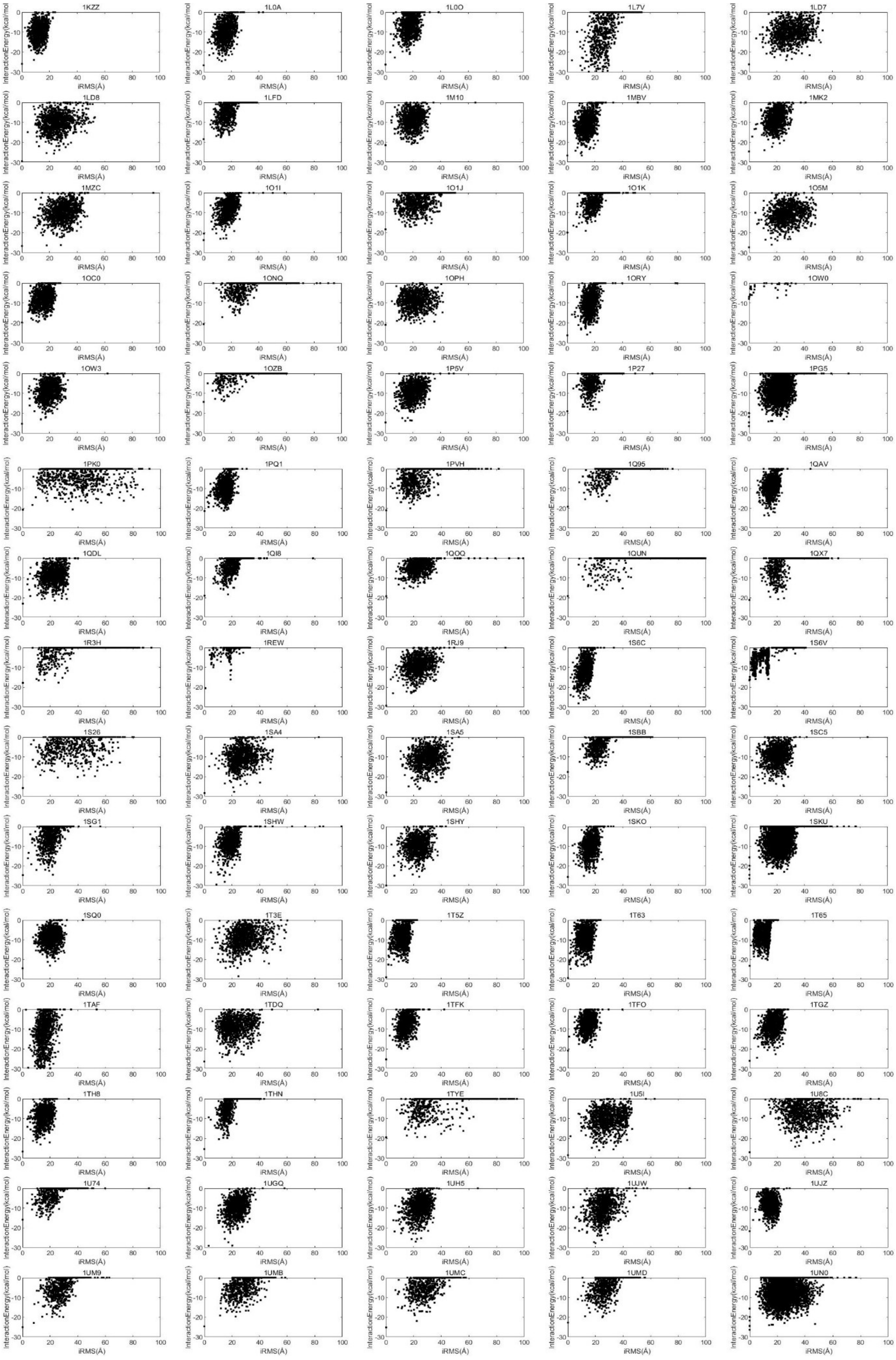
1KZZ
1L0A
1L0O
1L7V
1LD7
1LD8
1LFD
1M10
1MBV
1MK2
1MZC
1O1I
1O1J
1O1K
1O5M
1OC0
1ONQ
1OPH
1ORY
1OW0
1OW3
1OZB
1P5V
1P27
1PG5
1PK0
1PQ1
1PVH
1Q95
1QAV
1QDL
1QI8
1QOQ
1QUN
1QX7
1R3H
1REW
1RJ9
1S6C
1S6V
1S26
1SA4
1SA5
1SBB
1SC5
1SG1
1SHW
1SHY
1SKO
1SKU
1SQ0
1T3E
1T5Z
1T63
1T65
1TAF
1TDQ
1TFK
1TFO
1TGZ
1TH8
1THN
1TYE
1U5I
1U8C
1U74
1UGQ
1UH5
1UJW
1UJZ
1UM9
1UMB
1UMC
1UMD
1UN0
InteractionEnergy(kcal/mol)
iRMS(Å)

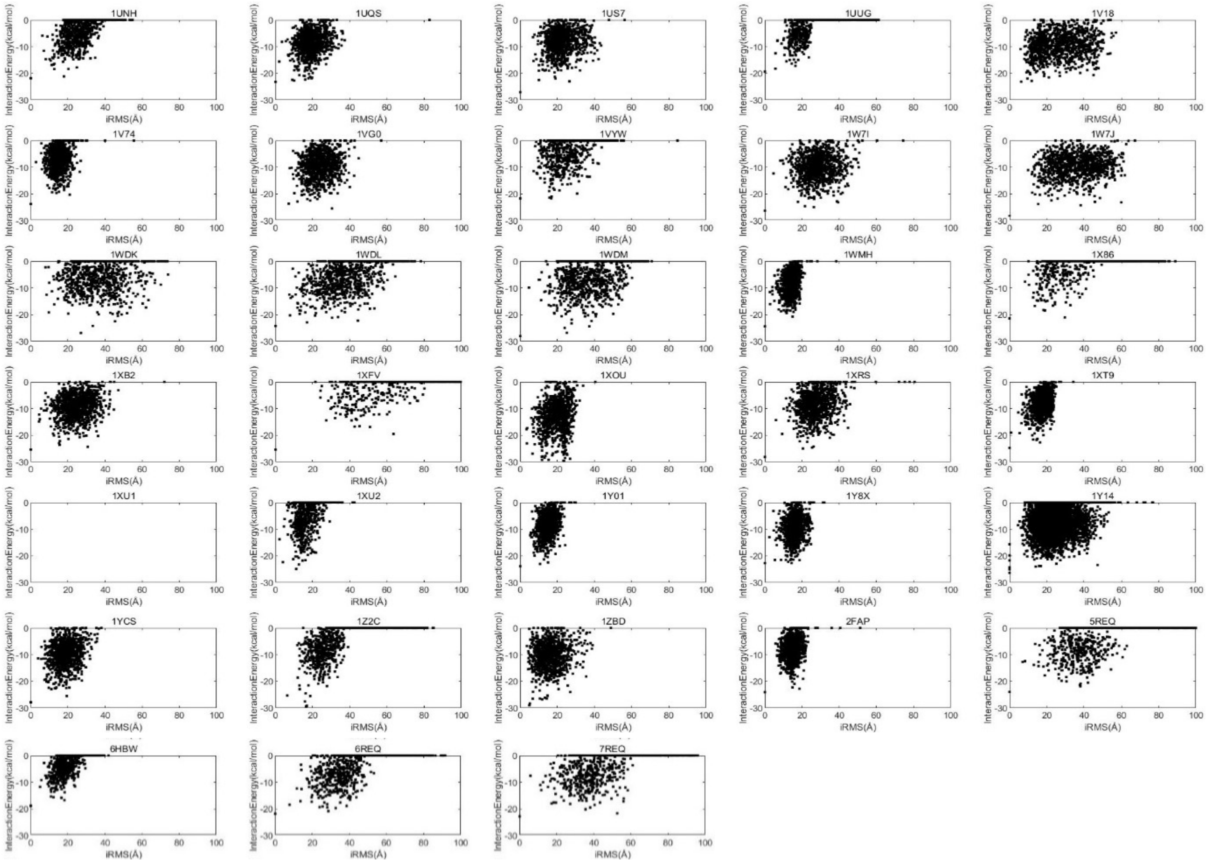

Figure S11: Interaction energy distribution of 183 experimentally determined protein complexes from IntAct database, docking simulated using RosettaDock.

Interaction energy of 1000 simulated docking structures of 183 experimentally determined protein complexes from IntAct database are shown. Protein dockings were simulated with RosettaDock. Each PDB ID of protein complex structure are notated above. Interaction energies were calculated with Rosetta energy. iRMS were calculated between each simulated docking structure and most stable docking structure. To compare distributions, scale is fixed (iRMS: 0–100Å, Interaction energy: −30 to 0 kcal/mol). Points outside this range were ignored; related to Figure 4.

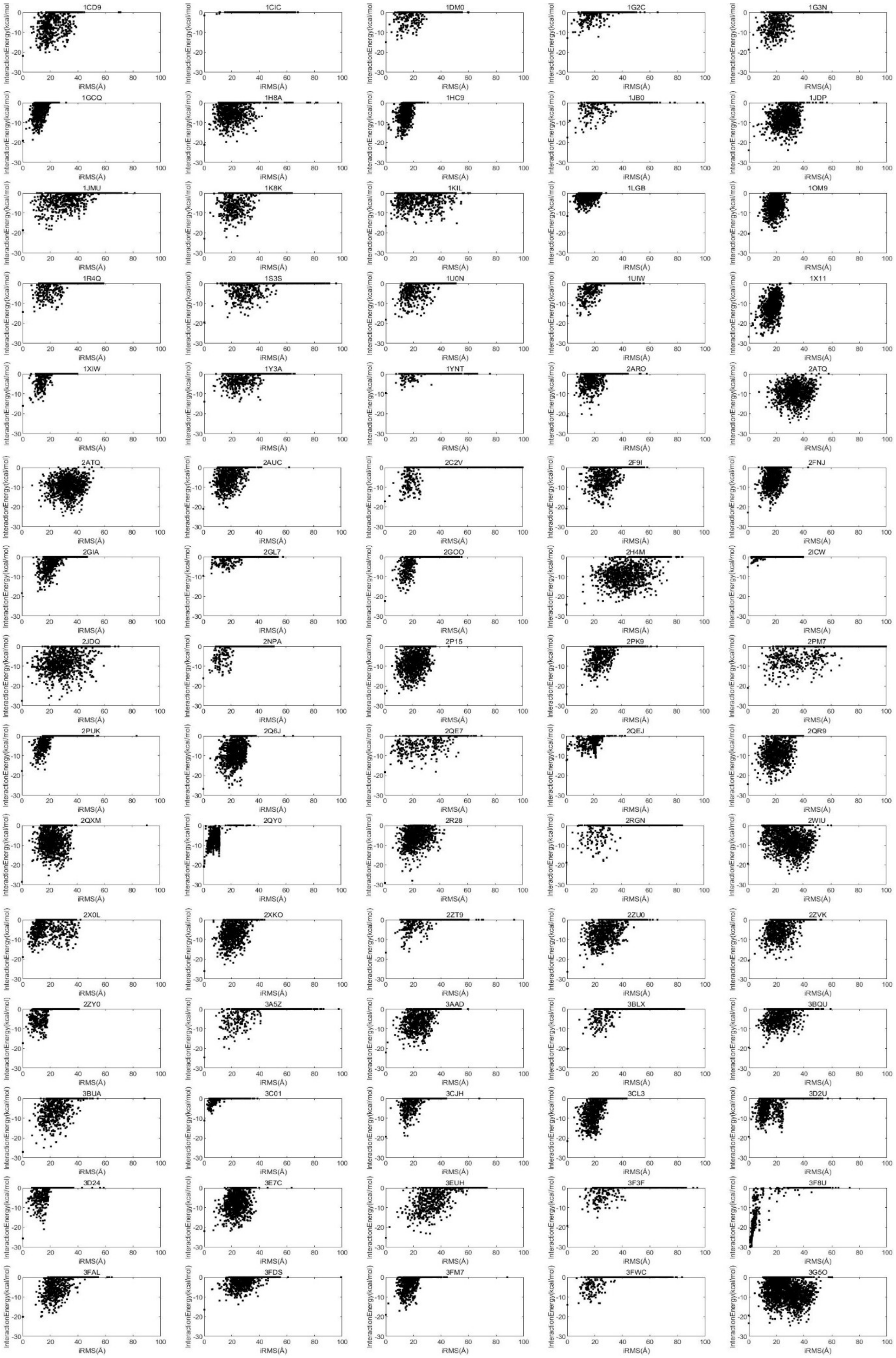
1CD9
1CIC
1DM0
1G2C
1G3N
1GCQ
1H8A
1HC9
1JB0
1JDP
1JMU
1K8K
1KIL
1LGB
1OM9
1R4Q
1S3S
1U0N
1UIW
1X11
1XIW
1Y3A
1YNT
2ARO
2ATQ
2ATQ
2AUC
2C2V
2F9I
2FNJ
2GIA
2GL7
2GOO
2H4M
2ICW
2JDQ
2NPA
2P15
2PK9
2PM7
2PUK
2Q6J
2QE7
2QEJ
2QR9
2QXM
2QY0
2R28
2RGN
2WIU
2X0L
2XKO
2ZT9
2ZU0
2ZVK
2ZY0
3A5Z
3AAD
3BLX
3BQU
3BUA
3C01
3CJH
3CL3
3D2U
3D24
3E7C
3EUH
3F3F
3F8U
3FAL
3FDS
3FM7
3FWC
3G5O
InteractionEnergy(kcal/mol)
iRMS(Å)

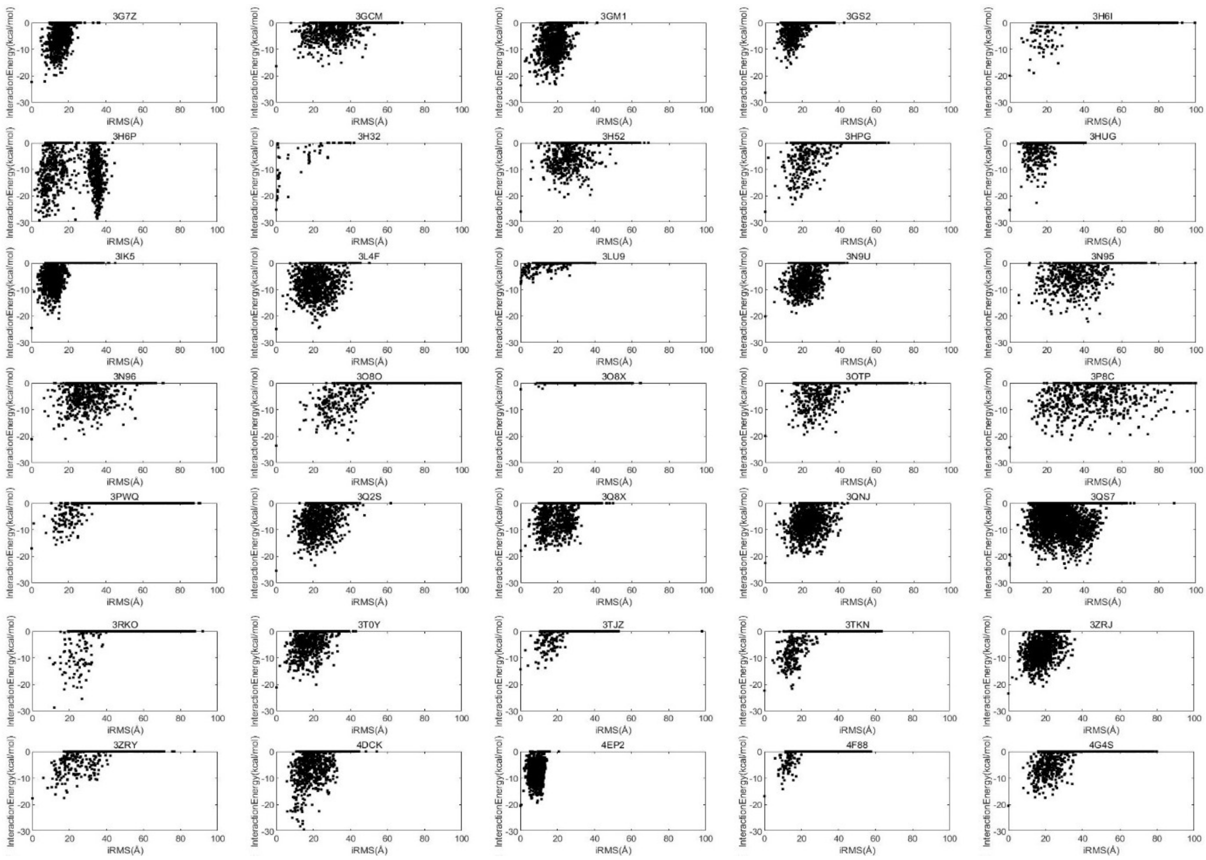

Figure S12: Interaction energy distribution of 110 protein pairs unlikely interact each other from negatome 2.0 database, docking simulated using RosettaDock.

Interaction energy of 1000 simulated docking structures of 110 protein pairs unlikely interact each other from negatome 2.0 database are shown. Protein dockings were simulated with RosettaDock. Each PDB ID of protein complex structure are notated above. Interaction energies were calculated with Rosetta energy. iRMS were calculated between each simulated docking structure and most stable docking structure. To compare distributions, scale is fixed (iRMS: 0–100Å, Interaction energy: −30 to 0 kcal/mol). Points outside this range were ignored; related to Figure 4.

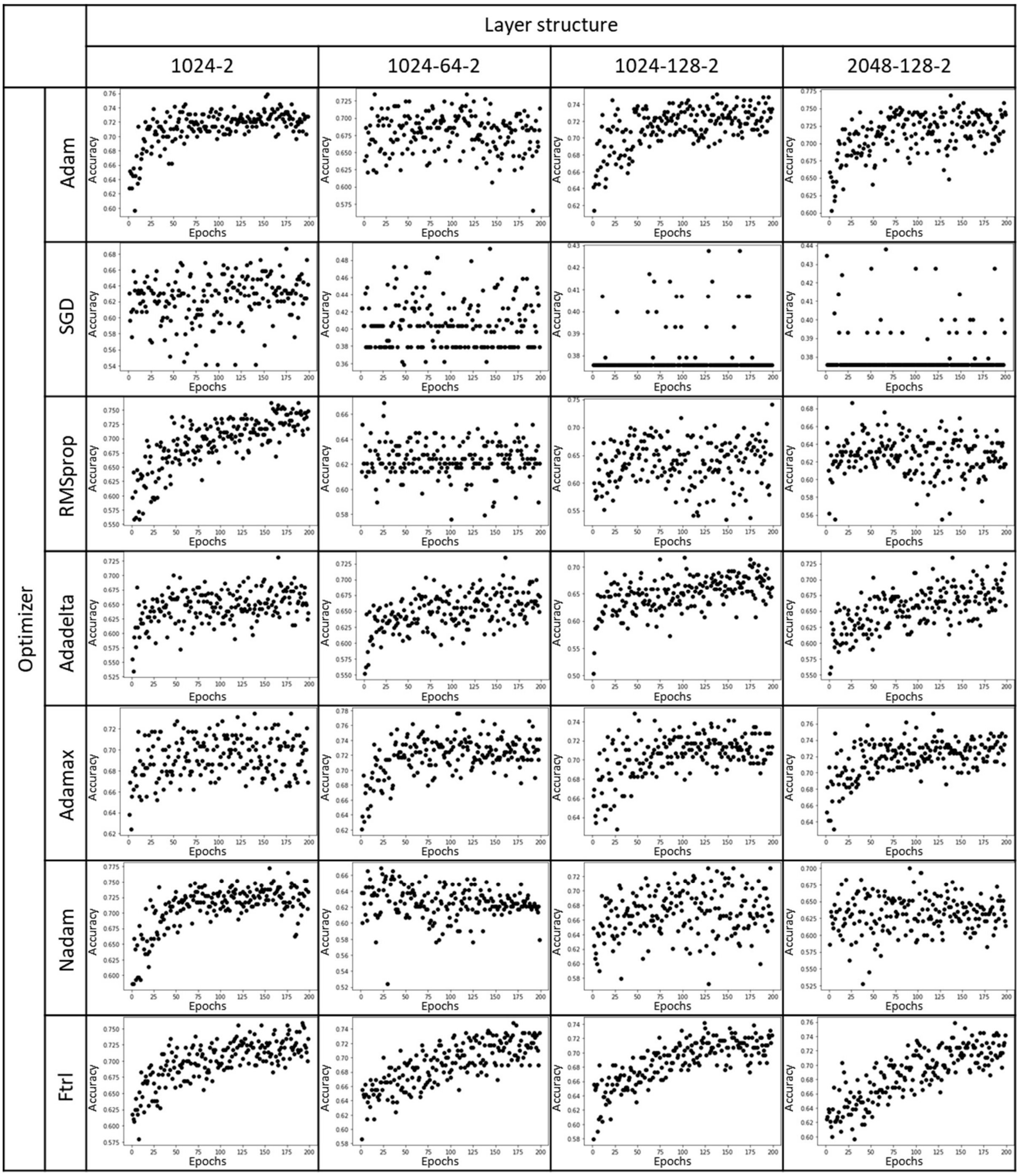
Layer structure
1024-2
1024-64-2
1024-128-2
2048-128-2
Optimizer
Adam
SGD
RMSprop
Adadelta
Adamax
Nadam
Ftrl
Accuracy
Epochs

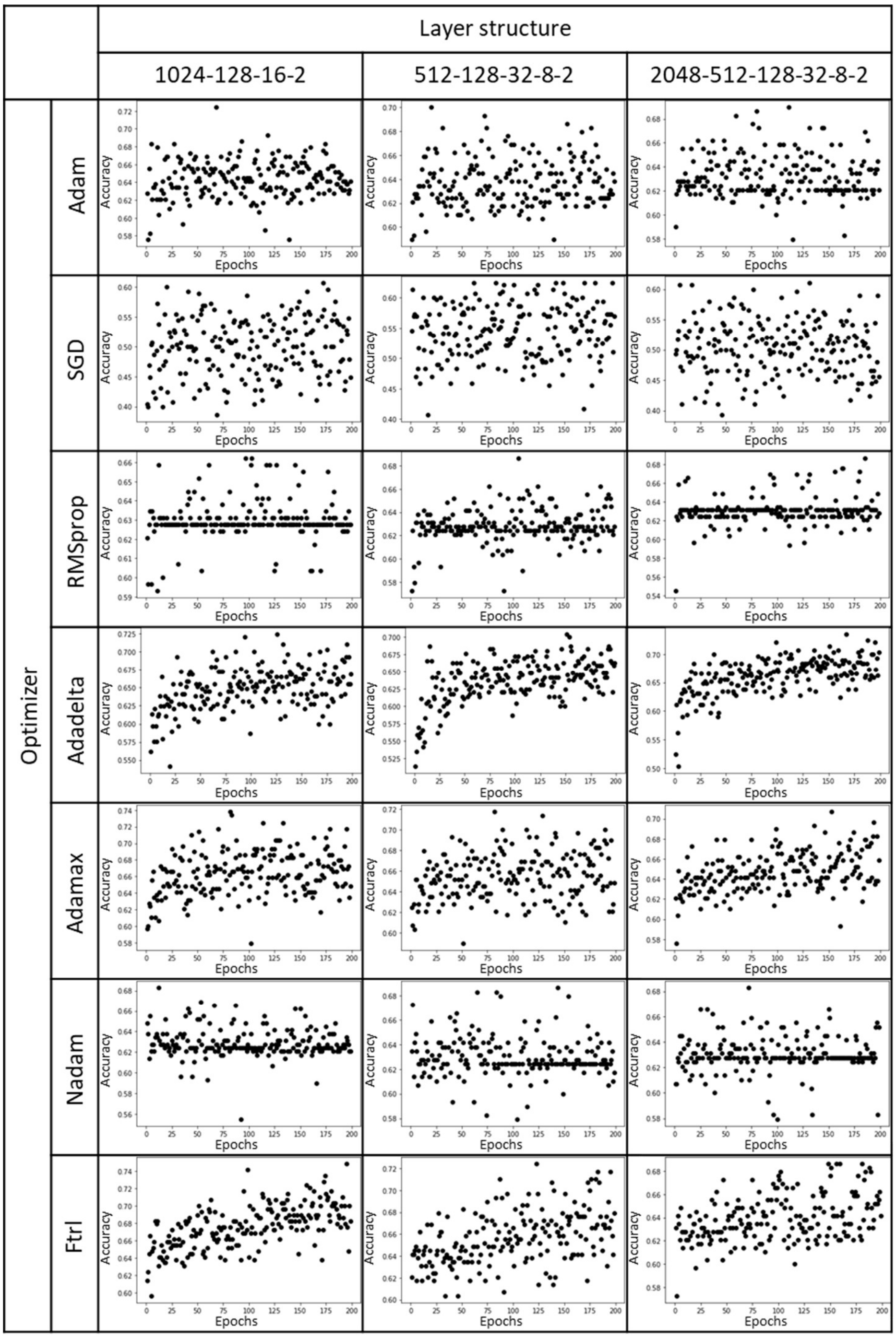

Figure S13: PPI prediction accuracy of candidate deep learning models for general PPI partner prediction trained with data from RosettaDock.

PPI prediction accuracy of 49 candidate models for general PPI partner prediction with 7 optimizers in the Keras library (Adam, SGD, RMSprop, Adadelta, Adamax, Nadam and Ftrl) and 7 candidate layer structures were analyzed with 1–200 epochs and plotted. Training data (iRMS and interaction energy) for deep learning were calculated using simulated docking structure from RosettaDock; related to Figure 5 and 6.

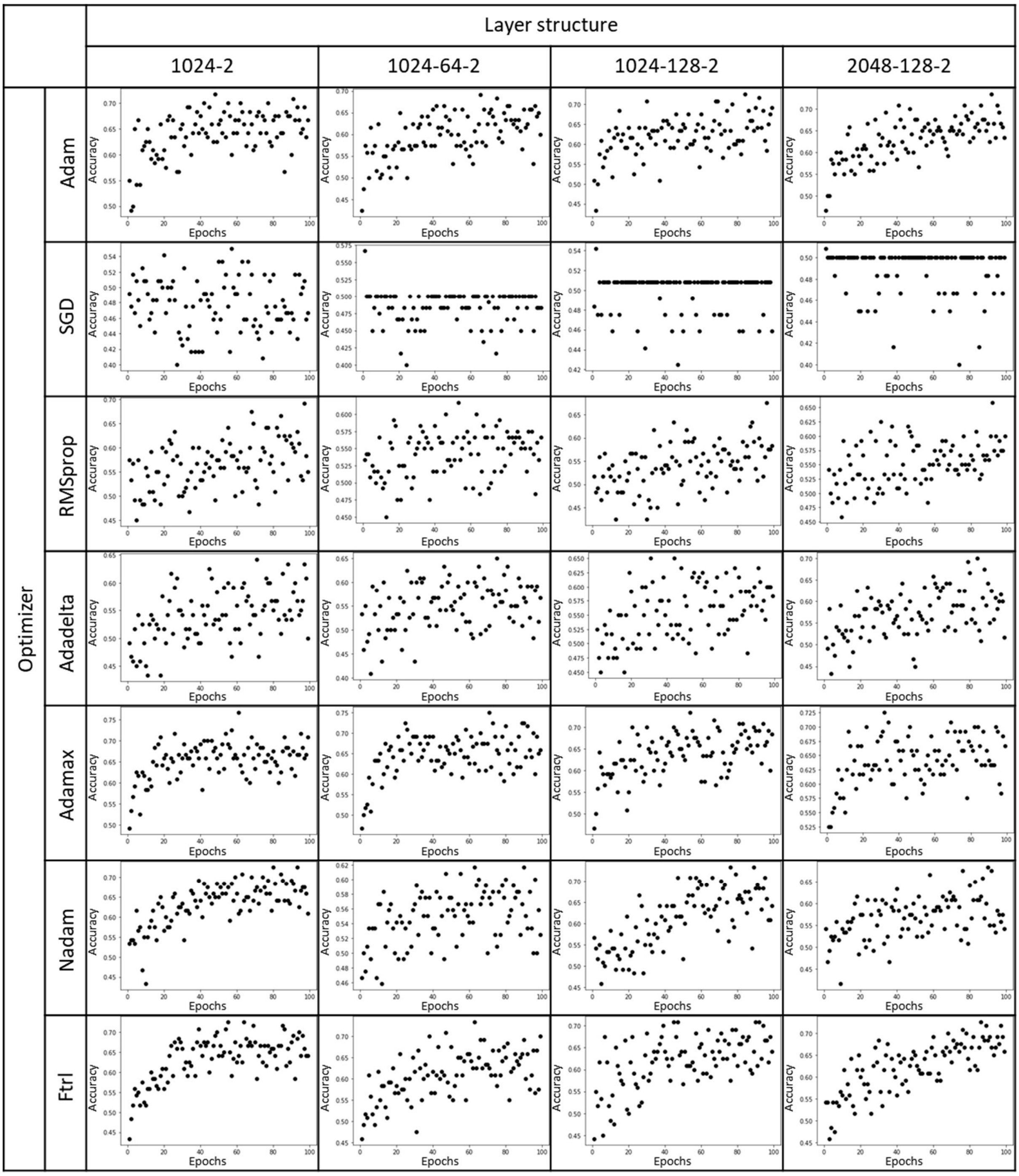
Layer structure
1024-2
1024-64-2
1024-128-2
2048-128-2
Optimizer
Adam
SGD
RMSprop
Adadelta
Adamax
Nadam
Ftrl
Accuracy
Epochs

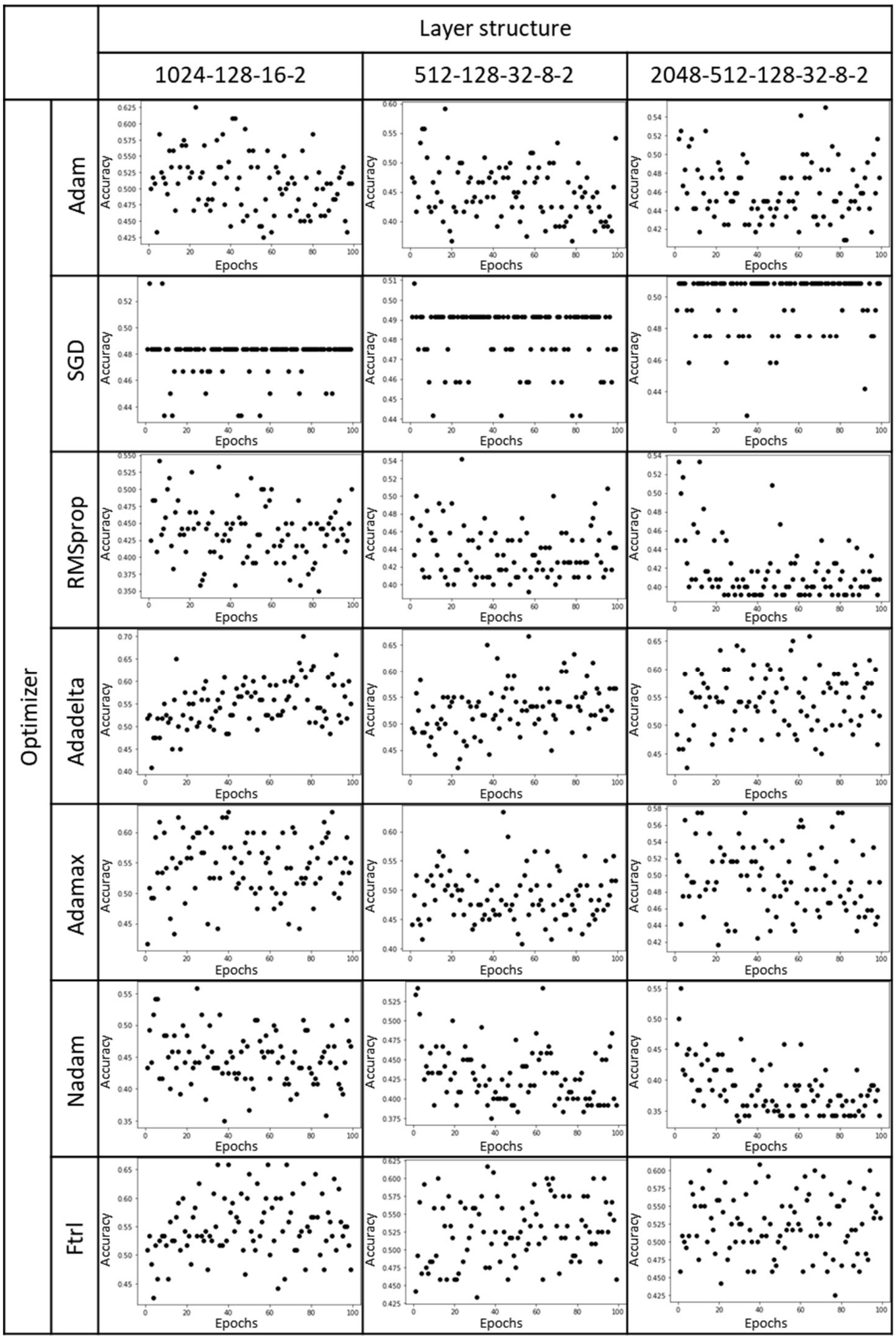

Figure S14: PPI prediction accuracy of candidate deep learning models for EUL substrate prediction trained with data from RosettaDock.

PPI prediction accuracy of 49 candidate models for EUL substrate prediction with 7 optimizers in the Keras library (Adam, SGD, RMSprop, Adadelta, Adamax, Nadam and Ftrl) and 7 candidate layer structures were analyzed with 1−100 epochs and plotted. Training data (iRMS and interaction energy) for deep learning were calculated using simulated docking structure from RosettaDock; related to Figure 5 and 6.

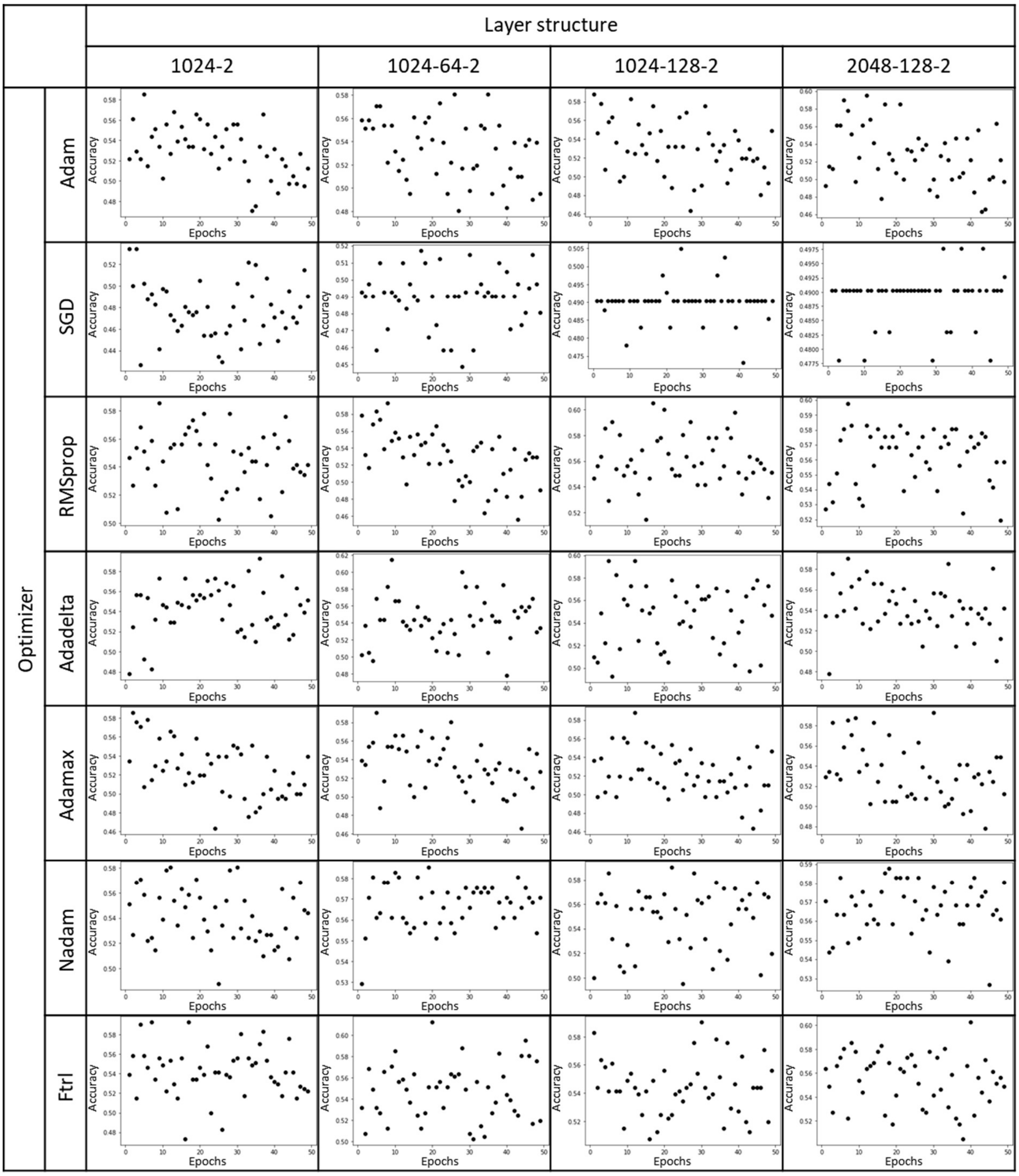

Layer structure
1024-2
1024-64-2
1024-128-2
2048-128-2
Optimizer
Adam
SGD
RMSprop
Adadelta
Adamax
Nadam
Ftrl
Accuracy
Epochs

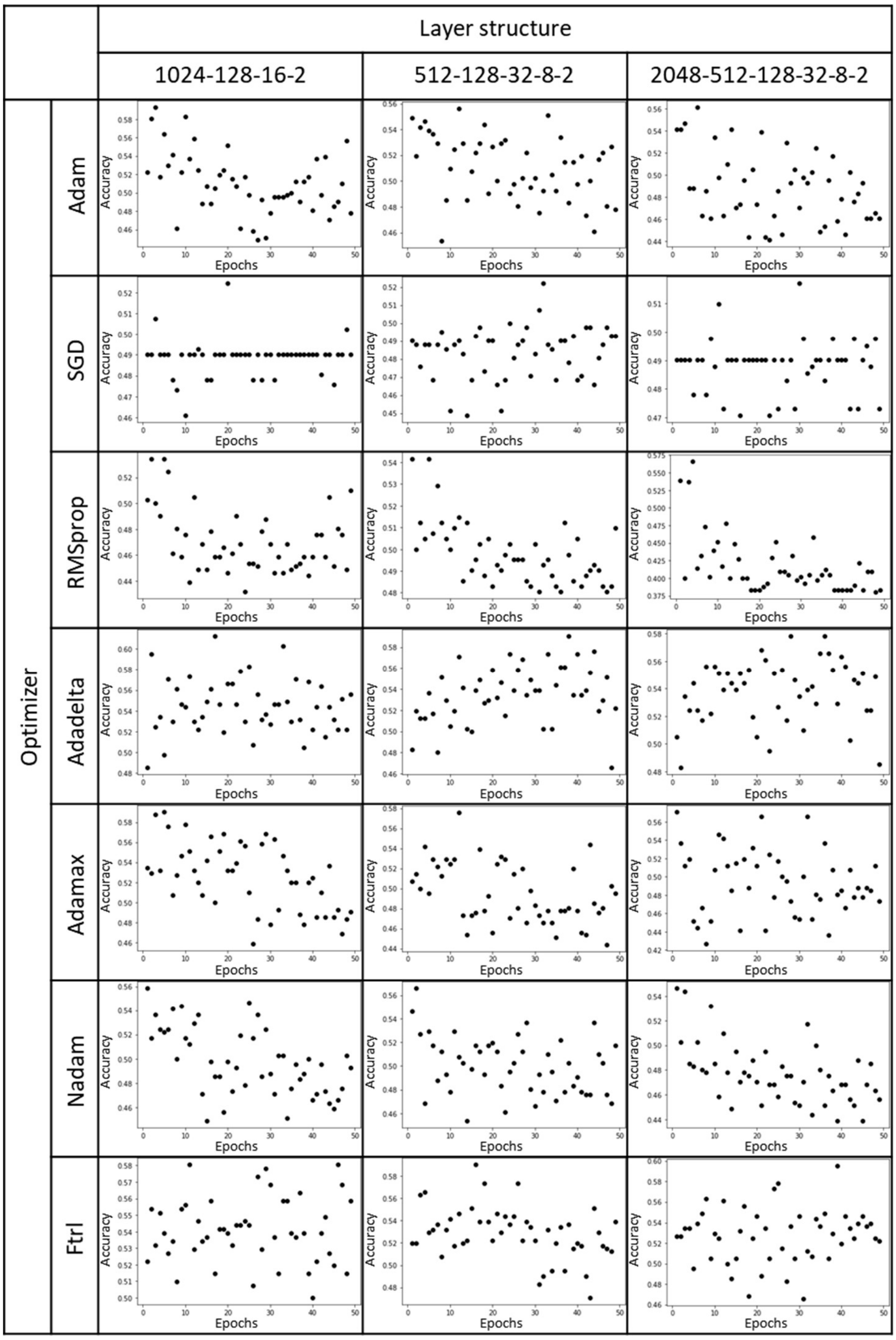

Figure S15: PPI prediction accuracy of candidate deep learning models for kinase substrate prediction trained with data from RosettaDock.

PPI prediction accuracy of 49 candidate models for kinase substrate prediction with 7 optimizers in the Keras library (Adam, SGD, RMSprop, Adadelta, Adamax, Nadam and Ftrl) and 7 candidate layer structures were analyzed with 1–50 epochs and plotted. Training data (iRMS and interaction energy) for deep learning were calculated using simulated docking structure from RosettaDock; related to Figure 5 and 6.

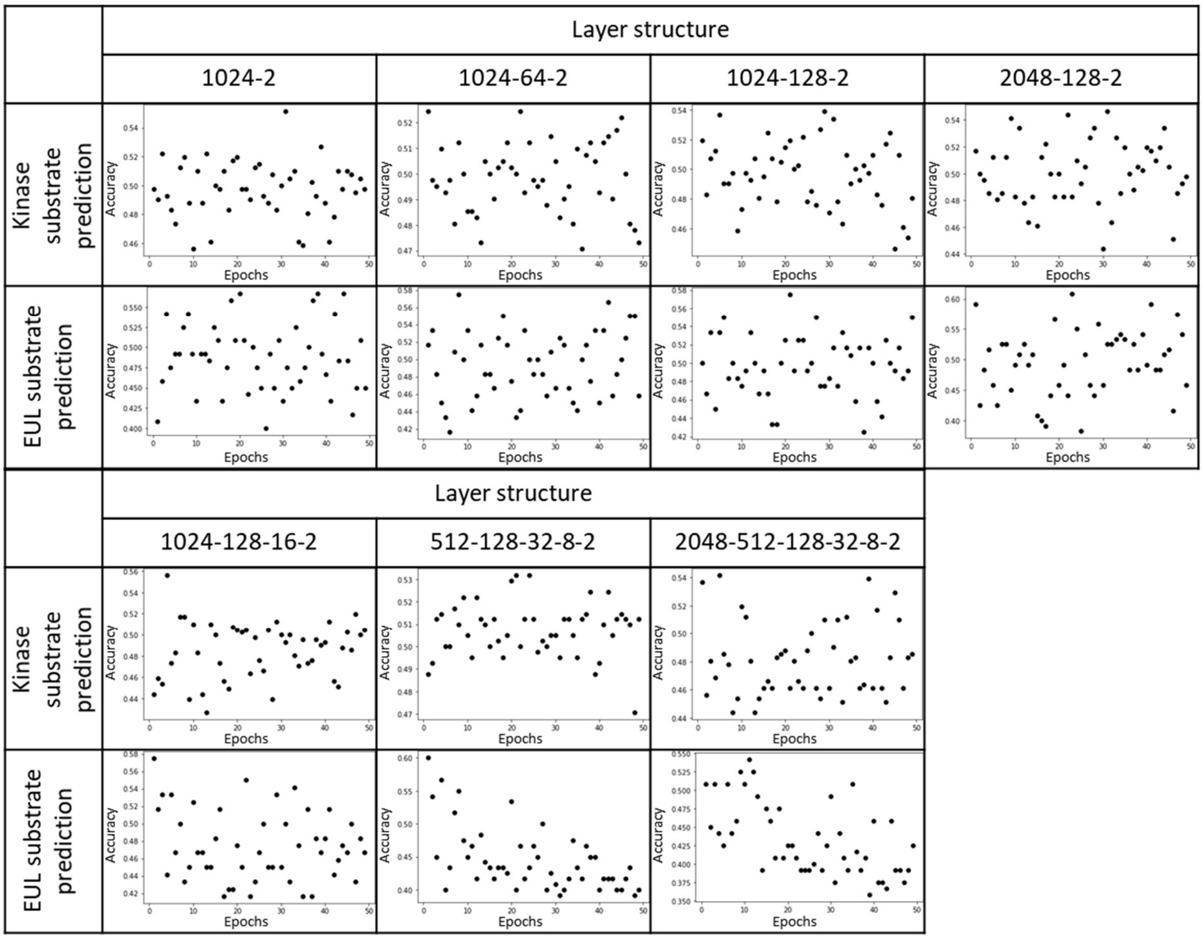

Figure S16: PPI prediction accuracy of candidate deep learning models for EUL and kinase substrate prediction trained with data from HDOCKlite.

PPI prediction accuracy of 49 candidate models for kinase substrate prediction with 7 optimizers in the Keras library (Adam, SGD, RMSprop, Adadelta, Adamax, Nadam and Ftrl) and 7 candidate layer structures were analyzed with 1−50 epochs and plotted. Training data (iRMS and interaction energy) for deep learning were calculated using simulated docking structure from HDOCKlite; related to Figure 5.

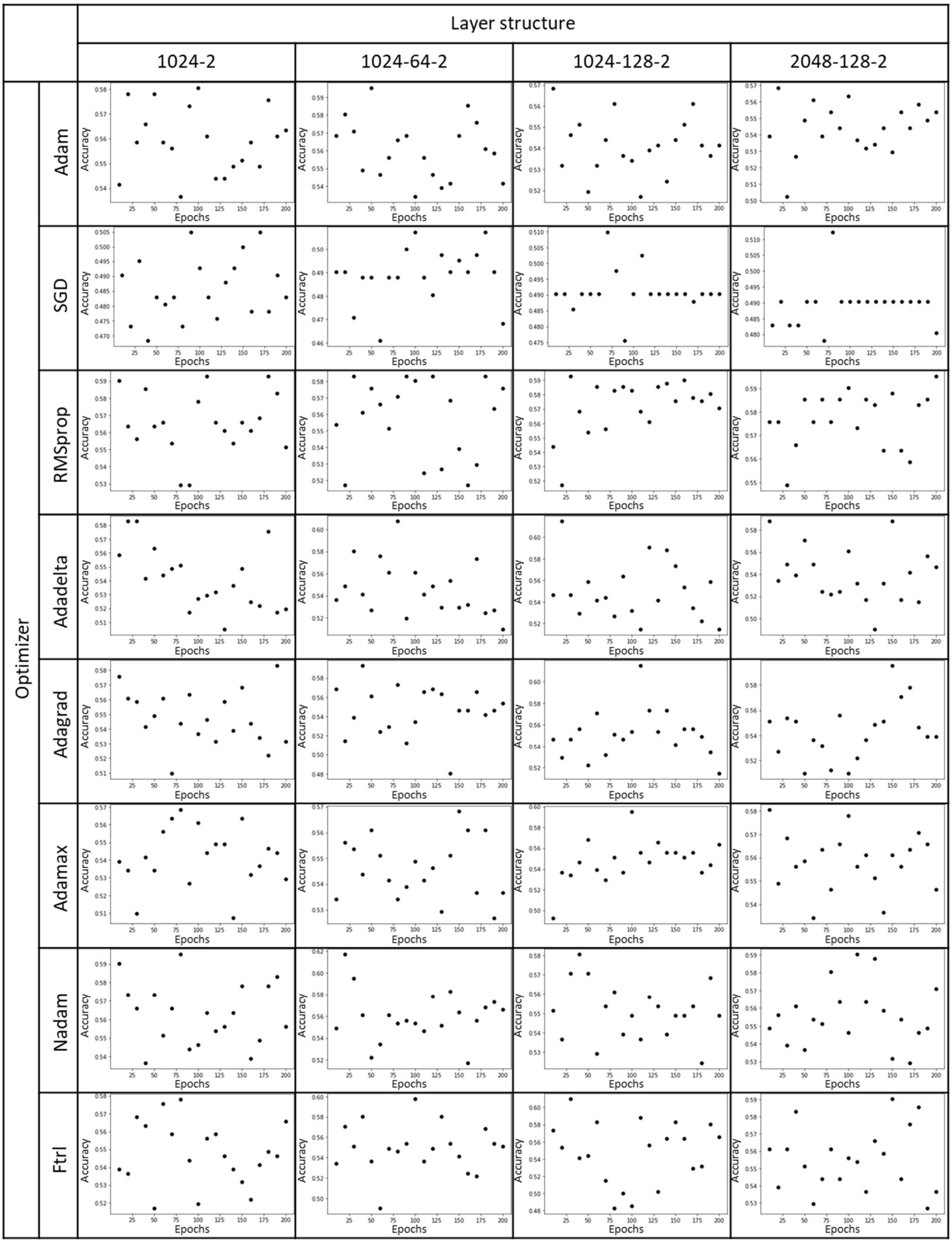
Layer structure
1024-2
1024-64-2
1024-128-2
2048-128-2
Optimizer
Adam
SGD
RMSprop
Adadelta
Adagrad
Adamax
Nadam
Ftrl
Accuracy
Epochs

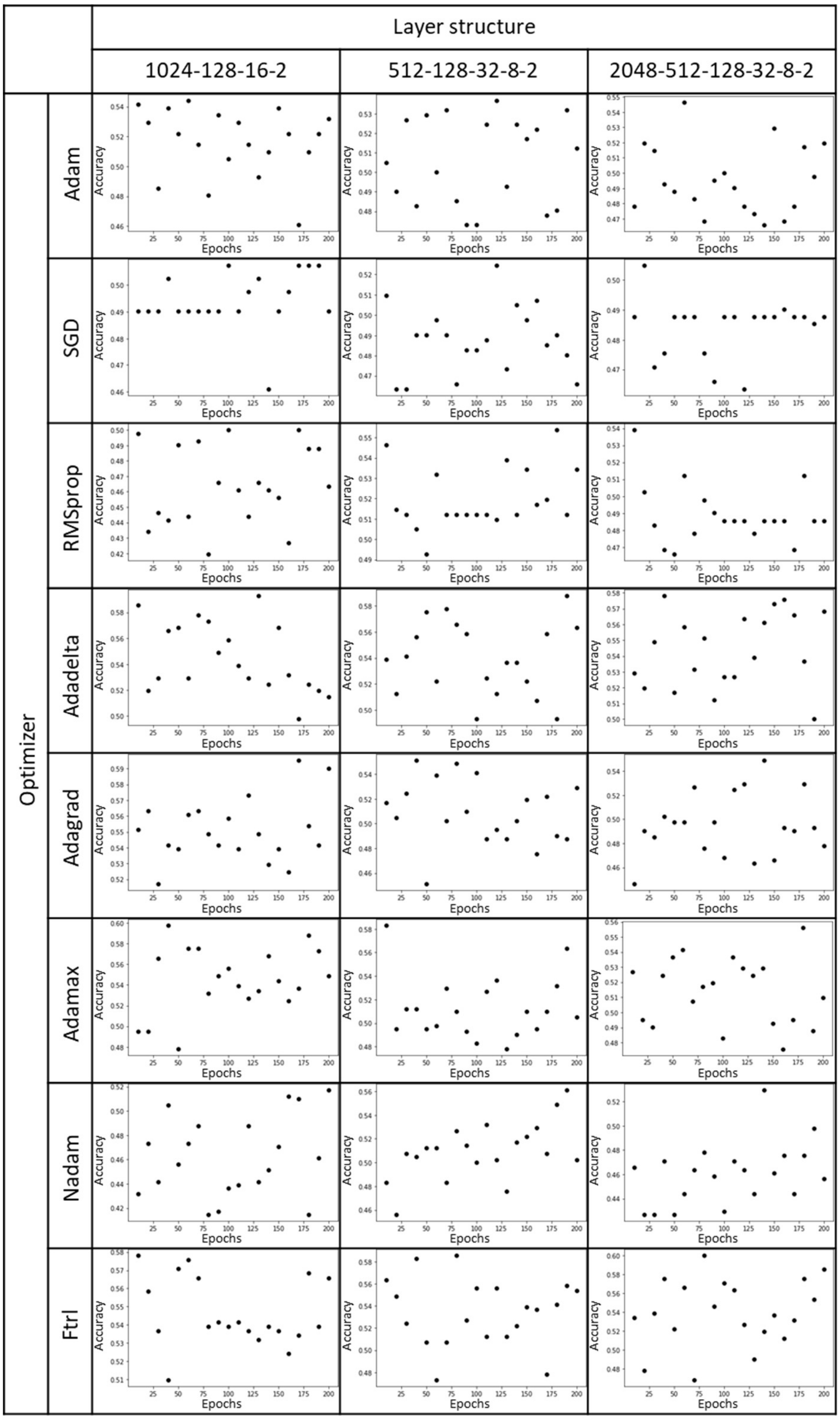

Figure S17: PPI prediction accuracy of candidate deep learning models for kinase substrate prediction trained with interaction energy, iRMS, rTM-score, iTM-score and riTM-score

PPI prediction accuracy of 56 candidate models for kinase substrate prediction with 8 optimizers in the Keras library (Adam, SGD, RMSprop, Adadelta, Adagrad, Adamax, Nadam and Ftrl) and 7 candidate layer structures were analyzed with 1–200 epochs in increments of 10 and plotted. As training data for deep learning, interaction energy, iRMS, rTM-score, iTM-score and riTM-score from 1,000 docking structures per kinase-substrate pair generatred using RosettaDock were used; related to Table 2 and Figure 7.

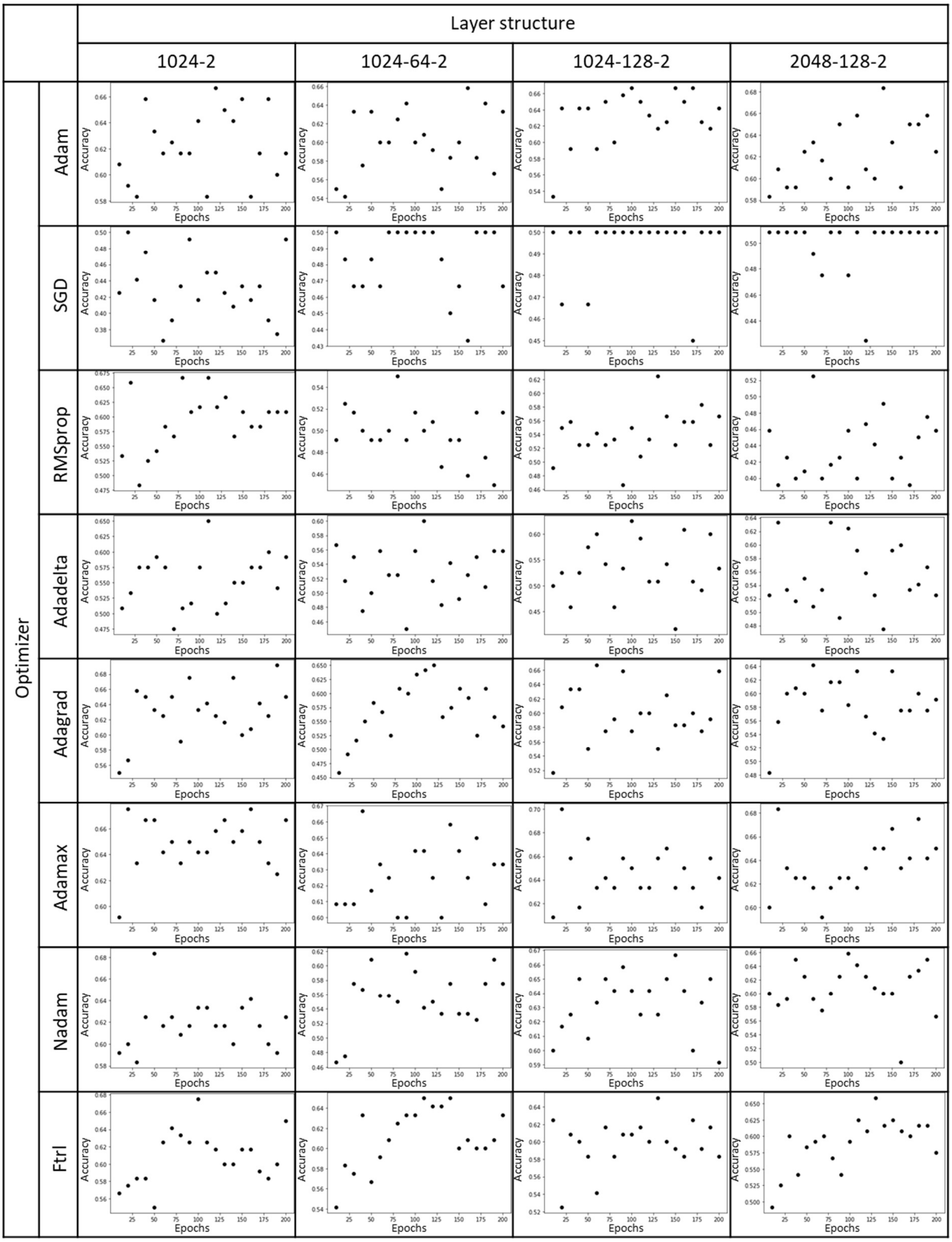

Layer structure
1024-2
1024-64-2
1024-128-2
2048-128-2
Optimizer
Adam
SGD
RMSprop
Adadelta
Adagrad
Adamax
Nadam
Ftrl
Accuracy
Epochs

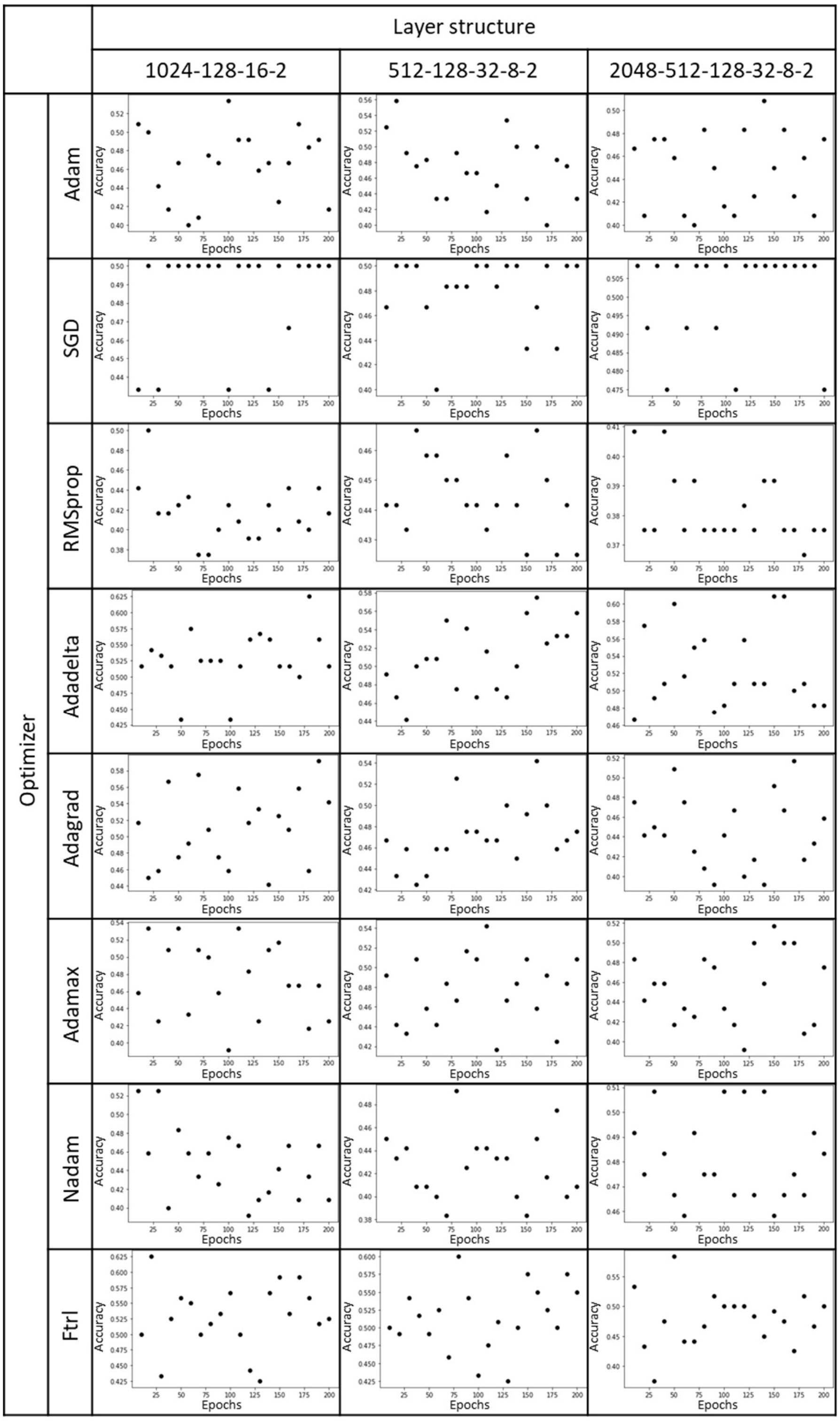

Figure S18: PPI prediction accuracy of candidate deep learning models for EUL substrate prediction trained with interaction energy, iRMS, rTM-score, iTM-score and riTM-score

PPI prediction accuracy of 56 candidate models for EUL substrate prediction with 8 optimizers in the Keras library (Adam, SGD, RMSprop, Adadelta, Adagrad, Adamax, Nadam and Ftrl) and 7 candidate layer structures were analyzed with 1–200 epochs in increments of 10 and plotted. As training data for deep learning, interaction energy, iRMS, rTM-score, iTM-score and riTM-score from 1,000 docking structures per EUL-substrate pair generated using RosettaDock were used; related to Table 2 and Figure 7.

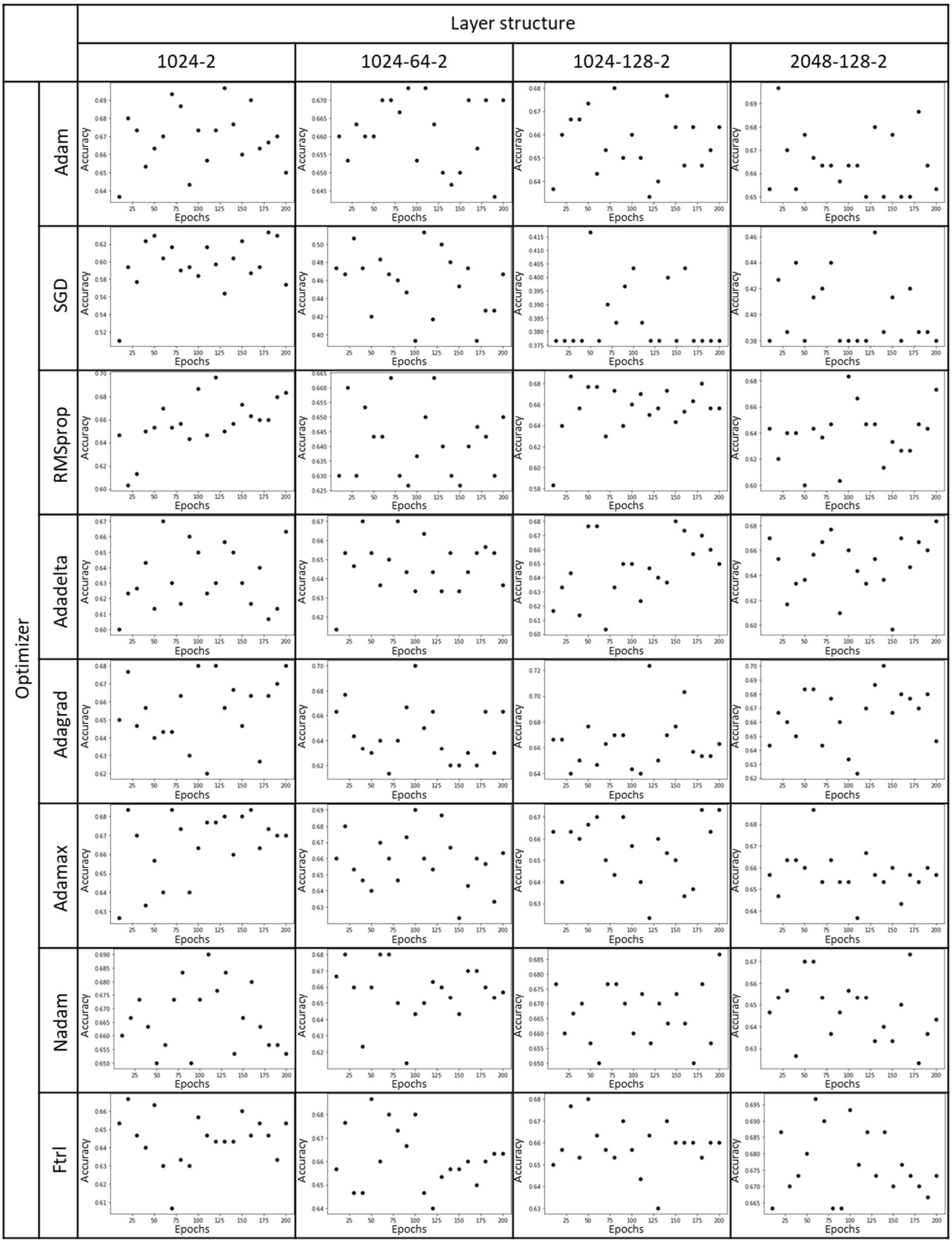

Layer structure
1024-2
1024-64-2
1024-128-2
2048-128-2
Optimizer
Adam
SGD
RMSprop
Adadelta
Adagrad
Adamax
Nadam
Ftrl
Accuracy
Epochs

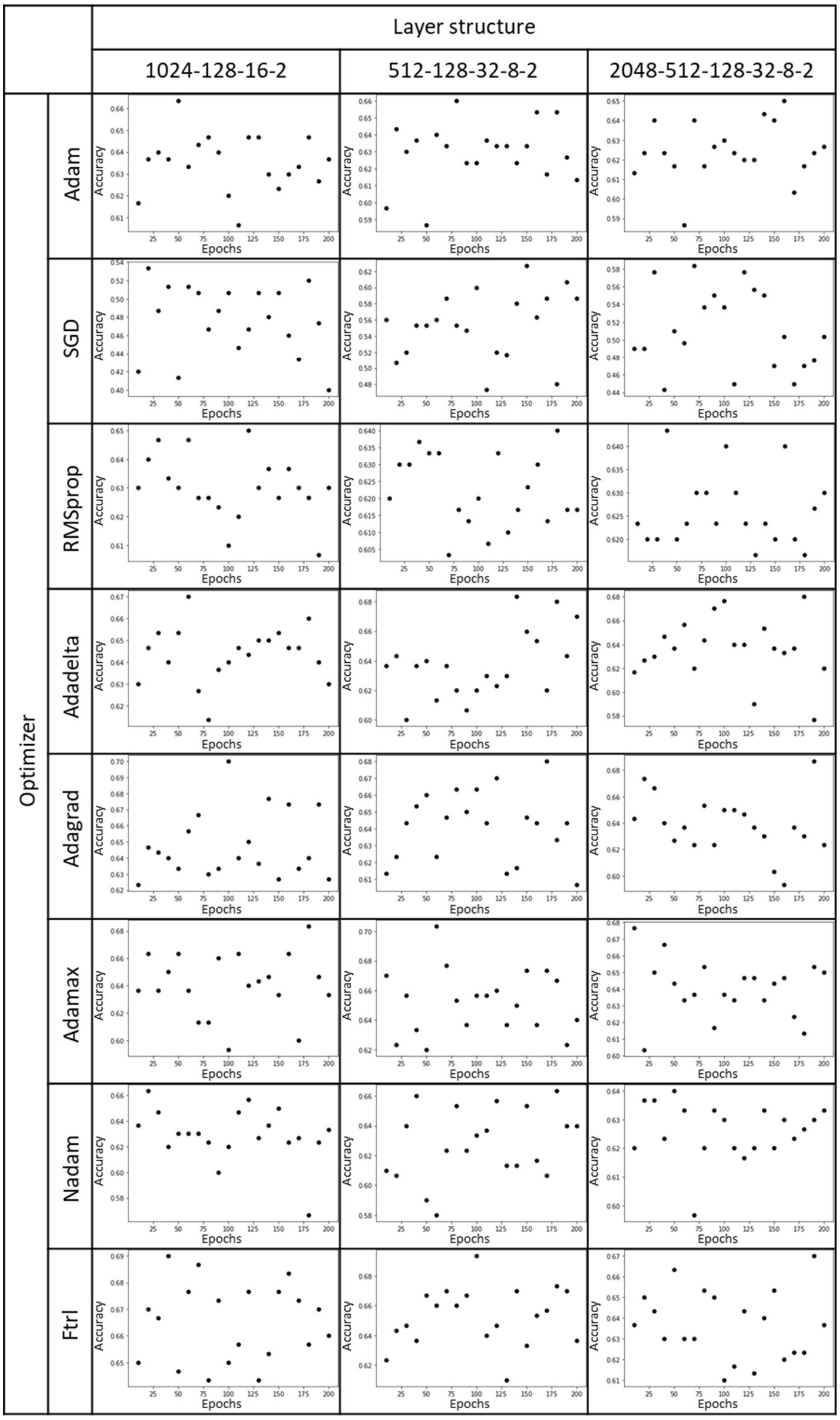

Figure S19: PPI prediction accuracy of candidate deep learning models for general protein interaction using experimentally determined PDB data trained with interaction energy, iRMS, rTM-score, iTM-score and riTM-score

PPI prediction accuracy of 56 candidate models for general protein interaction using experimentally determined PDB data with 8 optimizers in the Keras library (Adam, SGD, RMSprop, Adadelta, Adagrad, Adamax, Nadam and Ftrl) and 7 candidate layer structures were analyzed with 1–200 epochs in increments of 10 and plotted. As training data for deep learning, interaction energy, iRMS, rTM-score, iTM-score and riTM-score from 1,000 docking structures per protein complex generated using RosettaDock were used; related to Table 2 and Figure 7.

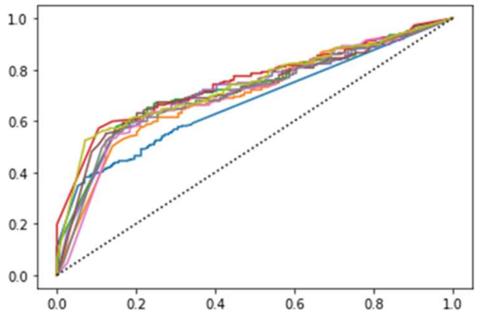

Line	Training Data	AUC
	Interaction energy	0.673
	Interaction energy+iRMS	0.701
	Interaction energy+rTM_score	0.726
	Interaction energy+iTM_score	0.752
	Interaction energy+riTM_score	0.719
	Interaction energy+iRMS+rTM_score	0.706
	Interaction energy+iRMS+iTM_score	0.71
	Interaction energy+iRMS+riTM_score	0.741
	Interaction energy+iRMS+rTM_score+iTM_score+riTM_score	0.724

Figure S20: Predictive performance of deep learning model for general protein interaction using experimentally determined PDB data trained with different scores including interaction energy, iRMS and modifications of TM-score

ROC curves from 10-fold cross validation when shuffling data in the deep learning model to predict general protein interaction using experimentally determined PDB data. Each line represents the ROC curve of a model trained with different training data, and the table provides the corresponding area AUC values; related to Table 2 and Figure 7.

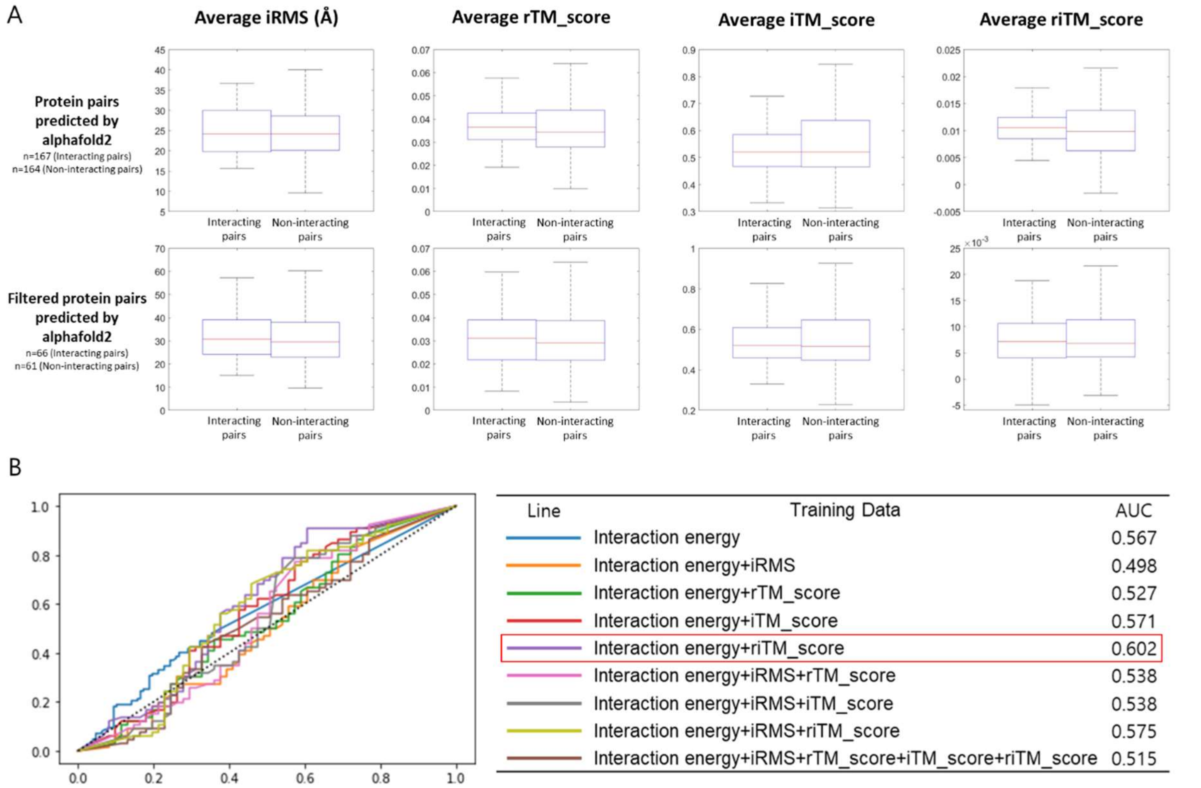

Figure S21: Protein interaction energy distribution analysis using general protein interacting pairs

(A) Distribution of average iRMSs, rTM-score, iTM-score and riTM-score of 1,000 docking structures of interacting predicted protein structure complexes and noninteracting predicted protein structure pairs are shown using box-and-whisker plot. (B) ROC curves from 10-fold cross validation when shuffling data in the deep learning model to prediction protein interactions using predicted protein structures. Each line represents the ROC curve of a model trained with different training data, and the table provides the corresponding area AUC values. The highest AUC value is highlighted with a red box; related to Table 2 and Figure 7.

Table S1. Criteria to distinguish narrow funnel-like interaction energy distribution of whole interaction pairs; related to Figure 1, S9 and 3.

	RosettaDock		HDOCKlite	
	Kinase pair	E3 ubiquitin ligase pair	Kinase pair	E3 ubiquitin ligase pair
Interaction energy criteria (kcal/mol)	-7.4	-15	178~400	344
iRMS criteria (Å)	52	36.8	49~49.4	41.4
Distribution criteria (%)	73.8	10.9	0.2	12.6

Table S2. Criteria to distinguish narrow funnel-like interaction energy distribution of interaction pairs with high-accuracy structure prediction results; related to Figure 1, S10 and 3.

	RosettaDock		HDOCKlite	
	Kinase pair	E3 ubiquitin ligase pair	Kinase pair	E3 ubiquitin ligase pair
Interaction energy criteria (kcal/mol)	-16.6	-14.6	50~73	344
iRMS criteria (Å)	12.6	54.4	57.6	41.4~42
Distribution criteria (%)	0.4	21	0.2	8.9
